# Aromatic scaffold-integrated hybrids of estradiol and benzoxazol-2-ones: synthesis and *in vitro* anticancer activity of *N*-substituted regioisomeric pairs†

**DOI:** 10.1039/d5ra01977j

**Published:** 2025-07-10

**Authors:** Ferenc Kovács, Ildikó Huliák, Hédi Árva, Marianna Kocsis, Mónika Kiricsi, Éva Frank

**Affiliations:** a Department of Molecular and Analytical Chemistry, University of Szeged Dóm tér 7-8 H-6720 Szeged Hungary frank@chem.u-szeged.hu +36-62-544-275; b Department of Biochemistry and Molecular Biology, Doctoral School of Biology, University of Szeged Közép fasor 52 H-6726 Szeged Hungary

## Abstract

Molecular hybridization often leads to derivatives with better pharmacokinetic properties and different biological effects than their parent compounds. Several *N*-substituted benzoxazolone – estradiol chimeras sharing a common benzene ring were prepared by *N*-alkylation or *N*-arylation *via* Chan–Lam cross-coupling of their pre-synthesized unsubstituted precursors. Both the 2,3- and 3,4-fused regioisomers were obtained in good yields and a comparison of the *in vitro* anticancer activity revealed useful structure–activity relationships. *N*-Substitution led to benzoxazolone derivatives with high cancer-selectivity. Moreover, the oxazolone ring fusion is more favorable at C2–C3 than at C3–C4 position, as well as the *N*-alkylation is preferable over *N*-arylation in terms of both drug-likeness and bioactivity. The most potent derivatives of the current set showed IC_50_ values between 2.0–5.3 μM on one (6b and 6d) or all of the tested malignant cell lines (6c) with excellent selectivity and triggered apoptosis in cancer cells. The strongest apoptosis-activating molecule was 6d.

## Abstract 2

Regioisomeric estradiol–benzoxazolone hybrids featuring a shared aromatic ring were efficiently synthesized, and their anticancer activities were comparatively evaluated. Derivatives bearing the 2,3-ring fusion exhibited enhanced biological activity.

## Graphical abstract

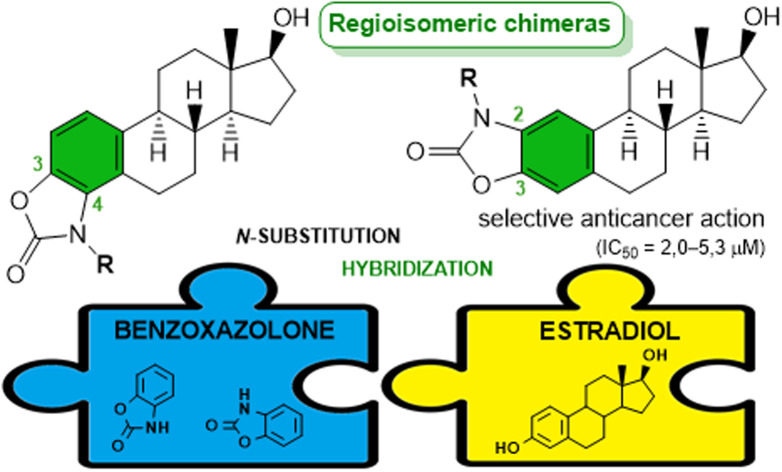

## Introduction

**Fig. 1:**
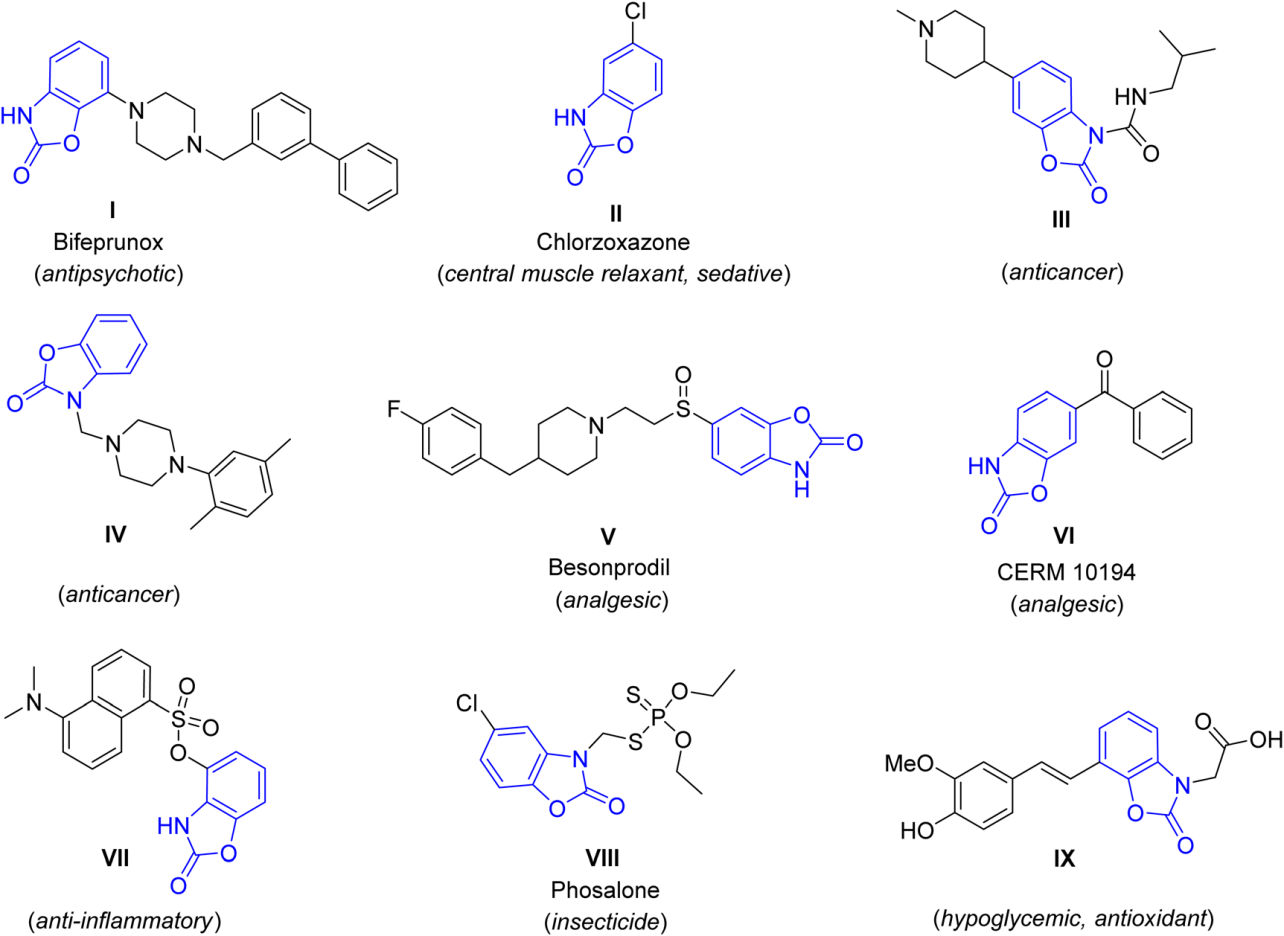
Selected benzoxazolone derivatives with diverse biological effects.

Benzoxazolone, a planar rigid heterocycle consisting of a benzene ring fused to a cyclic carbamate, represents an important structural motif in drug design and development.^1^ The advantageous physicochemical properties, the bioisosteric preference compared to groups more susceptible to metabolic conversion, the presence of both lipophilic and hydrophilic fragments within a single scaffold, and the extensive chemical modification possibilities make this molecule an outstanding pharmacophore in medicinal chemistry.^2^ The lipophilic benzene ring can be readily substituted by electrophiles,^3^ while the tautomerism of the hydrophilic carbamate moiety offers the possibility for *N*-substitution under slightly basic conditions.^4^ The oxazolone part of the molecule with two oxygen atoms as hydrogen bond acceptors and an NH as hydrogen bond donor site enables interaction with diverse macromolecular targets.^5^ Therefore, benzoxazolone constitutes the structural building block of several marketed drugs with a wide range of pharmacological effects (*e.g.* antipsychotic (I),^1^ sedative (II),^1,2^ anticancer (III,^2^IV^6^), analgesic (V,^2^VI^7^) anti-inflammatory (VII),^8^ insecticidal (VIII),^9^ antioxidant, hypoglycaemic (IX)^10,11^) as well as of drug candidates participating in clinical development pipelines (Fig. 1). Furthermore, benzoxazolone serves as bioisosteric surrogate for groups sensitive to enzymatic biotransformation, such as pyrocatechol and phenol.^2^ This enables the chemical modification of existing bioactive molecules in order to improve their pharmacokinetic profile.^12^

Hybridization of sex steroids, including estrogens^13,14^ with various heterocycles is a frequently used strategy for the synthesis of novel derivatives possessing anticancer activity,^15^ especially in cases where the macromolecular target has not yet been identified and rational design and optimization based on docking studies are therefore not feasible. According to this concept, different pharmacophores with beneficial properties are connected through a linker or even by integrating common structural moieties (*e.g.* a ring) to obtain a new derivative.^16^ The purpose of hybridization is to maintain or possibly strengthen favourable parameters (synergism) in order to create new, hitherto unknown active substances.^17^ Highly lipophilic steroids with good cell membrane penetration should therefore be combined primarily with hydrophilic units (heterocyclic scaffolds) so that the produced active ingredient has an even better pharmacokinetic profile.

Bioisosteric replacement of the phenolic or catechol A-ring of estradiol or its *in vivo* metabolites, such as 2- and 4-hydroxyestradiols,^18^ with a benzoxazolone ring can significantly alter the biological effect while improving the stability and bioavailability of the molecule. In addition to participating in rapid metabolism, the phenolic OH group at C-3 of these derivatives also plays a crucial role in binding to the estrogen receptor *via* hydrogen bond, mainly as a H-donor.^19^ Consequently, the incorporation of a 2,3- or 3,4-fused oxazolone ring and thus the transformation of the H-donor 3-OH group into a ring-forming H-acceptor oxygen, as well as the simultaneous adjacent (C-2 or C-4) substitution makes it more likely that the compound is free of hormonal effects, while retaining the possibility of interaction with other macro-molecular targets. Several C-2 substituted estradiol derivatives are known to exhibit anticancer activity, including those containing an intact 3-OH group (*e.g.* 2-methoxyestradiol, 2ME2)^20,21^ as well as those bearing 3-OR and 3-NHR functionalities^15,22^ however, C-4 substituted derivatives are less studied.^23^

The construction of a benzoxazolone ring can be achieved from either primary or secondary 2-aminophenols through a simple ring closure with phosgene, triphosgene, or the less toxic carbonyldiimidazole (CDI). In the former case, the NH of the primarily formed cyclic carbamate can be substituted further with alkyl or aralkyl halides under basic conditions,^24^ while in the latter case, cyclization of the *N*-substituted amine obtained by the reductive alkylation of aminophenol leads to the desired product.^25^ Interestingly, only a few examples for the *N*-arylation of the benzoxazolone ring are to be found in the literature,^26^ despite the fact that copper-promoted Ullmann–Goldberg reaction with aryl halides^27,28^ or its modified version, the Chan–Lam cross coupling of tautomerizable heterocycles^29^ using boronic acids to obtain novel N–C bonds has long been well known.

Previously we developed a two-step method for the synthesis of 2- and 4-aminoestradiols involving the mononitration of estrone without the formation of a 2,4-disubstituted product, followed by simultaneous reduction of both the NO_2_ and 17-(C=O) groups after separation of the regioisomeric pair. Cyclization of the aminophenols with CDI yielded A-ring-fused, *N*-unsubstituted oxazol-2-ones.^30^ Since these derivatives exhibited significant cytotoxic effects on certain cancer cell lines, our present goal was to synthesize additional derivatives by *N*-alkylation and *N*-arylation of the benzoxazolone moiety in order to map and compare the biological effects of products with different ring fusion and substitution patterns. The cytotoxicity of all synthesized derivatives was tested *in vitro* by the colorimetric 3-(4,5-dimethylthiazol-2-yl)-2,5-diphenyltetrazolium bromide (MTT) assay^31^ using human A549 (lung), DU-145 (hormone insensitive prostate), HeLa (cervical), MCF-7 (breast) cancer cell lines and non-cancerous MRC-5 fibroblast cells in comparison with the structurally most similar 2ME2 and the clinically used cisplatin. Based on the primary toxicity screen, three *N*-alkylated regioisomeric pairs were selected and subjected to further experiments to obtain their IC_50_ values on different cell lines and to validate their apoptosis-inducing potential against cancer cells. The physicochemical, ADME, and drug-likeness parameters of the synthesized novel compounds were also computed using the freely accessible SwissADME online web tool.^32^

## Results and discussion

### Synthetic studies and *in silico* prediction of drug-likeness

**Scheme 1:**
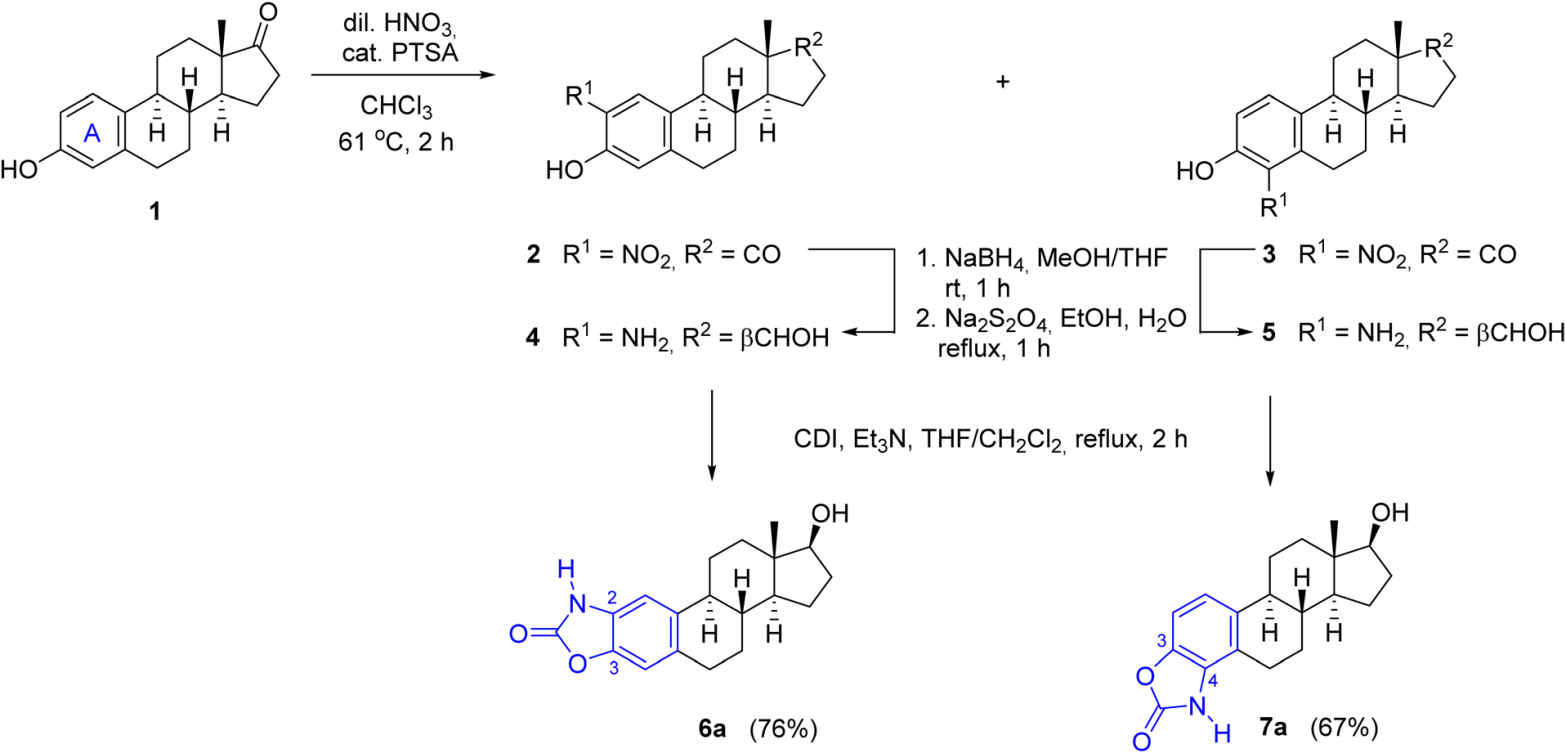
Modified synthesis of A-ring-integrated *N*-unsubstituted benzoxazolone regioisomers.

For the synthesis of A-ring fused oxazol-2(3*H*)-ones, 2- and 4-aminoestradiols (4 and 5) were first prepared from estrone (1) with a slight modification of the previously applied nitration/reduction sequence.^30^ Nitration of 1 was carried out with an equivalent amount of diluted nitric acid in chloroform instead of dichloromethane at reflux temperature due to the better solubility of 1 in this solvent with higher boiling point. A catalytic amount of *p*-toluenesulfonic acid (PTSA) completed the conversion, presumably by promoting the formation of the electrophilic nitronium ion. As a result of the aromatic substitution, only mononitro compounds (2 and 3) were obtained with almost identical yields (49 and 47%). During scale-up synthesis, recrystallization from acetic acid was used to separate the regioisomers thanks to the much poorer solubility of 4-nitroestrone (3) in this solvent. Although the following reduction of both NO_2_ and C=O groups can be carried out in one step by applying NaBH_4_, Pd/C in a protic solvent,^30^ the resulting aminophenols 4 and 5 can easily undergo oxidation in alkaline medium,^33^ requiring an immediate work-up by acidification after completion of the reaction. Alternatively, the reduction could also be performed in two steps, involving a carbonyl reduction with sodium borohydride and a subsequent treatment with sodium dithionite in aqueous EtOH to afford compounds 4 and 5 in almost the same yields as in the one-step procedure. Incorporation of a carbonyl group by CDI led to the unsubstituted cyclic carbamates 6a and 7a,^30^ which served as precursors for the *N*-alkyl and N-(hetero)arylbenzoxazolones (Scheme 1).

Next, the alkylation reaction of 6a was carried out using iodomethane (for 6b), bromoethane (for 6c) 1-bromopropane (for 6d) or 2-iodopropane (for 6e) in the presence of a base with gentle heating (Table 1). During the transformations, complete conversion of the starting material was observed in all cases after 2 h, and the products (6b–e) were isolated in good yields (78–86%) (entries 1–4). Subsequently, *N*-(hetero)aryl derivatives (8a–g) were planned to be prepared by Chan–Lam cross-coupling, which typically occurs even at room temperature in the presence of pyridine or bidentate ligands, such as 2,2′-bipyridine or 1,10-phenanthroline.^34,35^ Traditionally, chlorinated solvents are used,^36^ although reactions in DMSO, DMF or MeCN are also common.^37^ During the model reaction of 6a with phenylboronic acid carried out initially in DMSO or DMF, conversion was not observed. After changing the solvent to EtOH, however, the reaction was completed within 24 h at room temperature to give 8a in 81% yield after chromatographic purification (Table 1, entry 5). The success of the solvent exchange can be explained by the following reasons. On the one hand, in the case of EtOH as a solvent, the diethyl ester can also be formed from the used boronic acid, which is known to be almost 20 times more reactive than the corresponding boronic acid during the transmetallation step.^38^ On the other hand, during the reaction, the complex formed from the copper(ii) salt and EtOH is able to coordinate the boronic acid through the non-bonding electron pair of the oxygen and thus can promote the transmetallation step.^34^ Extending the cross-coupling to other (hetero)arylboronic acids, we obtained the products (8b–g) in moderate to good isolated yields (67–88%) after chromatographic purification (Table 1, entries 6–11).

**Table 1:** Synthesis of A-ring 2,3-fused *N*-substituted oxazol-2-ones

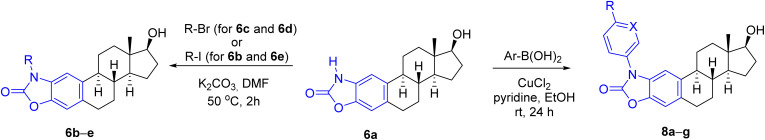				
Entry	*N*-Substituted benzoxazolone	R	X	Yield (%)
1	6b	Me	—	84
2	6c	Et	—	86
3	6d	Pr	—	78
4	6e	i-Pr	—	83
5	8a	H	CH	81
6	8b	F	CH	80
7	8c	Cl	CH	74
8	8d	Br	CH	88
9	8e	CN	CH	67
10	8f	Et	CH	84
11	8g	H	N	69

Since drug-likeness, which refers to the chance of a molecule becoming an oral drug in terms of bioavailability, is routinely employed to screen chemical libraries to exclude molecules with properties that are unlikely to be compatible with an acceptable pharmacokinetic profile, a rapid estimation for the synthesized compounds was performed using the SwissADME web tool^32^ (ESI). The prediction showed that only *N*-unsubstituted (6a) and alkylated oxazolones (6b–e) met all criteria for drug-likeness, while *N*-aryl derivatives (with the only exception of 8g) violated one or more RO5 (ESI, Table S2†) due to their high lipophilicity (XLOGP3 > 5) and poor water solubility (ESI, Table S1†). According to the computational results, the *N*-unsubstituted (6a) and *N*-alkyl-substituted cyclic carbamates (6b–e) showed high gastrointestinal (GI) absorption (Table S2†) and moderate solubility (Table S1†), which indicate that these compounds with optimal lipophilicity (log *P* < 5 according to all filters) can achieve good bioavailability when administered orally. It was also observed that all of them have a potential to penetrate through the blood–brain barrier (BBB), which may be a cause of concern for possible CNS-related side effects. However, since the calculation method does not quantify the extent of permeation, the amount entering may not be significant enough to have a pronounced toxic effect. It should be noted that *in silico* models of BBB penetration often do not fully reproduce the complexity of human BBB, resulting in limited predictive accuracy and possible false positive or negative results. Among the non-aryl substituted derivatives, compounds 6a and 6b were predicted to be a substrate of P-glycoprotein (P-gp), which usually acts as an efflux transporter, pumping xenobiotics back into the GI lumen, thereby reducing the plasma and tissue concentrations of a drug. It is also important to emphasize that none of the compounds contain structurally promiscuous (PAINS alert) or putatively toxic, chemically reactive unstable moieties (Brenk alert).

Considering the predicted poor water solubility of the *N*-aryl derivatives and because, based on the preliminary biological tests, none of 8a–g exerted any particular biological effect (see later) presumably due to steric reasons preventing their binding to the target, in the case of steroidal oxazol-2(3*H*)-one 7a, only the *N*-alkylation reactions were performed (Table 2), thus obtaining the regioisomeric pairs (7b–e) of derivatives 6b–e.

**Table 2:** Synthesis of A-ring 3,4-fused *N*-substituted oxazol-2-ones

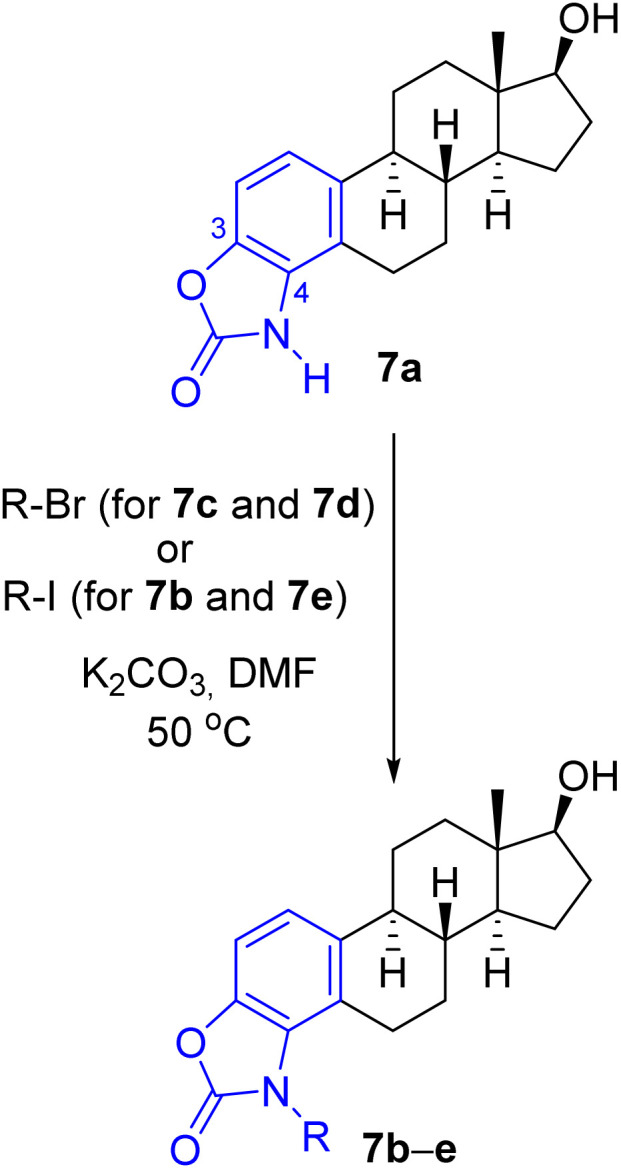				
Entry	7	R	Reaction time (h)	Yield (%)
1	b	Me	2	75
2	c	Et	2	81
3	d	Pr	8	76
4	e	i-Pr	20	53

The products were generally obtained with lower yields than 6b–e, and an increased reaction time had to be used to introduce longer or branched carbon chains (entries 3 and 4).

The lowest yield was achieved for the derivative containing the isopropyl group (7e), which can be attributed to steric reasons (Table 2, entry 4). *In silico* ADME screening and drug-likeness assessment of the heterocyclic compounds fused to the A-ring at C3–C4 position (7a–e) led to similar results as in the case of the 2,3-fused regioisomeric pairs (6a–e) (Tables S1 and S2, ESI†). The only significant difference between the two series of compounds lies in the predicted interaction with P-gp, as the program identified not only derivatives 7a and 7b, but also the *N*-ethyl-substituted compound 7c as a substrate for P-gp, in contrast to 6c. Based on all computed data, compounds 6c–e, 7d and 7e were found to be promising candidates from the current set.

The structures of all the newly synthesized *N*-substituted steroidal oxazolones (6b–e, 7b–e and 8a–g) were confirmed by ^1^H and ^13^C NMR spectroscopy as well as by MS measurements. The most important difference between the 2,3-fused (6, 8) and 3,4-fused compounds (7) is that in the former case the C-1 and C-4 protons give a singlet signal in the ^1^H NMR spectrum, while in the latter case the protons on the adjacent C-1 and C-2 appear as doublets with the same coupling constant. The negative carbon signal of the C=O group of the heteroring can be seen between 152.2 and 154.7 ppm in the ^13^C NMR spectra (APT) for the differently substituted derivatives, and the signals of the incorporated alkyl or hetero(aryl) moieties can also be identified.

### Pharmacological studies of the synthesized compounds

**Fig. 2:**
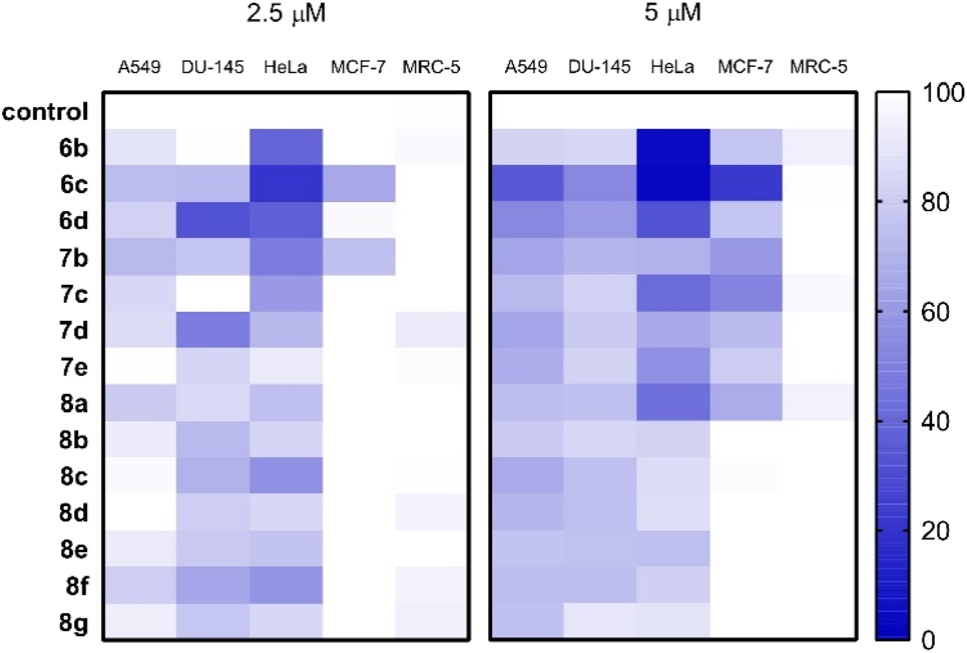
The primary cytotoxic effect of the synthesized *N*-substituted benzoxazolones on various human cancerous cell lines and on non-cancerous MRC-5 fibroblasts shown on heat maps (each compound was applied on cells in a concentration of *c* = 2.5 μM – left panel, and 5 μM – right panel; the incubation time was in each case 72 h, the tested cell lines are A549 lung adenocarcinoma, DU-145 prostate adenocarcinoma, HeLa cervical adenocarcinoma, MCF-7 breast adenocarcinoma cancer cell lines and non-cancerous MRC-5 lung fibroblast cells). Control represents the viability of untreated cells. The heat map illustrates the viability of cells treated with the test compounds individually compared to the viability of the untreated control cells, expressed in percentages. The cytotoxic activity of each compound correlates with the intensity of blue colour on the heat map. White colour represents 100% viability.

Cancer cell-specific growth inhibition of the *N*-substituted benzoxazolones was assessed by applying the compounds in 2.5 and 5 μM concentrations on various human cells, and their effect on the viability of A549 lung, DU-145 prostate, HeLa cervical, MCF-7 breast adenocarcinoma cells as well as on the non-cancerous MRC-5 fibroblasts were screened. The resulting viability data are shown as heat maps (Fig. 2 and Table S3, ESI†). The solution of compound 6e prepared in DMSO was not found to be homogeneous and clear enough for cellular studies, and thus it was excluded from pharmacological screening. The results demonstrate that overall, MCF-7 cells were the least, whereas HeLa cells were the most sensitive to the anti-proliferative effects (represented as viability in %) of the test compounds. Most of the molecules were effective both at 2.5 as well as at 5 μM concentrations, and in most cases, a concentration-dependent anti-cancer activity could be observed. Importantly, non-cancerous fibroblasts were not or just mildly affected by the test compounds indicating a strong cancer-selective performance (Fig. 2 and Table S3, ESI†). When the toxicity of each molecule was considered individually, among the *N*-substituted benzoxazolones compounds 6b, 6c and 6d were the most promising ones. Molecule 6c was especially effective, as it exerted a strong killing activity on all cancer cell lines tested, however, it did not affect non-cancerous fibroblasts. Based on the prominent cancer-selective performance at both concentrations of the screen, compounds 6b, 6c and 6d were regarded as positive hits. It is also apparent that the A-ring 2,3-fused *N*-alkylated oxazolones performed better than their identically substituted 3,4-fused counterparts (6b–d*versus*7b–d). Moreover, the N-(hetero)aryl compounds (8a–g) manifested only moderate anti-cancer potential, significantly weaker than that of 6b, 6c and 6d, suggesting a structure–activity correlation favouring 2,3-fused *N*-alkylated oxazolones.

Given the results of the primary toxicity screens, the half-maximal inhibitory concentration values of compounds 6b–d and 7b–d were assessed on A549, DU-145, HeLa, MCF-7 and MRC-5 cells (Table 3). The MTT cell viability data and the obtained dose–response curves (Fig. S1, ESI†) provided the basis for the determination of half maximal inhibitory concentration (IC_50_) values, which were compared with those of unsubstituted molecules (6a, 7a), 2ME2 and cisplatin on these cell lines, published previously.^30^ IC_50_ is a measure of the potency of a substance, it indicates how much of a particular inhibitory substance (*e.g.* drug) is needed to inhibit, *in vitro*, a given biological process or biological component by 50%. As it was expected based on the primary screens, compound 6c exhibited the lowest IC_50_ values on all of the cancerous cells and showed no toxicity on fibroblasts, representing clearly the most efficient molecule from the library. Actually, this molecule performed equally well as cisplatin or 2ME2 on the tested cancer cells. Furthermore, on HeLa cells compounds 6b and 6d produced similarly low IC_50_ as 6c, nevertheless somewhat higher values were obtained when 6b and 6d were administered to A549 and DU-145 cells. HeLa cells were effectively eliminated also by 7c as well as by the unsubstituted cyclic carbamates (6a, 7a) (Table 3). On the other hand, only 6c resulted to be detrimental to MCF-7 cells highlighting its potential against breast cancer cells. Compounds 7b, 7c and 7d did not exert significant toxicity on most of the tested cells, with the exception of 7c on HeLa cells. Thus, among the compounds of the molecule library we found some with remarkable toxic performance as well as obvious cancer cell selectivity, supporting the results of the primary screen.

**Table 3:** IC_50_ (±SD) values of some selected estradiol-based A-ring-integrated benzoxazolones as well as of 2ME2 and cisplatin assessed on A549 lung adenocarcinoma, DU-145 prostate adenocarcinoma, HeLa cervical adenocarcinoma and MCF-7 breast adenocarcinoma cancer cell lines and on non-cancerous MRC-5 lung fibroblast cells. Cells were treated with the tested compounds for 72 h, and then the viability of the cells was measured by MTT-assay

Compound	IC_50_ (μM) ± SD				
	A549	DU-145	HeLa	MCF-7	MRC-5
6a^a^	27.68 ± 1.96	20.67 ± 4.38	0.09 ± 0.01	—	>50
6b	10.04 ± 1.13	12.65 ± 1.21	2.12 ± 0.07	20.47 ± 1.81	>50
6c	2.89 ± 0.14	5.29 ± 0.53	2.00 ± 0.13	3.51 ± 0.46	>50
6d	12.36 ± 1.99	14.66 ± 1.23	3.91 ± 0.23	46.21 ± 16.24	>50
7a^a^	22.91 ± 1.43	>50	5.69 ± 0.46	—	>50
7b	>50	>50	34.57 ± 3.49	>50	>50
7c	>50	>50	4.96 ± 0.69	>50	>50
7d	>50	>50	>50	>50	>50
2ME2^a^	1.63 ± 0.31	0.71 ± 0.07	11.2 ± 3.33	1.11 ± 0.24	19.39 ± 3.12
Cisplatin^a^	5.39 ± 0.84	0.29 ± 0.02	1.42 ± 0.08	2.11 ± 0.15	7.41 ± 1.37

a Transferred data.^30^

**Fig. 3:**
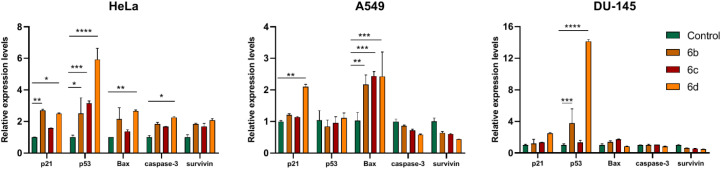
The relative mRNA levels of pro-apoptotic markers p21, p53, Bax, caspase-3, and survivin in HeLa, A549 and DU-145 cells treated with various *N*-substituted benzoxazolones. Two-way analysis of variance (ANOVA) followed by Dunnett's multiple comparisons tests, *, *P* < 0.03 **, *P* < 0.002 ***, *P* < 0.0002 ****, *P* < 0.0001.

In the first screens, we identified potent and cancer-selective compounds in the current molecule library, therefore, as a subsequent step, we examined whether these *N*-substituted benzoxazolones trigger apoptosis in cancer cells. In order to study this molecular event at its earliest point we determined the expression of several apoptotic marker genes (p21, p53, Bax, caspase-3, and survivin) by RT-qPCR. We selected the tumour suppressor and transcription factor protein p53 to verify apoptosis induction, as it is known for activating the expression of multiple target genes and plays critical roles in regulating cell cycle, apoptosis, and genomic stability. Also, the cyclin-dependent kinase inhibitor p21 functions as a regulator of cell cycle progression, DNA repair and apoptosis, as it interacts with proliferating cell nuclear antigen (PCNA), and can block the PCNA-dependent S-phase DNA synthesis. The expression of p21 is tightly regulated by p53 in response to a variety of stress stimuli. The Bcl-2 family member Bax is a pro-apoptotic factor involved in the formation of mitochondrial apoptosis-induced channel, and cytochrome c release to the cytosol. The expression of Bax is also controlled by p53. Caspase-3 is a cysteine–aspartic acid protease that plays a central role in the execution-phase of cell apoptosis. Finally, the anti-apoptotic inhibitor of apoptosis protein (IAP) family member survivin was also selected as it can physically interact with several caspases and inhibit their catalytic activity.^39,40^ For this examination, HeLa, A549 and DU-145 cells were treated with molecules 6b, 6c, or 6d in different concentrations for 24 hours. Each compound was applied on each cell line in a concentration corresponding to its IC_30_ value, which causes a 30% reduction in cell viability compared to the viability of untreated control cells. This IC_30_ concentration was selected for the subsequent experiments, since it is high enough to induce measurable loss of viability in the culture; however, it is low enough to keep a larger portion of the cells viable, and suitable for RNA extraction. Following treatments, total cellular RNA was extracted, reverse transcribed, and the relative expression levels of p21, p53, Bax, caspase-3, and survivin were determined (Fig. 3). We found that the mRNA expression of several apoptosis-related genes was altered when cancer cells were exposed to the *N*-substituted benzoxazolones. In HeLa cells, apart from that of survivin, the mRNA levels of all other apoptotic marker genes were elevated upon either 6b, 6c or 6d treatments, with p53 showing the highest induction in its expression. Bax expression in A549 cells, while p53 levels in DU-145 cells highlighted that these compounds triggered apoptotic cell death. Based on the RT-qPCR data analysis, the most potent apoptosis activating molecule resulted to be 6d. Although we could verify apoptotic gene expression changes induced by the selected compounds, we cannot exclude that other cell death mechanisms might also contribute significantly to the cytotoxic performance of the estradiol-based molecules in cancer cells. As sterane-based molecules have a very complex mechanism of action in humans, the identification of the exact biological target is beyond the scope of this study.

## Conclusions

A number of novel *N*-substituted benzoxazolone – estradiol chimeras with a common benzene ring were produced in good yields by *N*-alkylation or *N*-arylation of their unsubstituted precursors. According to computational predictions, only the *N*-alkylated regioisomeric pairs met all the filters of drug-likeness, although longer branched side chain is already unfavourable in terms of water solubility. The lack of substitution, or substitution with a smaller methyl group, increases the possibility that the molecule is a good substrate for P-gp, which may reduce its bioavailability. The biological effects of the obtained compounds were screened *in vitro* on cancer cell lines of various origins as well as on non-cancerous fibroblasts. These results suggested a structure–activity correlation favouring the heteroring fusion at 2,3 position and *N*-alkylation for anticancer purposes. Among the steroidal *N*-alkylated 2,3-fused oxazolones, 6c exhibited the lowest IC_50_ values on all of the cancerous cells, representing clearly the most efficient molecule from the library. To validate the apoptosis-inducing potential of the selected compounds an RT-qPCR-based approach was employed. This showed elevated Bax and p53 expression in cancer cells following treatments highlighting that apoptotic cell death was triggered by these compounds. The most potent apoptosis activating molecule resulted to be 6d. Based on the promising bioactivity of compounds 6c and 6d and their compliance with the drug-like criteria based on *in silico* studies, these two derivatives are considered to be suitable candidates for further investigation needed to gain a deeper understanding of their mechanism of action.

## Experimental

### Materials and methods

Reagents and solvents were purchased from commercial suppliers (Sigma-Aldrich and Alfa Aesar) and used without further purification. Melting points (Mps) were determined on an SRS Optimelt digital apparatus and are uncorrected. The transformations were monitored by TLC (0.25 mm thick Kieselgel-G plates (Si 254 F, Merck)). The compound spots were visualized by spraying with 5% phosphomolybdic acid in 50% aqueous phosphoric acid, followed by heating. Purifications by flash column chromatography (CC) were carried out on silica gel 60 (40–63 μm, Merck). Elementary analysis data were obtained with a PerkinElmer CHN analyser model 2400. NMR spectra were recorded with a Bruker DRX 500 instrument at room temperature in DMSO-d6 using residual solvent signals as an internal reference. Chemical shifts are reported in ppm (*δ* scale), and coupling constants (*J*) are given in Hz. Multiplicities of the ^1^H signals are indicated as a singlet (s), a doublet (d), a doublet of doublets (dd), a doublet of triplets (dt), a triplet (t), a triplet of doublets (td), a quartet (q), heptet (hep) or a multiplet (m). ^13^C NMR spectra are ^1^H-decoupled and the APT pulse sequence was used for multiplicity editing. In this spin-echo type experiment, both protonated and unprotonated carbons can be detected (CH_3_ and CH carbons appear as positive signals, while CH_2_ and C carbons as negative signals). The purified derivatives were dissolved in high purity acetonitrile and introduced with an Agilent 1290 Infinity II liquid chromatography pump to an Agilent 6470 tandem mass spectrometer equipped an electrospray ionization source. Flow rate was 0.5 mL min^−1^, and contained 0.1% formic acid to facilitate ionization. The instrument operated in MS1 scan mode with 135 V fragmentor voltage, and the spectra were recorded from 300 to 500 *m*/*z*, which were corrected with the background.

### Synthetic procedures

#### Two-step reduction of 2- (2) or 4-nitroestrone (3)

Nitroestrone derivative 2 or 3 (2.0 g, 6.3 mmol) was suspended in MeOH/THF (1 : 1, 20 mL) and sodium borohydride (477 mg, 2 equiv.) was added in small portions. After 1 h of stirring at room temperature, the solution was neutralized with diluted HCl, and extracted with EtOAc (3 × 10 mL). The combined organic layer was dried over anhydrous Na_2_SO_4_ and evaporated *in vacuo*. The residue was suspended in aqueous EtOH and sodium dithionite (4.4 g, 4 equiv.) was added under reflux. After 1 h of heating, the reaction mixture was poured into water (200 mL), and the precipitate was filtered off, washed with water and dried to give compound 4 or 5, in 96% or 90%, respectively. The NMR data of the produced compounds corresponded to those previously reported.^30^

#### General procedure for the synthesis of *N*-alkylated steroidal benzoxazolones (6b–e and 7b–e)

To a solution of compound 6a or 7a (100 mg, 0.32 mmol) in DMF (2 mL), K_2_CO_3_ (88 mg, 0.64 mmol, 2 equiv.) and the corresponding alkyl halide (0.96 mmol, 3 equiv.) were added, and then the reaction mixture was heated at 50 °C for 2 h (6b–e, 7b, 7c), 8 h (7d) or 20 h (7e). The reaction mixture was added dropwise to saturated NaHCO_3_ solution and extracted with EtOAc (3 × 10 mL). The combined organic layer was washed with water (10 mL) and NaCl solution (10 mL), dried over MgSO_4_. The crude product was purified by flash chromatography (10 → 40% EtOAc/hexane, gradient elution).

##### 17β-Hydroxy-3′-methyloxazolo[4′,5′:2,3]estra-1,3,5(10)-trien-2′-one (6b)

According to the general procedure, iodomethane (60 μl) was used. Yield: 90 mg (84%, white solid); Mp: 174–176 °C; anal. calcd for C_20_H_25_NO_3_ (327.42) C 73.37; H 7.70; N 4.28. Found C 73.45; H 7.77; N 4.31. ^1^H NMR (500 MHz, DMSO-*d*_6_): *δ* 0.68 (s, 3H, 18-CH_3_), 1.08–1.48 (m, 7H), 1.54–1.64 (m, 1H), 1.76–1.84 (m, 1H), 1.84–1.90 (m, 1H), 1.87–1.95 (m, 1H), 2.15–2.24 (m, 1H), 2.35–2.43 (m, 1H), 2.79–2.89 (m, 2H), 3.31 (s, 3H, *N*-CH_3_), 3.54 (td, *J* = 8.5, 4.8 Hz, 1H, 17-αH), 4.49 (d, *J* = 4.8 Hz, 1H, 17-OH), 6.99 (s, 1H, 4-H), 7.14 (s, 1H, 1-H); ^13^C NMR (125 MHz, DMSO-*d*_6_): *δ* 11.2 (18-CH_3_), 22.7 (CH_2_), 26.3 (CH_2_), 26.7 (CH_2_), 27.9 (N-CH_3_), 29.1 (CH_2_), 29.9 (CH_2_), 36.5 (CH_2_), 38.3 (8-CH), 42.7 (13-C), 44.0 (9-CH), 49.6 (14-CH), 79.9 (17-CH), 105.9 (1-CH), 109.1 (4-CH), 129.6 (C), 130.4 (C), 135.9 (C), 140.2 (3-C), 154.2 (C=O); ESI-MS: *m*/*z* 328.0 [M + H]^+^.

##### 17β-Hydroxy-3′-ethyloxazolo[4′,5′:2,3]estra-1,3,5(10)-trien-2′-one (6c)

According to the general procedure, bromoethane (72 μl) was used. Yield: 94 mg (86%, white solid); Mp: 183–185 °C; anal. calcd for C_21_H_27_NO_3_ (341.45) C 73.87; H 7.97; N 4.10. Found C 73.76; H 8.07; N 4.14. ^1^H NMR (500 MHz, DMSO-*d*_6_): *δ* 0.68 (s, 3H, 18-CH_3_), 1.22 (t, *J* = 7.2 Hz, 3H, CH_3_ of *N*-ethyl), 1.07–1.47 (m, 7H), 1.54–1.64 (m, 1H), 1.76–1.84 (m, 1H), 1.84–1.95 (m, 2H), 2.15–2.24 (m, 1H), 2.36–2.44 (m, 1H), 2.78–2.85 (m, 2H), 3.53 (td, *J* = 8.5, 4.8 Hz, 1H, 17-αH), 3.83 (q, *J* = 7.2 Hz, 2H, CH_2_ of *N*-ethyl), 4.49 (d, *J* = 4.8 Hz, 1H, 17-OH), 7.00 (s, 1H, 4-H), 7.18 (s, 1H, 1-H); ^13^C NMR (125 MHz, DMSO-*d*_6_): *δ* 11.2 (18-CH_3_), 12.8 (CH_3_ of *N*-ethyl), 22.7 (CH_2_), 26.3 (CH_2_), 26.7 (CH_2_), 29.1 (CH_2_), 29.9 (CH_2_), 36.4 (CH_2_), 36.5 (CH_2_), 38.3 (8-CH), 42.7 (13-C), 44.1 (9-CH), 49.6 (14-CH), 80.0 (17-CH), 105.9 (4-CH), 109.3 (1-CH), 128.6 (C), 130.4 (C), 135.9 (C), 140.2 (C), 153.7 (2′-C); ESI-MS: *m*/*z* 342.1 [M + H]^+^.

##### 17β-Hydroxy-3′-propyloxazolo[4′,5′:2,3]estra-1,3,5(10)-trien-2′-one (6d)

According to the general procedure, 1-bromopropane (86 μl) was used. Yield: 88 mg (78%, white solid); Mp: 176–178 °C; anal. calcd for C_22_H_29_NO_3_ (355,48) C 74.33; H 8.22; N 3.94. Found C 74.39; H 8.15; N 3.90. ^1^H NMR (500 MHz, DMSO-*d*_6_): *δ* 0.68 (s, 3H, 18-CH_3_), 0.88 (t, *J* = 7.4 Hz, 3H, CH_3_ of *N*-propyl), 1.06–1.46 (m, 7H), 1.54–1.63 (m, 1H), 1.68 (hep, *J* = 7.3 Hz, 2H, CH_2_ of *N*-propyl), 1.76–1.84 (m, 1H), 1.84–1.94 (m, 2H), 2.15–2.24 (m, 1H), 2.37–2.44 (m, 1H), 2.78–2.88 (m, 2H), 3.53 (td, *J* = 8.5, 4.8 Hz, 1H, 17-αH), 3.75 (t, *J* = 7.1 Hz, 2H, CH_2_ of N-propyl), 4.49 (d, *J* = 4.8 Hz, 1H, 17-OH), 6.99 (s, 1H, 4-H), 7.18 (s, 1H, 1-H); ^13^C NMR (125 MHz, DMSO-*d*_6_): *δ* 10.9 (CH_3_ of *N*-propyl), 11.2 (18-CH_3_), 20.6 (CH_2_ of *N*-propyl), 22.7 (CH_2_), 26.3 (CH_2_), 26.6 (CH_2_), 29.1 (CH_2_), 29.9 (CH_2_), 36.5 (CH_2_), 38.3 (8-CH), 42.7 (13-C), 42.9 (CH_2_ of *N*-propyl), 44.1 (9-CH), 49.6 (14-CH), 80.0 (17-CH), 106.0 (1-CH), 109.3 (4-CH), 129.0 (C), 130.4 (C), 135.9 (C), 140.1 (3-C), 154.1 (C=O); ESI-MS: *m*/*z* 356.2 [M + H]^+^.

##### 17β-Hydroxy-3′-isopropyloxazolo[4′,5′:2,3]estra-1,3,5(10)-trien-2′-one (6e)

According to the general procedure, 2-iodopropane (96 μl) was used. Yield: 94 mg (83%, white solid); Mp: > 100 °C (decomp.); anal. calcd for C_22_H_29_NO_3_ (355.48) C 74.33; H 8.22; N 3.94. Found C 74.21; H 8.29; N 4.01. ^1^H NMR (500 MHz, DMSO-*d*_6_): *δ* 0.68 (s, 3H, 18-CH_3_), 1.08–1.46 (m, 7H), 1.43 (d, *J* = 2.7 Hz, 3H, one CH_3_ of the *N*-isopropyl), 1.44 (d, *J* = 2.7 Hz, 3H, the other CH_3_ of the *N*-isopropyl), 1.54–1.64 (m, 1H), 1.76–1.84 (m, 1H), 1.84–1.94 (m, 2H), 2.15–2.24 (m, 1H), 2.36–2.44 (m, 1H), 2.77–2.84 (m, 2H), 3.53 (td, *J* = 8.5, 4.8 Hz, 1H, 17-αH), 4.48 (hep, *J* = 7.1 Hz, 1H, CH of the *N*-isopropyl), 4.49 (d, *J* = 4.9 Hz, 1H, 17-OH), 6.99 (s, 1H, 4-H), 7.19 (s, 1H, 1-H); ^13^C NMR (125 MHz, DMSO-*d*_6_): *δ* 11.2 (18-CH_3_), 19.3 (one CH_3_ of the *N*-isopropyl), 19.4 (the other CH_3_ of the *N*-isopropyl), 22.7 (CH_2_), 26.2 (CH_2_), 26.6 (CH_2_), 29.1 (CH_2_), 29.9 (CH_2_), 36.5 (CH_2_), 38.3 (8-CH), 42.7 (13-C), 44.1 (9-CH), 45.7 (CH of the *N*-isopropyl), 49.6 (14-CH), 80.0 (17-CH), 106.5 (4-CH), 109.3 (1-CH), 128.0 (C), 130.2 (C), 135.7 (C), 140.2 (C), 153.0 (2′-C); ESI-MS: *m*/*z* 356.2 [M + H]^+^.

##### 17β-Hydroxy-3′-methyloxazolo[4′,5′:3,4]estra-1,3,5(10)-trien-2′-one (7b)

According to the general procedure, iodomethane (60 μl) was used. Yield: 79 mg (75%, white solid); Mp: 169–170 °C; anal. calcd for C_20_H_25_NO_3_ (327.42) C 73.37; H 7.70; N 4.28. Found C 73.25; H 7.65; N 4.22. ^1^H NMR (500 MHz, DMSO-*d*_6_): *δ* 0.67 (s, 3H, 18-CH_3_), 1.06–1.44 (m, 7H), 1.56–1.66 (m, 1H), 1.82–1.95 (m, 3H), 2.16–2.24 (m, 1H), 2.27–2.35 (m, 1H), 3.03–3.14 (m, 1H), 3.25 (dd, *J* = 17.0, 5.7 Hz, 1H), 3.49–3.55 (m, 1H, 17-αH), 3.55 (s, 3H, *N*-CH_3_), 4.50 (d, *J* = 4.8 Hz, 1H, 17-OH), 7.07 (s, 2H, 1-H and 2-H); ^13^C NMR (125 MHz, DMSO-*d*_6_): *δ* 11.1 (18-CH_3_), 22.7 (CH_2_), 23.9 (CH_2_), 26.2 (CH_2_), 26.5 (CH_2_), 29.9 (CH_2_), 30.9 (N-CH_3_), 36.5 (CH_2_), 37.5 (8-CH), 42.6 (13-C), 44.0 (9-CH), 49.3 (14-CH), 79.9 (17-CH), 106.9 (2-CH), 119.4 (1-CH), 120.5 (5-C), 128.7 (4-C), 136.6 (10-C), 139.8 (3-C), 154.7 (C=O); ESI-MS: *m*/*z* 328.1 [M + H]^+^.

##### 17β-Hydroxy-3′-ethyloxazolo[4′,5′:3,4]estra-1,3,5(10)-trien-2′-one (7c)

According to the general procedure, bromomethane (72 μl) was used. Yield: 89 mg (81%, white solid); Mp: 167–169 °C; anal. calcd for C_21_H_27_NO_3_ (341.45) C 73.87; H 7.97; N 4.10. Found C 73.95; H 7.87; N 4.07. ^1^H NMR (500 MHz, DMSO-*d*_6_): *δ* 0.67 (s, 3H, 18-CH_3_), 1.25 (t, *J* = 7.0 Hz, 3H, CH_3_ of *N*-ethyl), 1.06–1.45 (m, 7H), 1.56–1.66 (m, 1H), 1.82–1.95 (m, 3H), 2.16–2.25 (m, 1H), 2.26–2.34 (m, 1H), 2.97–3.08 (m, 1H), 3.07–3.15 (m, 1H), 3.53 (td, *J* = 8.5, 4.5 Hz, 1H, 17-αH), 3.98 (q, *J* = 7.1 Hz, 2H, CH_2_ of *N*-ethyl), 4.50 (d, *J* = 4.8 Hz, 1H, 17-OH), 7.09 (s, 2H, 1-H and 2-H); ^13^C NMR (125 MHz, DMSO-*d*_6_): *δ* 11.2 (18-CH_3_), 15.0 (CH_3_ of *N*-ethyl), 22.7 (CH_2_), 23.7 (CH_2_), 26.1 (CH_2_), 26.5 (CH_2_), 29.9 (CH_2_), 36.6 (CH_2_), 37.4 (8-CH), 38.5 (CH_2_ of *N*-ethyl), 42.6 (13-C), 44.1 (9-CH), 49.4 (14-CH), 79.9 (17-CH), 107.0 (2-CH), 119.4 (1-CH), 120.0 (5-C), 127.9 (4-C), 136.8 (10-C), 140.0 (3-C), 154.4 (C=O); ESI-MS: *m*/*z* 343.1 [M + H]^+^.

##### 17β-Hydroxy-3′-propyloxazolo[4′,5′:3,4]estra-1,3,5(10)-trien-2′-one (7d)

According to the general procedure, 1-bromopropane (86 μl) was used. Yield: 86 mg (76%, white solid); Mp: 169–171 °C; anal. calcd for C_22_H_29_NO_3_ (355.48) C 74.33; H 8.22; N 3.94. Found C 74.45; H 8.13; N 3.91. ^1^H NMR (500 MHz, DMSO-*d*_6_): *δ* 0.67 (s, 3H, 18-CH_3_), 0.90 (t, *J* = 7.4 Hz, 3H, CH_3_ of *N*-propyl), 1.07–1.44 (m, 7H), 1.55–1.74 (m, 3H), 1.82–1.95 (m, 3H), 2.16–2.25 (m, 1H), 2.25–2.34 (m, 1H), 2.94–3.04 (m, 1H), 3.04–3.13 (m, 1H), 3.53 (td, *J* = 8.5, 5.0 Hz, 1H, 17-αH), 3.89 (t, *J* = 7.6 Hz, 2H, CH_2_ of *N*-propyl), 4.49 (d, *J* = 4.8 Hz, 1H, 17-OH), 7.09 (s, 2H, 1-H and 2-H); ^13^C NMR (125 MHz, DMSO-*d*_6_): *δ* 10.7 (CH_3_ of *N*-propyl), 11.1 (18- CH_3_), 22.7 (CH_2_), 23.0 (CH_2_ of *N*-propyl), 23.8 (CH_2_), 26.2 (CH_2_), 26.4 (CH_2_), 29.9 (CH_2_), 36.6 (CH_2_), 37.4 (8-CH), 42.6 (13-C), 44.1 (9-CH), 44.9 (CH_2_ of *N*-propyl), 49.5 (14-CH), 79.9 (17-CH), 107.0 (2-CH), 119.5 (1-CH), 120.1 (5-C), 128.0 (4-C), 136.8 (10-C), 140.0 (3-C), 154.7 (C=O); ESI-MS: *m*/*z* 356.2 [M + H]^+^.

##### 17β-Hydroxy-3′-isopropyloxazolo[4′,5′:3,4]estra-1,3,5(10)-trien-2′-one (7e)

According to the general procedure, 2-iodopropane (96 μl) was used. Yield: 60 mg (53%, white solid); Mp: 207–209 °C; anal. calcd for C_22_H_29_NO_3_ (355.48) C 74.33; H 8.22; N 3.94. Found C 74.27; H 8.26; N 3.98. ^1^H NMR (500 MHz, DMSO-*d*_6_): *δ* 0.67 (s, 3H, 18-CH_3_), 1.07–1.44 (m, 7H), 1.46 (d, *J* = 6.7 Hz, 3H, one CH_3_ of *N*-isopropyl), 1.50 (d, *J* = 6.7 Hz, 3H, the other CH_3_ of *N*-isopropyl), 1.56–1.66 (m, 1H), 1.81–1.95 (m, 3H), 2.16–2.25 (m, 1H), 2.26–2.33 (m, 1H), 3.03–3.09 (m, 2H), 3.53 (td, *J* = 8.5, 4.6 Hz, 1H, 17-αH), 4.50 (d, *J* = 4.8 Hz, 1H, 17-OH), 4.82 (hept, *J* = 6.8 Hz, 1H, CH of *N*-isopropyl), 7.06 (d, *J* = 8.5 Hz, 1H, 2-H), 7.09 (d, *J* = 8.6 Hz, 1H, 1-H); ^13^C NMR (125 MHz, DMSO-*d*_6_): *δ* 11.1 (18-CH_3_), 19.4 (one CH_3_ of *N*-isopropyl), 19.9 (the other CH_3_ of *N*-isopropyl), 22.7 (CH_2_), 25.2 (CH_2_), 26.3 (CH_2_), 26.7 (CH_2_), 29.9 (CH_2_), 36.6 (CH_2_), 37.3 (8-CH), 42.6 (13-C), 44.3 (9-CH), 47.5 (CH of *N*-isopropyl), 49.3 (14-CH), 79.9 (17-CH), 106.9 (2-CH), 119.6 (1-CH), 120.2 (5-C), 128.4 (4-C), 136.7 (10-C), 140.0 (3-C), 152.9 (C=O); ESI-MS: *m*/*z* 356.1 [M + H]^+^.

#### General procedure for the preparation of N-(hetero)aryl benzoxazolones (8a–g) by Chan–Evans–Lam coupling

To a solution of compound 6a (100 mg, 0.32 mmol) in EtOH (2 mL) pyridine (52 μL, 2 equiv.), CuCl_2_ catalyst (8.6 mg, 20 mol%), and the corresponding (hetero)arylboronic acid (3 equiv.) were added. After stirring at room temperature for 24 h, the reaction mixture was poured into water (10 mL) and then extracted with EtOAc (3 × 10 mL). The combined organic phase was washed with HCl solution (10 mL, 1 M), water (10 mL), NaCl solution (10 mL), dried over MgSO_4_ and evaporated. The crude product was purified by flash chromatography (10 → 60% EtOAc/hexane, gradient elution).

##### 17β-Hydroxy-3′-phenyloxazolo[4′,5′:2,3]estra-1,3,5(10)-trien-2′-one (8a)

According to the general procedure, phenylboronic acid (116 mg) was used. Yield: 100 mg (81%, white solid); Mp: > 75 °C (decomp.); anal. calcd for C_25_H_27_NO_3_ (389.50) C 77.09; H 6.99; N 3.60. Found C 76.98; H 7.05; N 3.64. ^1^H NMR (500 MHz, DMSO-*d*_6_) *δ* 0.64 (s, 3H, 18-CH_3_), 1.05–1.43 (m, 7H), 1.54–1.64 (m, 1H), 1.77–1.93 (m, 3H), 2.09–2.21 (m, 2H), 2.82–2.89 (m, 2H), 3.51 (td, *J* = 8.5, 4.4 Hz, 1H, 17-αH), 4.48 (d, *J* = 4.7 Hz, 1H, 17-OH), 6.91 (s, 1H, 4-H), 7.13 (s, 1H, 1-H), 7.44–7.54 (m, 1H, 4′′-H), 7.60 (d, *J* = 5.8 Hz, 4H, 2′′-H, 3′′-H, 5′′-H and 6′′-H); ^13^C NMR (126 MHz, DMSO-*d*_6_): *δ* 11.1 (18-CH_3_), 22.7 (CH_2_), 26.2 (CH_2_), 26.6 (CH_2_), 29.1 (CH_2_), 29.9 (CH_2_), 36.4 (CH_2_), 38.1 (8-CH), 42.7 (13-C), 43.8 (9-CH), 49.5 (14-CH), 79.9 (17-CH), 105.7 (4-CH), 109.7 (1-CH), 125.3 (2′′-CH and 6′′-CH), 128.2 (4′′-CH), 128.9 (2-C), 129.7 (3′′-CH and 5′′-CH), 131.5 (5-C), 133.4 (10-C), 136.1 (1′′-C), 140.3 (3-C), 152.7 (2′-C); ESI-MS: *m*/*z* 390.0 [M + H]^+^.

##### 17β-Hydroxy-3′-(4′′-fluorophenyl)oxazolo[4′,5′:2,3]estra-1,3,5(10)-trien-2′-one (8b)

According to the general procedure, 4-fluorophenyl boronic acid (134 mg) was used. Yield: 104 mg (80%, white solid); Mp: > 85 °C (decomp.); anal. calcd for C_25_H_26_FNO_3_ (407.49) C 73.69; H 6.43; N 3.44. Found C 73.77; H 6.37; N 3.42. ^1^H NMR (500 MHz, DMSO-*d*_6_): *δ* 0.64 (s, 3H, 18-CH_3_), 1.06–1.43 (m, 7H), 1.54–1.64 (m, 1H), 1.77–1.85 (m, 2H), 1.82–1.93 (m, 1H), 2.12–2.21 (m, 2H), 2.82–2.89 (m, 2H), 3.51 (td, *J* = 8.5, 4.0 Hz, 1H, 17-αH), 4.48 (d, *J* = 4.7 Hz, 1H, 17-OH), 6.88 (s, 1H, 4-H), 7.13 (s, 1H, 1-H), 7.40–7.48 (m, 2H, 2′′-H and 6′′-H), 7.62–7.69 (m, 2H, 3′′-H and 5′′-H); ^13^C NMR (125 MHz, DMSO-*d*_6_): *δ* 11.1 (18-CH_3_), 22.7 (CH_2_), 26.2 (CH_2_), 26.6 (CH_2_), 29.1 (CH_2_), 29.9 (CH_2_), 36.4 (CH_2_), 38.2 (8-CH), 42.7 (13-C), 43.8 (9-CH), 49.5 (14-CH), 79.9 (17-CH), 105.6 (4-CH), 109.7 (1-CH), 116.7 (d, *J* = 23.0 Hz, 3′′-CH and 5′′-CH), 127.9 (d, *J* = 9.0 Hz, 2′′-CH and 6′′-CH), 129.6 (C), 129.6 (C), 131.5 (C), 136.1 (C), 140.3 (3-C), 152.7 (C=O), 161.2 (d, *J* = 245.5 Hz, 4′′-C); ESI-MS: *m*/*z* 408.0 [M + H]^+^.

##### 17β-Hydroxy-3′-(4′′-chlorophenyl)oxazolo[4′,5′:2,3]estra-1,3,5(10)-trien-2′-one (8c)

According to the general procedure, 4-chlorophenyl boronic acid (150 mg) was used. Yield: 100 mg (74%, white solid); Mp: > 115 °C (decomp.); anal. calcd for C_25_H_26_ClNO_3_ (423.94) C 70.83; H 6.18; N 3.30. Found C 70.96; H 6.09; N 3.32. ^1^H NMR (500 MHz, DMSO-*d*_6_): *δ* 0.65 (s, 3H, 18-CH_3_), 1.06–1.34 (m, 5H), 1.31–1.43 (m, 2H), 1.54–1.64 (m, 1H), 1.78–1.85 (m, 2H), 1.83–1.94 (m, 1H), 2.10–2.23 (m, 2H), 2.82–2.89 (m, 2H), 3.51 (td, *J* = 8.5, 4.2 Hz, 1H, 17-αH), 4.48 (d, *J* = 4.8 Hz, 1H, 17-OH), 6.94 (s, 1H, 4-H), 7.13 (s, 1H, 1-H), 7.61–7.69 (m, 4′H, 2′′-H, 3′′-H, 5′′-H and 6′′-H); ^13^C NMR (125 MHz, DMSO-*d*_6_): *δ* 11.2 (18-CH_3_), 22.7 (CH_2_), 26.2 (CH_2_), 26.6 (CH_2_), 29.1 (CH_2_), 29.9 (CH_2_), 36.4 (CH_2_), 38.2 (8-CH), 42.7 (13-C), 43.8 (9-CH), 49.5 (14-CH), 79.9 (17-CH), 105.8 (4-CH), 109.8 (1-CH), 127.2 (2′′-CH and 6′′-CH), 128.7 (C), 129.7 (3′′-CH and 5′′-CH), 131.7 (C), 132.3 (C), 132.4 (C), 136.2 (C), 140.3 (3-C), 152.5 (C=O); ESI-MS: *m*/*z* 424.0 [M + H]^+^.

##### 17β-Hydroxy-3′-(4′′-bromophenyl)oxazolo[4′,5′:2,3]estra-1,3,5(10)-trien-2′-one (8d)

According to the general procedure, 4-bromophenyl boronic acid (192 mg) was used. Yield: 132 mg (88%, white solid); Mp: > 170 °C (decomp.); anal. calcd for C_25_H_26_BrNO_3_ (468.39) C 64.11; H 5.60; N 2.99. Found C 64.14; H 5.52; N 3.03. ^1^H NMR (500 MHz, DMSO-*d*_6_): *δ* 0.65 (s, 3H, 18-CH_3_), 1.07–1.43 (m, 7H), 1.54–1.63 (m, 1H), 1.78–1.85 (m, 2H), 1.83–1.94 (m, 1H), 2.10–2.23 (m, 2H), 2.82–2.89 (m, 2H), 3.51 (td, *J* = 8.5, 4.6 Hz, 1H, 17-αH), 4.48 (d, *J* = 4.8 Hz, 1H, 17-OH), 6.95 (s, 1H, 4-H), 7.13 (s, 1H, 1-H), 7.55–7.61 (m, 2H, 2′′-H and 6′′-H), 7.76–7.83 (m, 2H, 3′′-H and 5′′-H); ^13^C NMR (125 MHz, DMSO-*d*_6_): *δ* 11.2 (18-CH_3_), 22.7 (CH_2_), 26.2 (CH_2_), 26.6 (CH_2_), 29.1 (CH_2_), 29.9 (CH_2_), 36.4 (CH_2_), 38.2 (8-CH), 42.7 (13-C), 43.9 (9-CH), 49.5 (14-CH), 79.9 (17-CH), 105.8 (4-CH), 109.8 (1-CH), 120.8 (C), 127.5 (2′′-CH and 6′′-CH), 128.6 (C), 131.7 (C), 132.7 (3′′-CH and 5′′-CH), 132.8 (C), 136.2 (C), 140.3 (3-C), 152.5 (C=O). ESI-MS: *m*/*z* 467.8 [M + H]^+^.

##### 17β-Hydroxy-3′-(4′′-cyanophenyl)oxazolo[4′,5′:2,3]estra-1,3,5(10)-trien-2′-one (8e)

According to the general procedure, 4-cyanophenyl boronic acid (140 mg) was used. Yield: 88 mg (67%, white solid); Mp: 213–214 °C; anal. calcd for C_26_H_26_N_2_O_3_ (414.51) C 75.34; H 6.32; N 6.76. Found C 75.42; H 6.39; N 6.78. ^1^H NMR (500 MHz, DMSO-*d*_6_): *δ* 0.65 (s, 3H, 18-CH_3_), 1.08–1.45 (m, 7H), 1.54–1.64 (m, 1H), 1.77–1.99 (m, 3H), 2.14–2.26 (m, 2H), 2.80–2.89 (m, 2H), 3.52 (td, *J* = 8.5, 4.8 Hz, 1H, 17-αH), 4.48 (d, *J* = 4.8 Hz, 1H, 17-OH), 7.07 (s, 1H, 4-H), 7.16 (s, 1H, 1-H), 7.82–7.89 (m, 2H, 2′′-H and 6′′-H), 8.05–8.11 (m, 2H, 3′′-H and 5′′-H); ^13^C NMR (125 MHz, DMSO-*d*_6_): *δ* 11.2 (18-CH_3_), 22.7 (CH_2_), 26.1 (CH_2_), 26.6 (CH_2_), 29.1 (CH_2_), 29.9 (CH_2_), 36.4 (CH_2_), 38.2 (8-CH), 42.7 (13-C), 43.9 (9-CH), 49.5 (14-CH), 79.9 (17-CH), 106.2 (4-CH), 109.9 (1-CH), 110.2 (4′′-C), 118.3 (4′′-CN) 125.6 (2′′-CH and 6′′-CH), 127.9 (C), 132.1 (C), 133.9 (3′′-CH and 5′′-CH), 136.3 (C), 137.7 (C), 140.4 (3-C), 152.2 (2′-C); ESI-MS: *m*/*z* 415.0 [M + H]^+^.

##### 17β-Hydroxy-3′-(4′′-ethylphenyl)oxazolo[4′,5′:2,3]estra-1,3,5(10)-trien-2′-one (8f)

According to the general procedure, 4-ethylphenyl boronic acid (144 mg) was used. Yield: 112 mg (84%, white solid); Mp: > 80 °C (decomp.); anal. calcd for C_27_H_31_NO_3_ (417.55) C 77.67; H 7.48; N 3.35. Found C 77.79; H 7.42; N 3.31. ^1^H NMR (500 MHz, DMSO-*d*_6_): *δ* 0.64 (s, 3H, 18-CH_3_), 1.06–1.43 (m, 7H), 1.24 (t, *J* = 7.5 Hz, 3H, ethyl-CH_3_), 1.53–1.63 (m, 1H), 1.77–1.86 (m, 2H), 1.83–1.93 (m, 1H), 2.10–2.21 (m, 2H), 2.70 (q, *J* = 7.6 Hz, 2H, ethyl-CH_2_), 2.82–2.88 (m, 2H), 3.47–3.54 (m, 1H, 17-αH), 4.48 (d, *J* = 4.8 Hz, 1H, 17-OH), 6.89 (s, 1H, 4-H), 7.12 (s, 1H, 1-H), 7.43 (d, *J* = 8.3 Hz, 2H, 3′′-H and 5′′-H), 7.49 (d, *J* = 8.4 Hz, 2H, 2′′-H and 6′′-H); ^13^C NMR (125 MHz, DMSO-*d*_6_): *δ* 11.1 (18-CH_3_), 15.4 (ethyl-CH_3_), 22.7 (CH_2_), 26.2 (CH_2_), 26.6 (CH_2_), 27.8 (ethyl-CH_2_), 29.1 (CH_2_), 29.9 (CH_2_), 36.4 (CH_2_), 38.2 (8-CH), 42.7 (13-C), 43.8 (9-CH), 49.5 (14-CH), 79.9 (17-CH), 105.7 (4-CH), 109.7 (1-CH), 125.3 (2′′-CH and 6′′-CH), 129.0 (3′′-CH and 5′′-CH), 129.0 (C), 130.9 (C), 131.4 (C), 136.1 (C), 140.3 (C), 143.9 (C), 152.8 (C=O); ESI-MS: *m*/*z* 418.1 [M + H]^+^.

##### 17β-Hydroxy-3′-(3′′-pyridyl)oxazolo[4′,5′:2,3]estra-1,3,5(10)-trien-2′-one (8g)

According to the general procedure, pyridine-3-boronic acid (118 mg) was used. Yield: 86 mg (69%, white solid); Mp: > 100 °C (decomp.); anal. calcd for C_24_H_26_N_2_O_3_ (390.48) C 73.82; H 6.71; N 7.17. Found C 73.96; H 6.80; N 7.15. ^1^H NMR (500 MHz, DMSO-*d*_6_): *δ* 0.65 (s, 3H, 18-CH_3_), 1.06–1.43 (m, 7H), 1.54–1.64 (m, 1H), 1.78–1.94 (m, 3H), 2.10–2.23 (m, 2H), 2.83–2.89 (m, 2H), 3.51 (t, *J* = 8.5 Hz, 1H, 17-αH), 4.48 (s, 1H, 17-OH), 6.97 (s, 1H, 4-H), 7.16 (s, 1H, 1-H), 7.65 (dd, *J* = 8.2, 4.8 Hz, 1H, 5′′-H), 8.08 (dt, *J* = 8.2, 2.0 Hz, 1H, 4′′-H), 8.68 (dd, *J* = 4.8, 1.5 Hz, 1H, 6′′-H), 8.85 (d, *J* = 2.5 Hz, 1H, 2′′-H); ^13^C NMR (125 MHz, DMSO-*d*_6_): *δ* 11.2 (18-CH_3_), 22.7 (CH_2_), 26.1 (CH_2_), 26.6 (CH_2_), 29.1 (CH_2_), 29.9 (CH_2_), 36.4 (CH_2_), 38.2 (8-CH), 42.7 (13-C), 43.9 (9-CH), 49.5 (14-CH), 79.9 (17-CH), 105.8 (4-CH), 109.8 (1-CH), 124.5 (5′′-CH), 128.6 (C), 130.5 (C), 131.9 (C), 133.2 (4′′-CH), 136.3 (C), 140.4 (C), 146.5 (2′′-CH), 149.0 (6′′-CH), 152.7 (C=O); ESI-MS: *m*/*z* 391.0 [M + H]^+^.

#### *In silico* ADME and drug-likeness prediction

The *in silico* ADME screening and drug-likeness evaluation were performed using the SwissADME web tool (ESI) developed by Swiss Institute of Bioinformatics^32^ freely available online at http://www.swissadme.ch. After entering the structures of the cyclic carbamates using the ChemAxon's Marvin JS drawing tool, simple physicochemical properties, such as molecular weight (MW), counts of atom types, molecular refractivity (MR), and topological polar surface area (TPSA) can be computed. SwissADME gives access to five freely available predictive models of lipophilicity (*i.e.* iLOGP, XLOGP3, WLOGP, MLOGP, and SILICOS-IT) from which a consensus log *P*_o/w_ is determined.^32^ Three different methods (ESOL, Ali, SILICOS-IT) to predict water solubility (log *S*) are also included. Unique to SwissADME is the bioavailability radar that provides a graphical snapshot of the drug-likeness parameters of an orally available bioactive drug. The drug-likeness graph is presented as a hexagon (ESI) with each of the vertices representing a parameter that define a bioavailable drug. The pink area within the hexagon represents the optimal range for each property (lipophilicity (LIPO): XLOGP3 between −0.7 and +5.0, size: MW (SIZE) between 150 and 500 g mol^−1^, polarity (POLAR): TPSA between 20 and 130 Å^2^, solubility (INSOLU): log *S* not higher than 6, saturation (INSATU): fraction of carbons in the sp^3^ hybridization not less than 0.25, and flexibility (FLEX): no more than 9 rotatable bonds). Drug-likeness evaluation is implemented by computational filters developed by leading pharmaceutical companies and cheminfomaticians that include Lipinski, Ghose, Veber, Egan, and Muegge rules of 5 (RO5). The Abbott Bioavailability scores are computed to predict the probability of a compound to have at least 10% oral bioavailability by relying on total charge, TPSA, and violation of the Lipinski's filter. SwissADME includes “BOILEDegg evaluation” that predict gastrointestinal absorption (HIA) and efflux/retention by P-glycoprotein (P-gp). “BOILEDegg” is a graphical evaluation of HIA as a function of the position of the small molecule in the WLOGP-versus-TPSA plot. Additionally, blood–brain barrier (BBB) penetration and Cytochrome P450 (CYP) enzyme substrate-inhibition prediction can be made. The white part of the “BOILEDegg” represents the probability that a molecule will be passively absorbed through the GI tract, and the yellow region (yolk) represents the high probability of penetration into the brain. Yolk and white areas are not mutually exclusive. In addition, the points are coloured in blue if predicted as actively effluxed by P-gp (PGP+) and in red if predicted as non-substrate of P-gp (PGP−). Importantly, false positive results commonly seen in biochemical assays of small molecules are predicted with a fair degree of certainty, since the virtual tool has a complimentary substructure filter for removal of pan assay interference compounds (PAINS) from screening libraries and for their exclusion in bioassays. Brenk alerts, on the other hand, depict fragments of compounds that are likely to be metabolically unstable.^32^

### Pharmacology

#### Cell cultures

The A549 human adenocarcinomic alveolar basal epithelial cells, DU-145 prostate adenocarcinoma, HeLa cervical carcinoma, MCF-7 breast adenocarcinoma and the human non-cancerous fibroblast cell line MRC-5 were obtained from American Type Culture Collection (ATCC, Manassas, VA, USA). Cancerous cell lines were cultured in RPMI-1640 medium (Sigma-Aldrich, Saint Louis, MO, USA), the non-cancerous MRC-5 cells were maintained in EMEM medium (Sigma-Aldrich, Saint Louis, MO, USA). Media were complemented with 10% fetal bovine serum, 2 mM glutamine and 1% penicillin–streptomycin. Cells were cultured at 37 °C under 5% CO_2_ and at 95% humidity under standard conditions.

#### Cell viability tests

The test compounds were dissolved in DMSO (Molar Chemicals, Halásztelek, Hungary) to yield stock solutions with a final concentration of 10 mM. Compound 6e was not soluble in DMSO, thus it was excluded from the pharmacological screening. To determine the toxicity of each compound, 3000 cells per well were seeded into 96-well plates and left to grow for 24 hours. Then, the cells were treated with the test molecules individually in 2.5 μM as well as in 5 μM concentrations for 72 h. At the end of the incubation time, cells were washed with PBS and culture media containing 0.5 mg per mL 3-(4,5-dimethylthiazolyl-2)-2,5-diphenyltetrazolium bromide (MTT) reagent (SERVA, GmbH, Heidelberg, Germany) was administered to the cells. After 1-hour incubation at 37 °C, the resulting formazan crystals were dissolved in DMSO and the absorbance of the solutions were obtained at 570 nm using a Synergy HTX plate reader (BioTech-Hungary, Budapest, Hungary). Compounds showing advantageous anti-cancer potentials were selected and their IC_50_ values were determined on various cell lines and compared with those of 2ME2 and cisplatin. For this, the seeded cells were exposed to the selected test molecules or to 2ME2 or cisplatin in various concentrations and the experiments were performed as described above. Dose–response curves were fitted on the viability data, and IC_50_ values were calculated accordingly. The viability of the control cells (obtaining only the solvent DMSO) was considered 100%. MTT assays were repeated at least three times using four independent biological replicates. Graphical representation of the viability data as heat map or dose–response curves, as well as the calculation of IC_50_ values were carried out by GraphPad Prism 8 software.

#### Reverse transcription and real-time qPCR

To verify apoptosis induction in cancer cells by the test compounds, A549, HeLa and DU-145 cells were seeded at 6 × 10^5^ cells per well density in 60 mm dishes and on the next day were treated with either compound 6b (in 6 μM to treat A549, 7.6 μM for DU-145 and in 1.3 μM for HeLa), 6c (in 1.8 μM for A549, 3.2 μM for DU-145 and 1.2 μM for HeLa) or 6d (in 7.4 μM for A549, 8.8 μM for DU-145 and 2.4 μM for HeLa) for 24 h. Then total RNA was extracted from the cells using RNeasy® Mini Kit (QIAGEN, Hilden, Germany) according to the manufacturer's recommendation. 2 μg RNA was reverse transcribed (RevertAid First Strand cDNA Synthesis kit, Thermo Scientific, Walthan, MA, USA) in 20 μL total volume, then 2 μL cDNA was used as input upon PCR reactions for determining the expression of various apoptosis marker genes (primer sequences and primer concentrations are shown in Table 4). qPCR experiments were performed on PicoReal™ Real-time PCR (Thermo Scientific, Walthan, MA, USA) using Maxima SYBRGreen qPCR Master Mix (Thermo Scientific, Walthan, MA, USA). Relative transcript levels were determined by the ΔΔCt method using GAPDH as reference gene. Experiments were repeated three times with three biological replicates.

**Table 4:** List, sequence and concentrations of primers used for qPCR

Gene	Primer sequences (forward, reverse)	Final conc. [nM]
*p21*	5′-CAGCAGAGGAAGACCATGTG-3′	200
	5′-GGCGTTTGGAGTGGTAGAAA-3′	
*p53*	5′-CCCTTCCCAGAAAACCTACC-3′	200
	5′-CTCCGTCATGTGCTGTGACT-3′	
*Bax*	5′-TGCTTCAGGGTTTCATCCAG-3′	200
	5′-GGCGGCAATCATCCTCTG-3′	
*Casp-3*	5′-ACATGGCGTGTCATAAAATACC-3′	200
	5′-CACAAAGCGACTGGATGAAC-3′	
*survivin*	5′-AGAACTGGCCCTTCTTGGAGG-3′	200
	5′-CTTTTTATCTTCCTCTATGGGGTC-3′	
*GAPDH*	5′-TGCACCACCAACTGCTTAGC-3′	200
	5′-GGCATGGACTGTGGTCATGAG-3′	

#### Statistical analysis

For the statistical evaluation GraphPad prism software was employed. Data are presented as the mean ± standard deviation (SD), and were analyzed by two-way analysis of variance (ANOVA) followed by Dunnett's multiple comparisons tests. Statistical significance was set at *p* < 0.05. The representative significance values are *, *P* < 0.03 **, *P* < 0.002 ***, *P* < 0.0002 ****, *P* < 0.0001.

## Author contributions

Conceptualization, resources, supervision and writing – original draft, É. F. and M. K.; investigation, writing – review and editing, F. K. and I. H.; investigation and methodology, H. Á. and M. K. All authors have read and agreed to the published version of the manuscript.

## Conflicts of interest

The authors declare no conflict of interest.

## Supplementary Material

RA-015-D5RA01977J-s001

## Data Availability

The data supporting this article have been included as part of the ESI.†
