# Supplementary material for: Aromatic scaffold-integrated hybrids of estradiol and benzoxazol-2-ones: synthesis and in vitro anticancer activity of N-substituted regioisomeric pairs
Source: RSC Adv. 2025 Jul 10;15(29):23954–65. doi: 10.1039/d5ra01977j (PMC12242395; doi:10.1039/d5ra01977j)
Supplement: RA-015-D5RA01977J-s001 [file RA-015-D5RA01977J-s001.pdf]

Supplementary Information (SI) for RSC Advances.
This journal is © The Royal Society of Chemistry 2025

Electronic Supplementary Information for

# Aromatic scaffold-integrated hybrids of estradiol and benzoxazol-2-ones: synthesis and *in vitro* anticancer activity of *N*-substituted regioisomeric pairs

Ferenc Kovács[1], Ildikó Huliák[2], Hédi Árva[2], Marianna Kocsis,[2] Mónika Kiricsi[2], Éva Frank[1,*]

[1]*Department of Molecular and Analytical Chemistry, University of Szeged, Dóm tér 7-8, H-6720 Szeged, Hungary*

[2]*Department of Biochemistry and Molecular Biology, Doctoral School of Biology, University of Szeged, Közép fasor 52., H-6726 Szeged, Hungary*

*Corresponding author: frank@chem.u-szeged.hu; Tel.: +36-62-544-275

## Table of contents

$^{1}$H- and $^{13}$C NMR spectra of the newly synthesized steroidal cyclic carbamates 2-16

Computed ADME parameters and drug-likeness of the synthesized compounds (**6a-e, 8a-g and 7a-e**) including ″BOILEDegg″ evaluation 17-33

**Table S1**. Selected physicochemical properties, lipophilicity (logP) and water solubility (logS) of the synthesized molecules computed by the SwissADME web tool 34

**Table S2**. Evaluation of drug-likeness and selected pharmacokinetic properties of the synthesized molecules using swissADME 35

**Table S3**. Values of primary growth inhibitory screen used for heat map construction 36

**Fig. S1**. Representative cell viability curves to determine growth inhibition and $IC_{50}$ values following treatments with the selected compounds on different cell lines 37

1H — DMSO

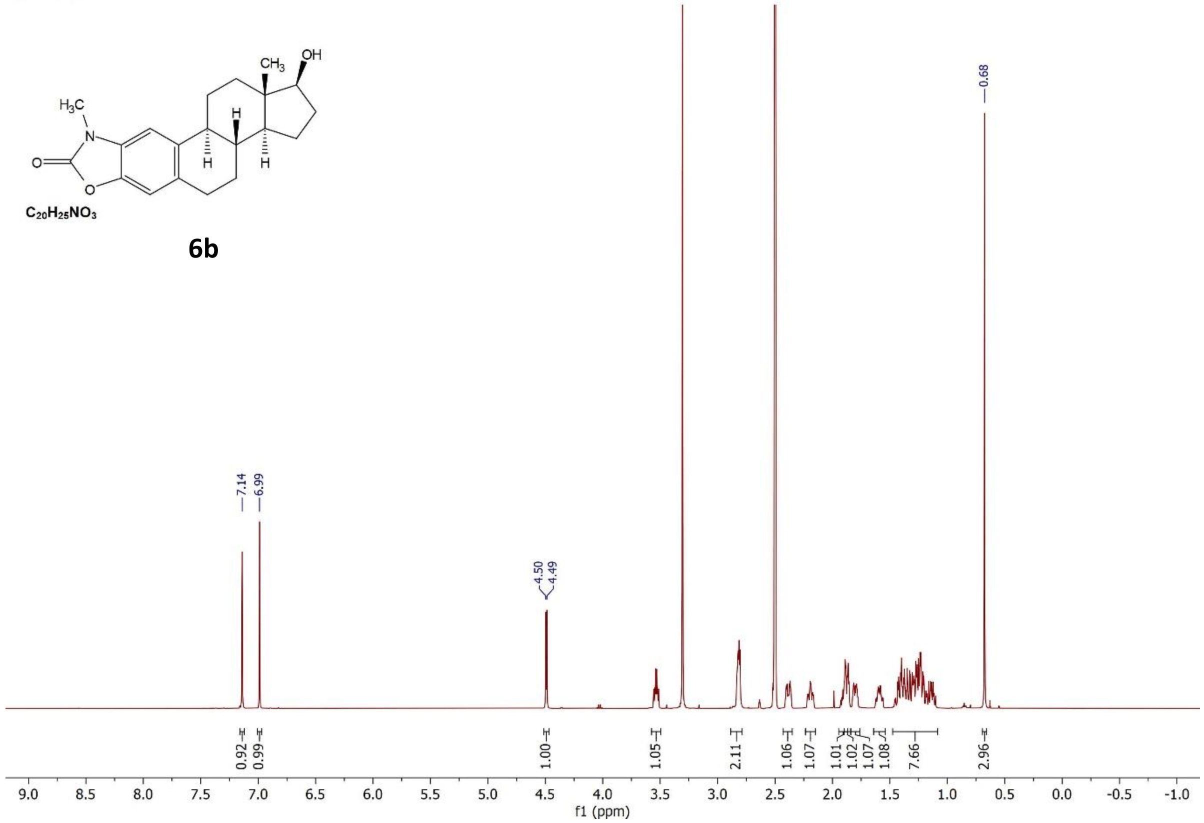


13C — DMSO

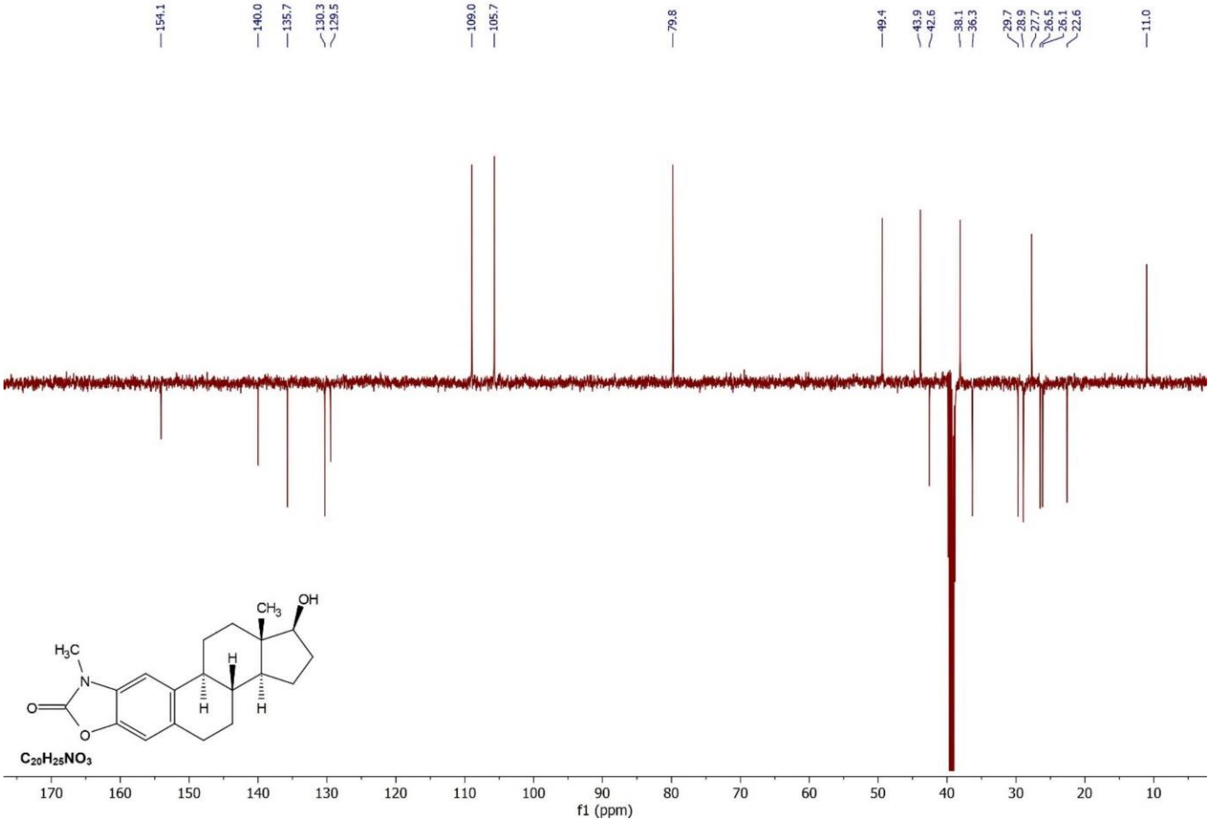

1H — DMSO

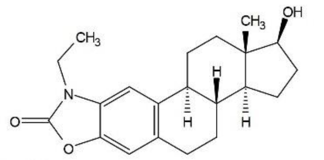


$C_{21}H_{27}NO_3$

**6c**

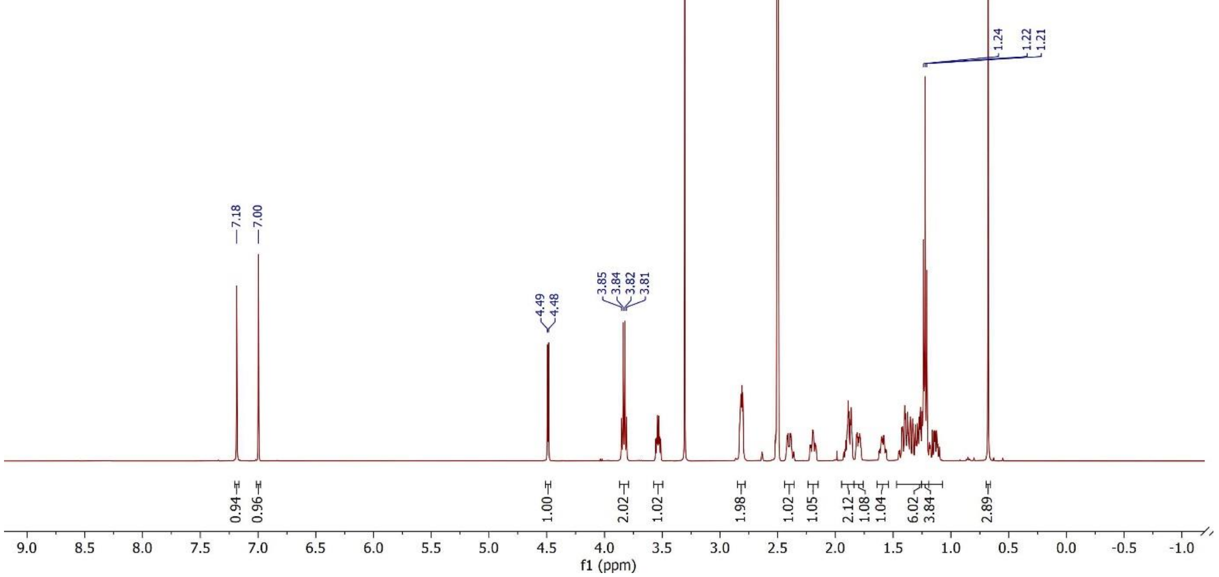


13C — DMSO

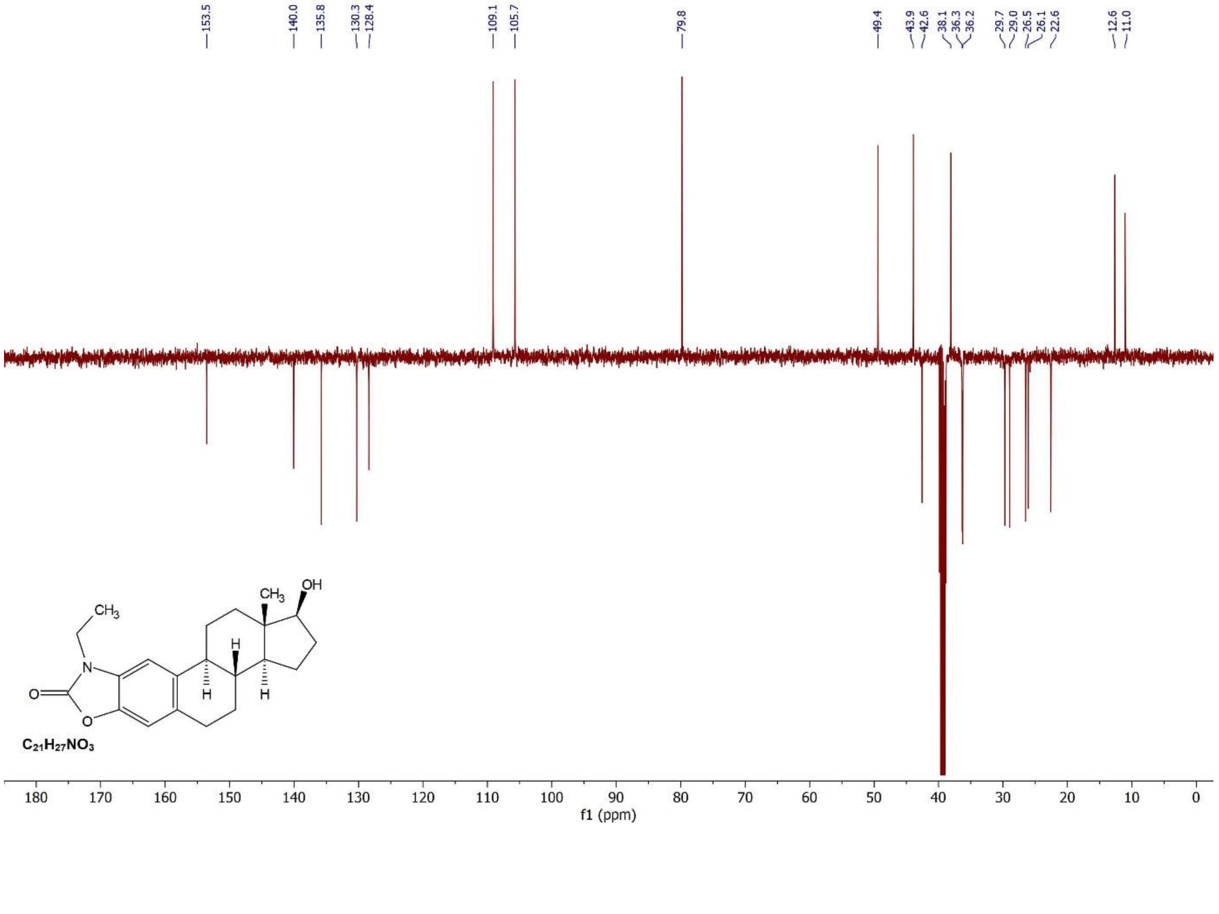

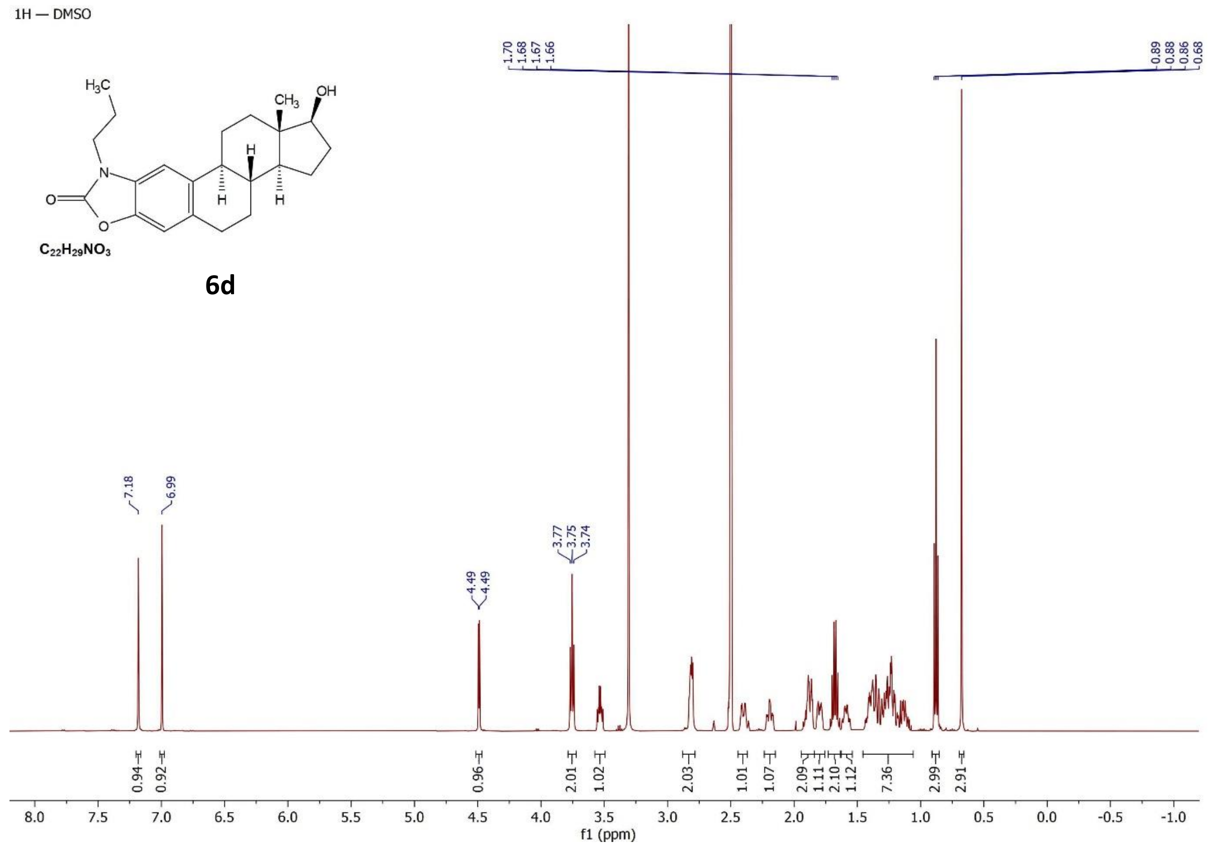
1H — DMSO
$H_3C$
$CH_3$
OH
N
O
H
$C_{22}H_{29}NO_3$
7.18
6.99
4.49
4.49
3.77
3.75
3.74
1.70
1.68
1.67
1.66
0.89
0.88
0.86
0.68
0.94
0.92
0.96
2.01
1.02
2.03
1.01
1.07
2.09
1.11
2.10
1.12
7.36
2.99
2.91
f1 (ppm)

**6d**

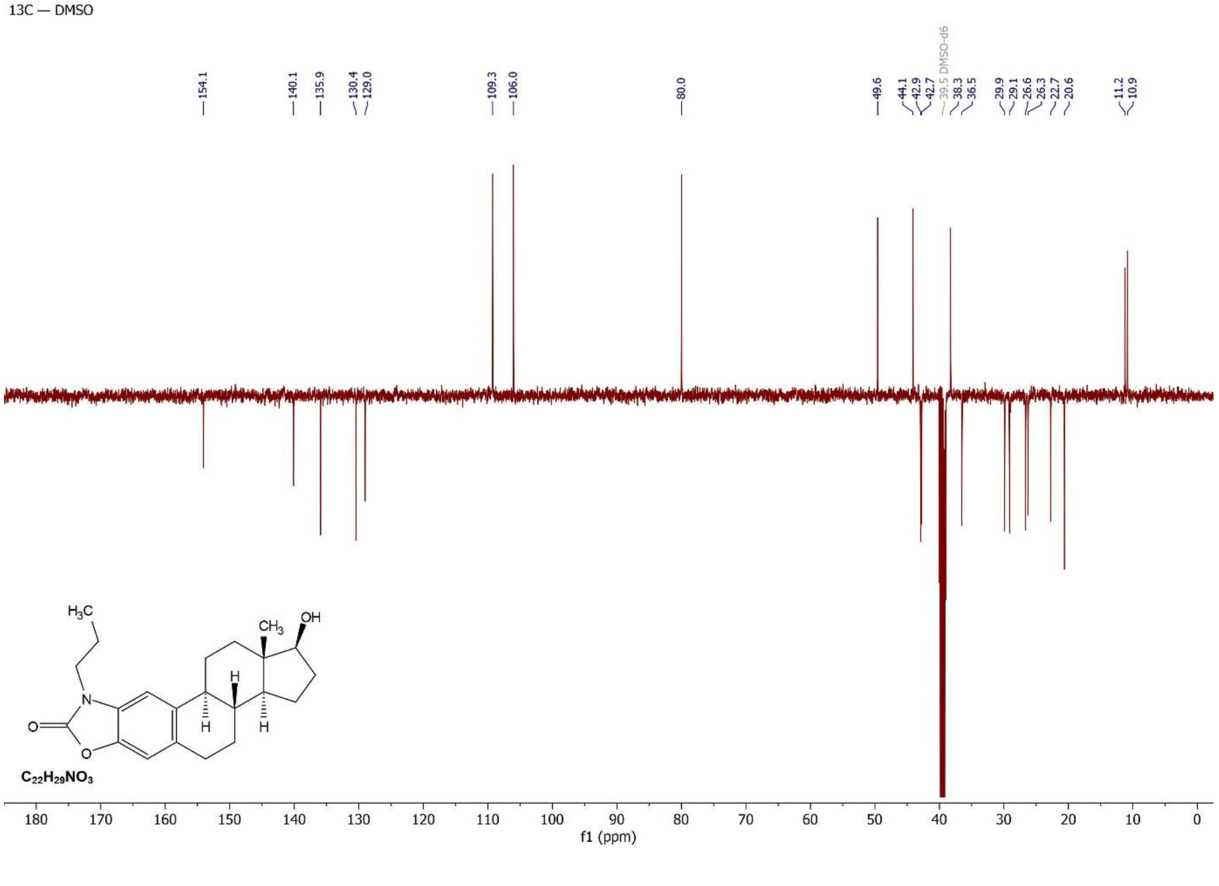
13C — DMSO
154.1
140.1
135.9
130.4
129.0
109.3
106.0
80.0
49.6
44.1
42.9
42.7
39.5 DMSO-d6
38.3
36.5
29.9
29.1
26.6
26.3
22.7
20.6
11.2
10.9
$H_3C$
$CH_3$
OH
N
O
H
$C_{22}H_{29}NO_3$
f1 (ppm)

1H — DMSO

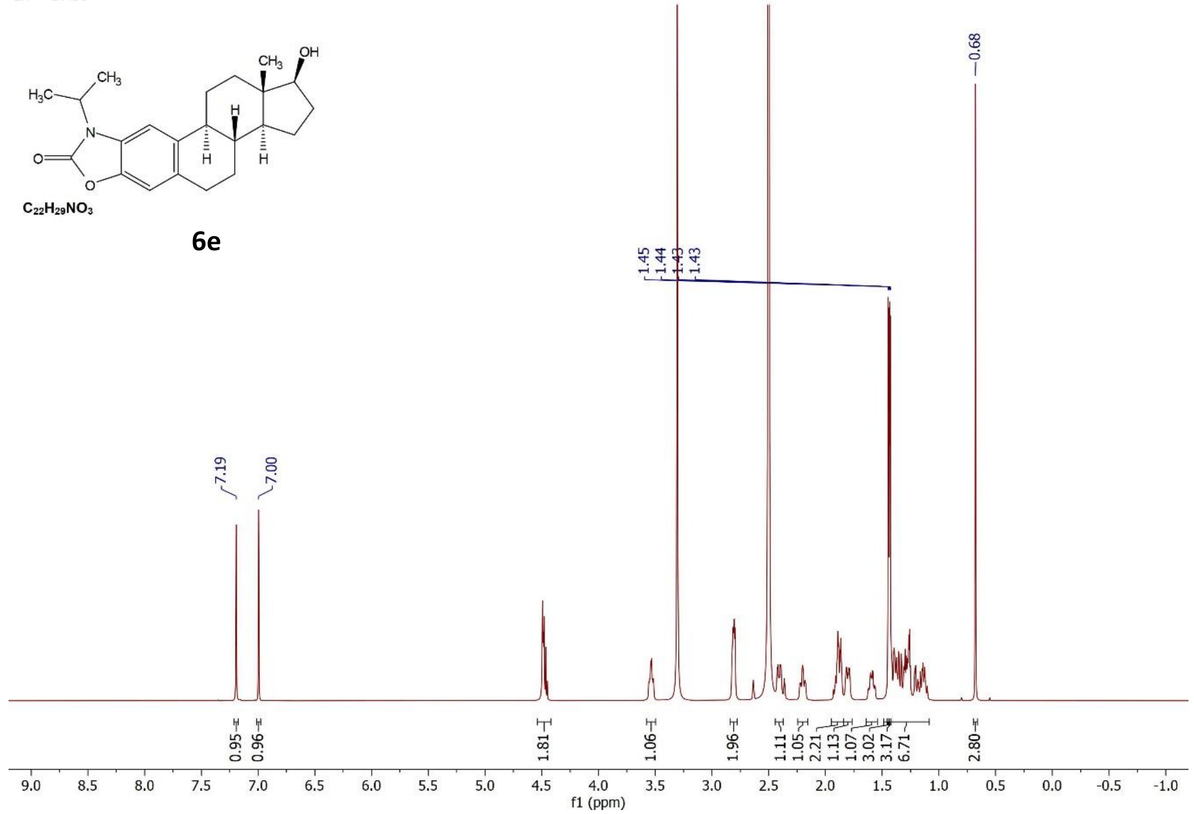


13C — DMSO

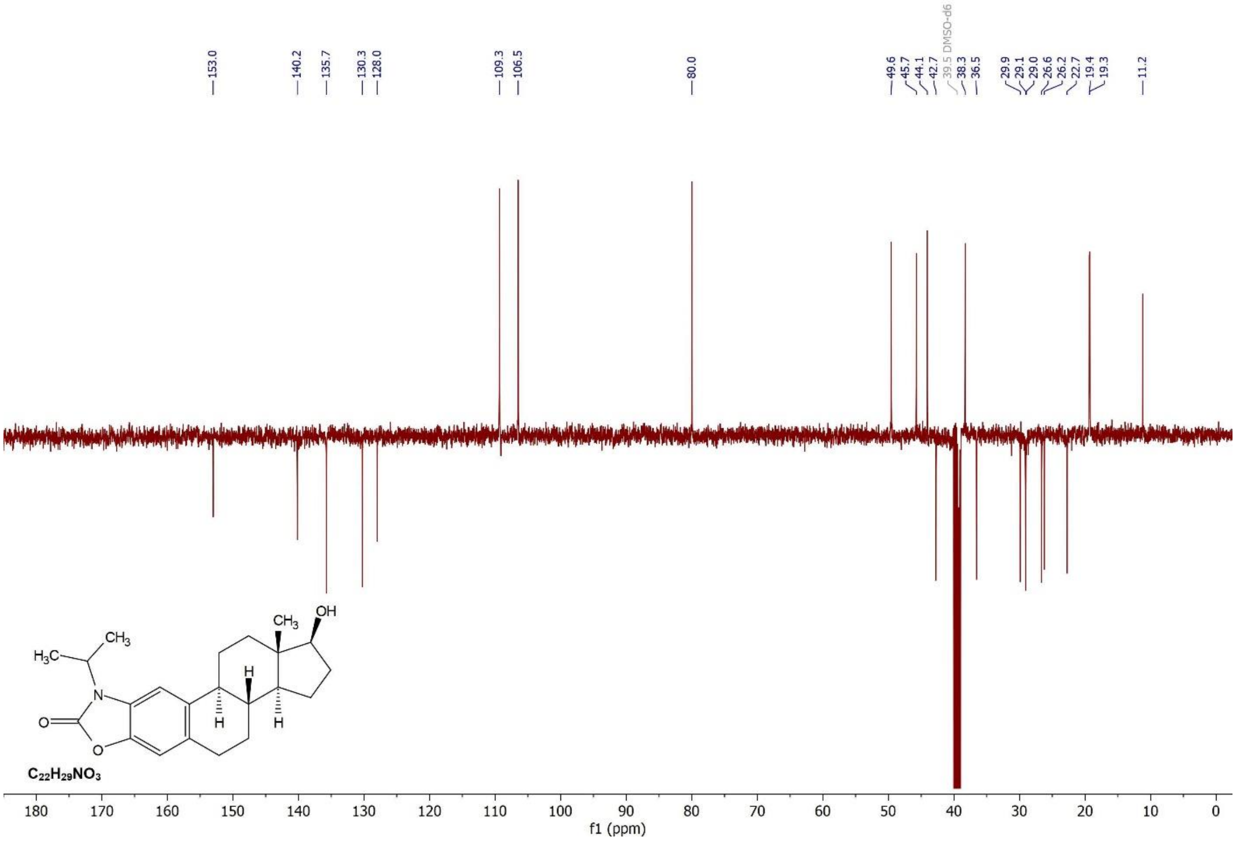

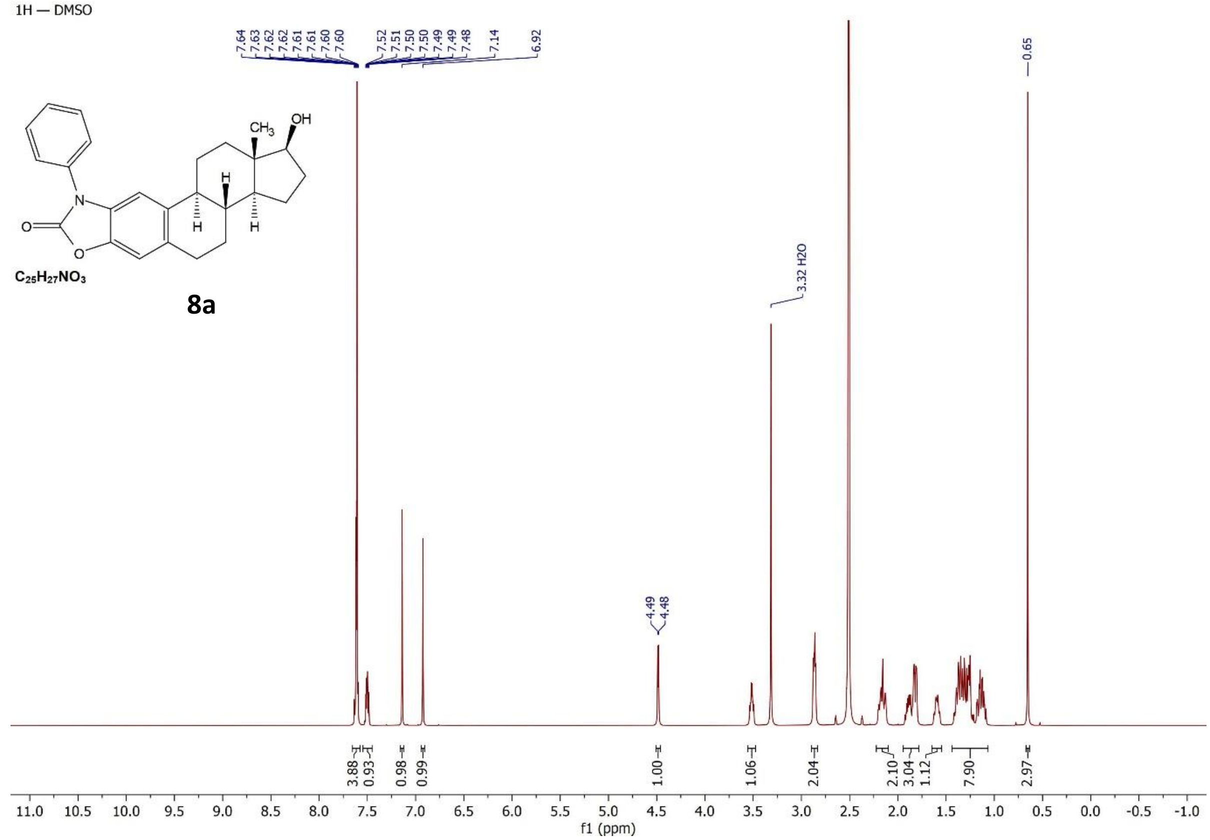
1H — DMSO
8a
$C_{25}H_{27}NO_3$
7.64 7.63 7.62 7.62 7.61 7.61 7.60 7.60 7.52 7.51 7.50 7.50 7.49 7.49 7.48 7.14 6.92
3.32 H2O
0.65
4.49 4.48
3.88 0.93 0.98 0.99 1.00 1.06 2.04 2.10 3.04 1.12 7.90 2.97
f1 (ppm)

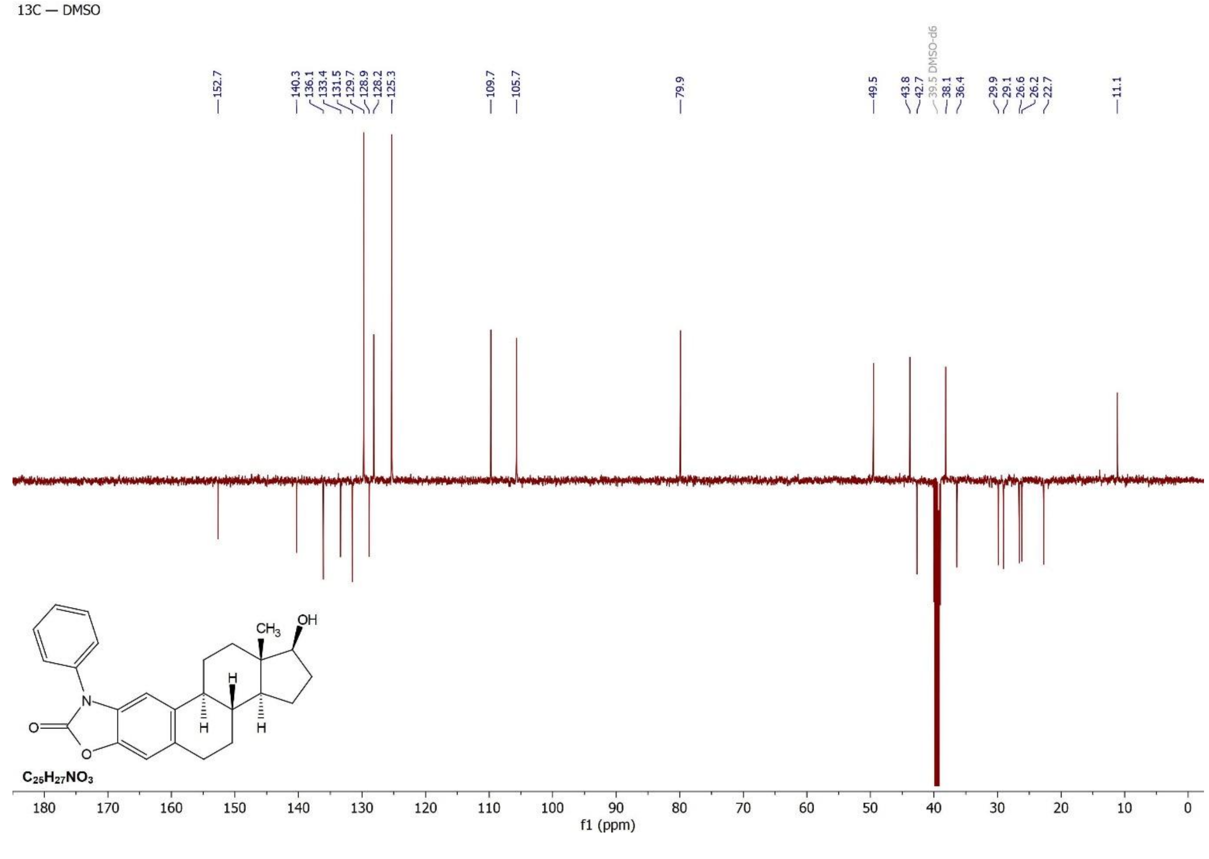
13C — DMSO
152.7 140.3 136.1 133.4 131.5 129.7 128.9 128.2 125.3 109.7 105.7 79.9 49.5 43.8 42.7 39.5 DMSO-d6 38.1 36.4 29.9 29.1 26.6 26.2 22.7 11.1
$C_{25}H_{27}NO_3$
f1 (ppm)

1H — DMSO

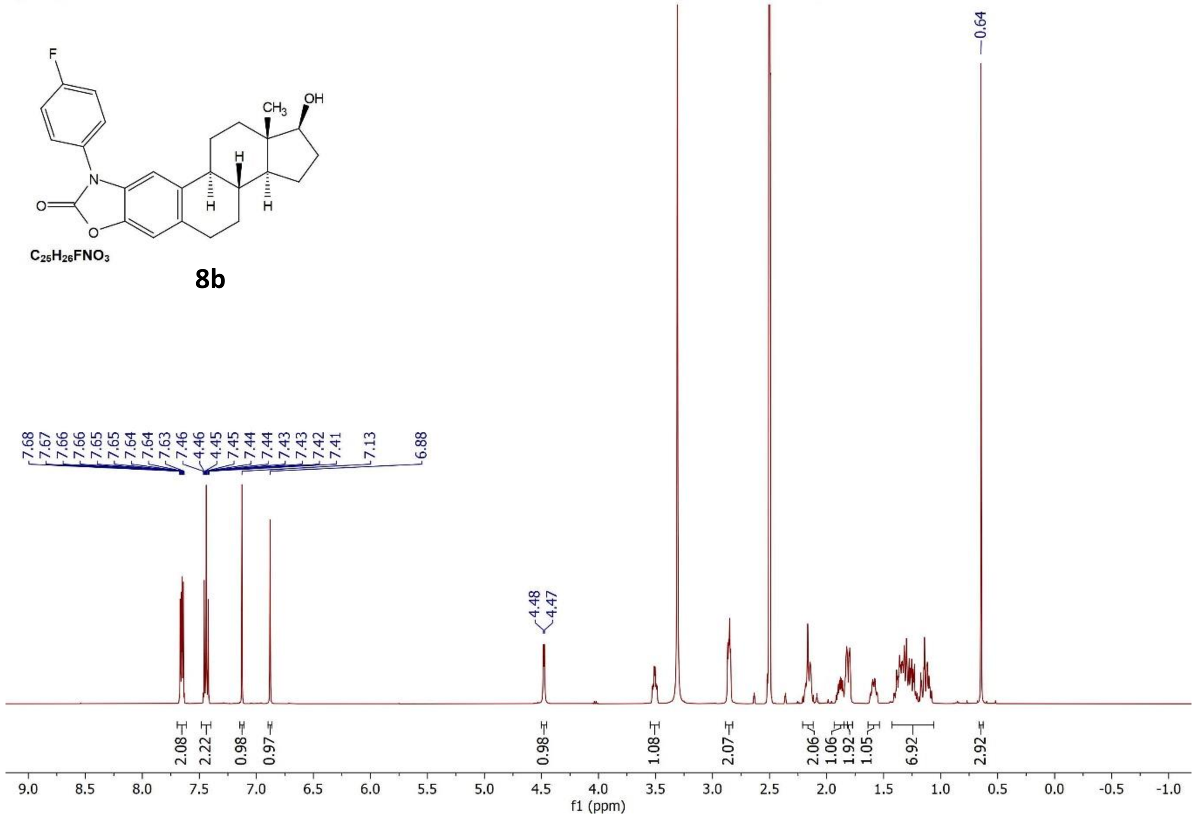


13C — DMSO

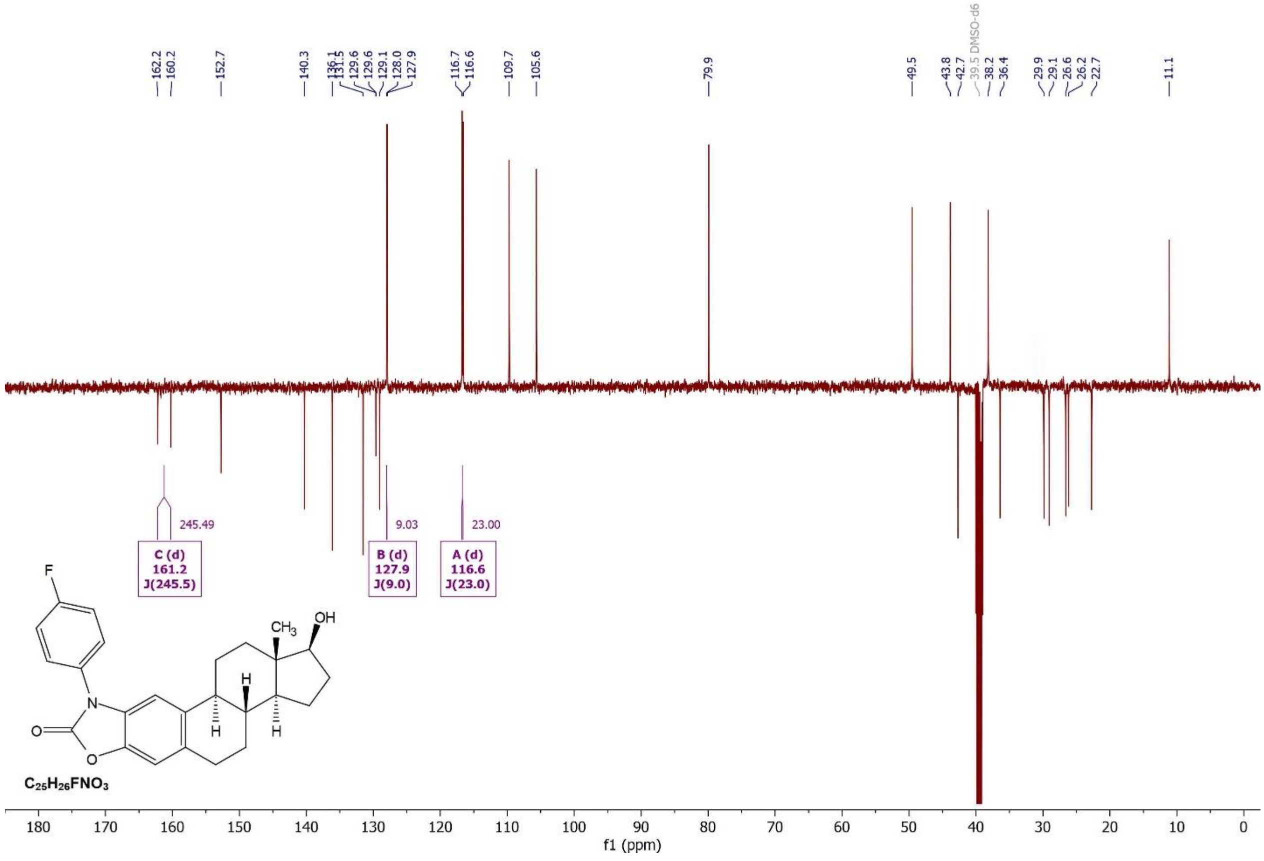

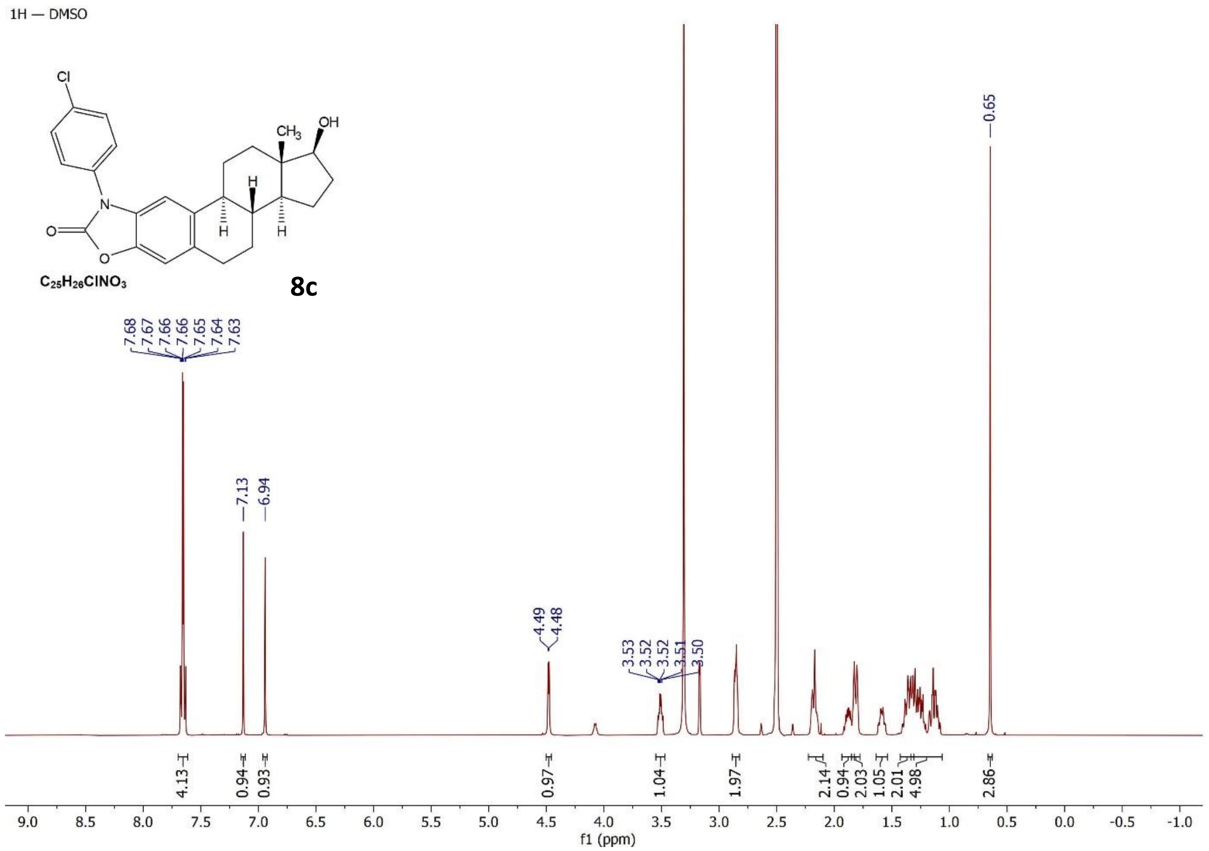
1H — DMSO
Cl
$CH_3$
OH
N
O
H
H
H
$C_{25}H_{26}ClNO_3$
8c
7.68
7.67
7.66
7.66
7.65
7.64
7.63
7.13
6.94
4.49
4.48
3.53
3.52
3.52
3.51
3.50
0.65
4.13
0.94
0.93
0.97
1.04
1.97
2.14
0.94
2.03
1.05
2.01
4.98
2.86
f1 (ppm)

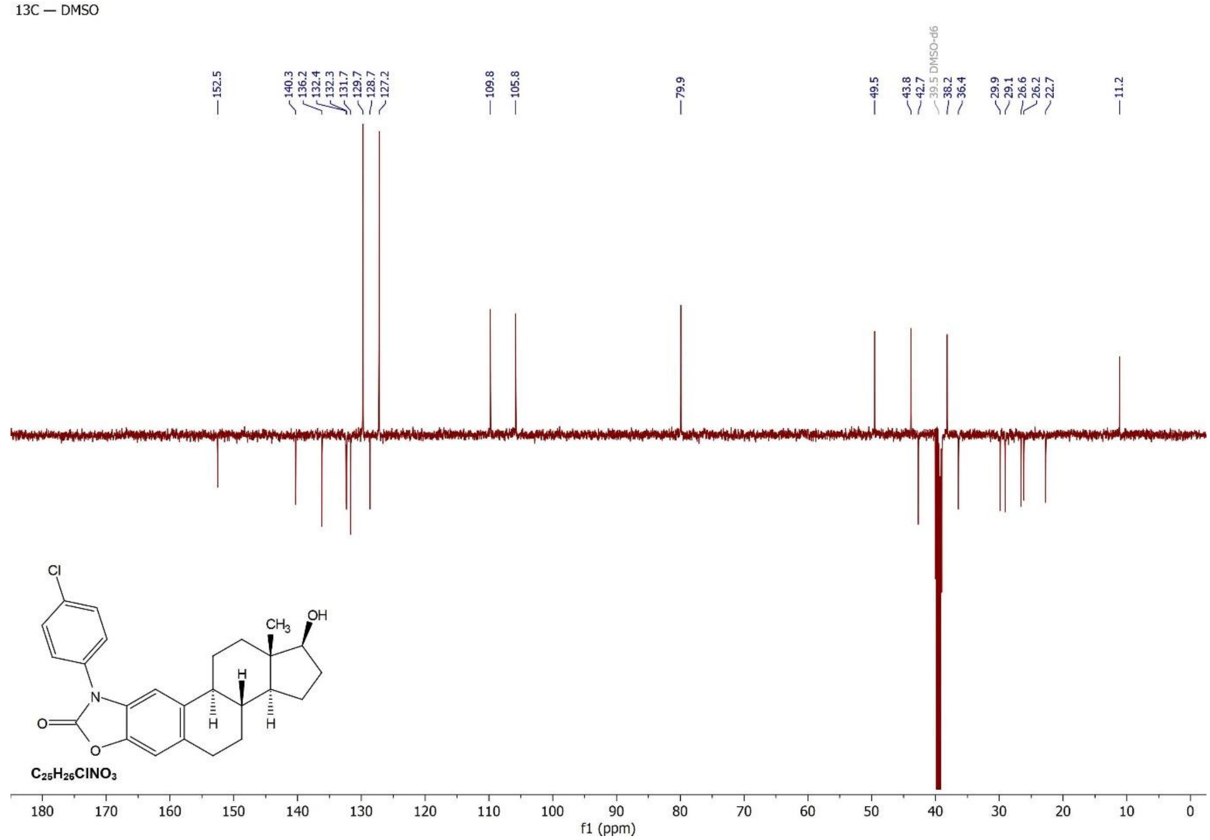
13C — DMSO
152.5
140.3
136.2
132.4
132.3
131.7
129.7
128.7
127.2
109.8
105.8
79.9
49.5
43.8
42.7
39.5 DMSO-d6
38.2
36.4
29.9
29.1
26.6
26.2
22.7
11.2
Cl
$CH_3$
OH
N
O
H
H
H
$C_{25}H_{26}ClNO_3$
f1 (ppm)

1H — DMSO

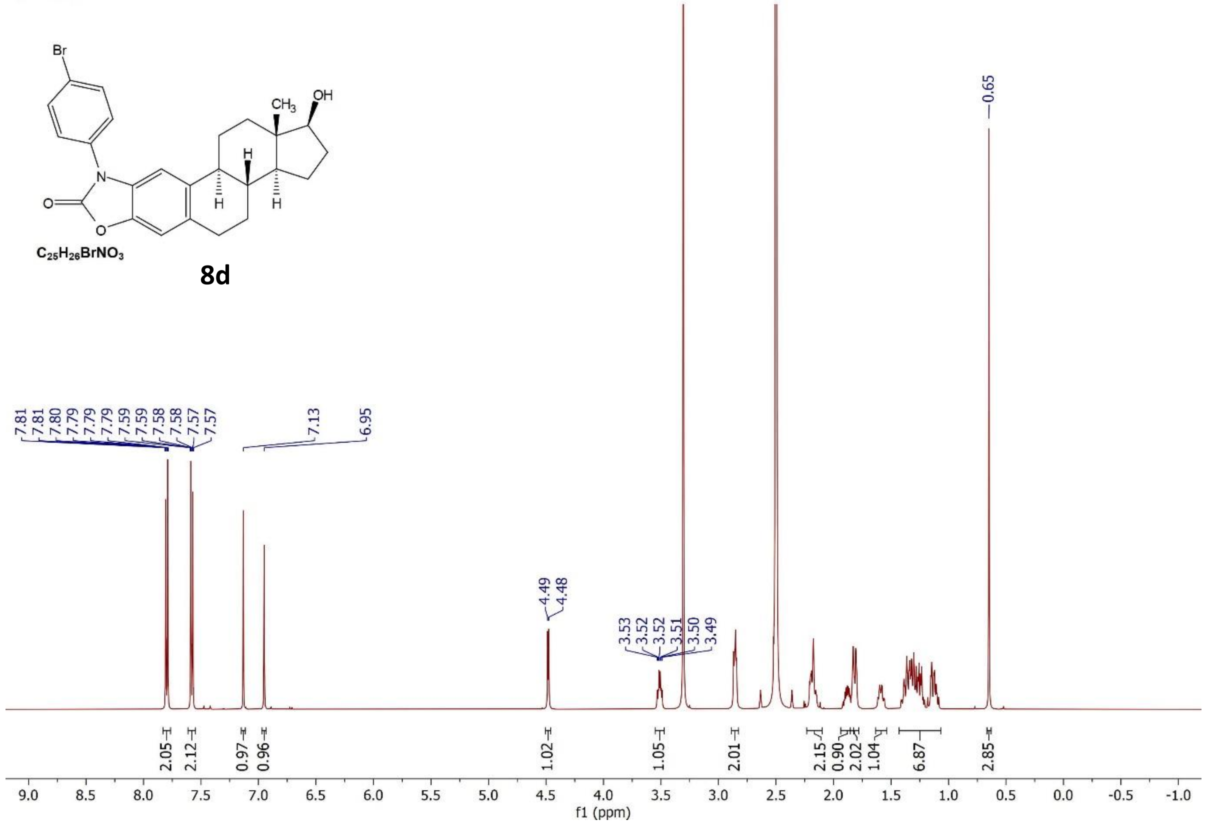


13C — DMSO

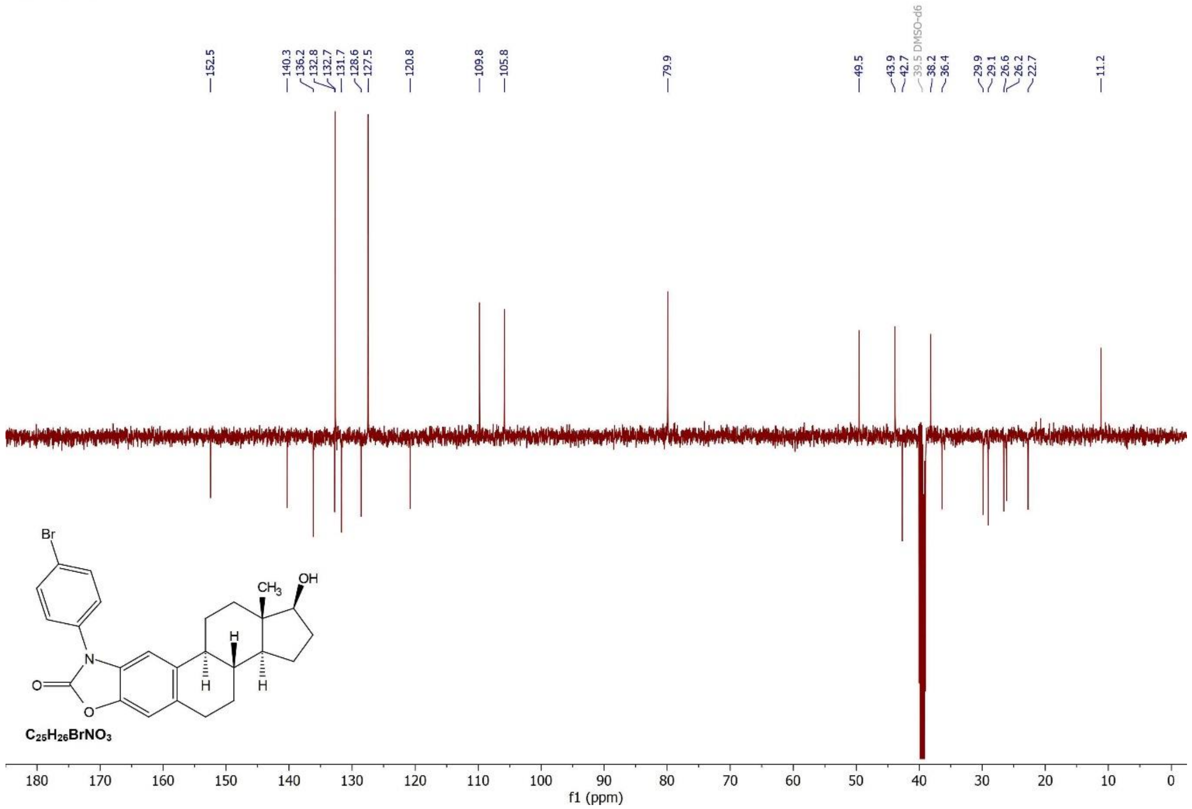

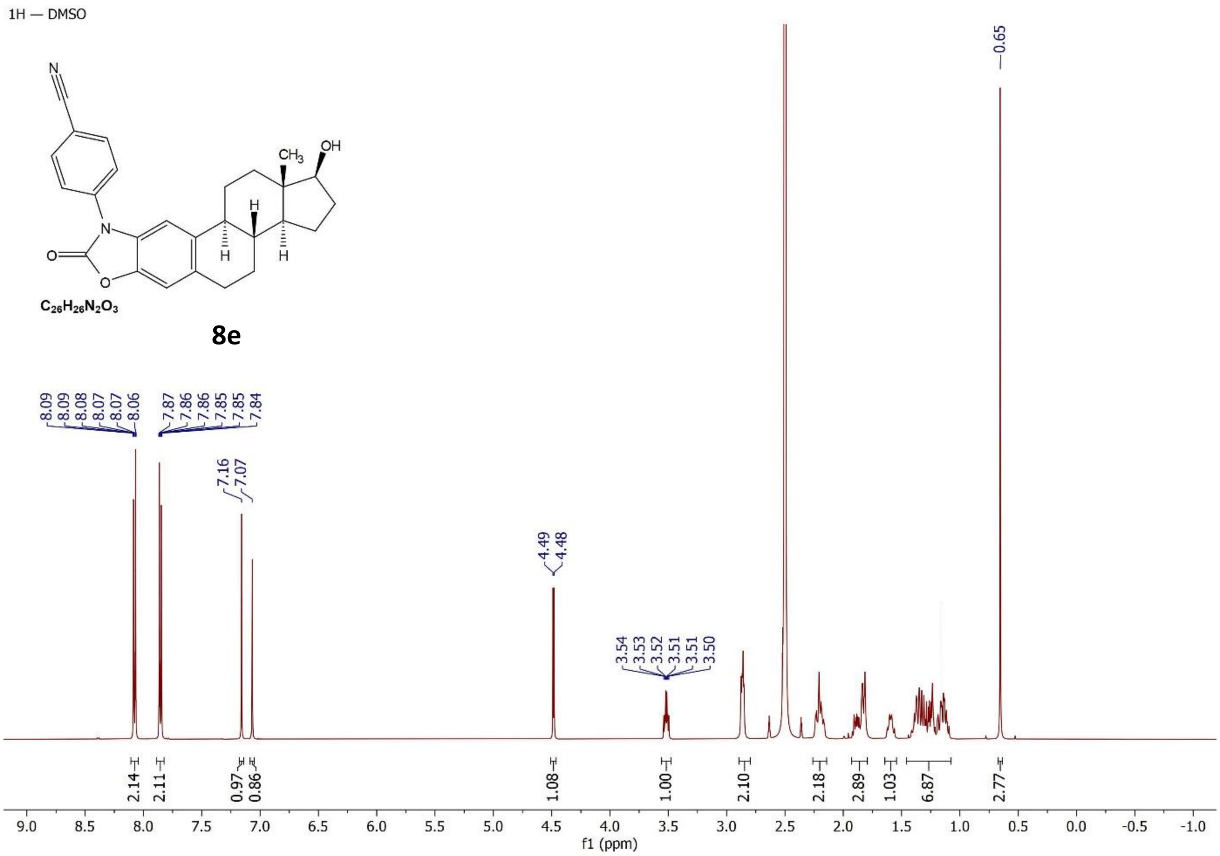

1H — DMSO
$C_{26}H_{26}N_2O_3$
8.09 8.09 8.08 8.07 8.07 8.06
7.87 7.86 7.86 7.85 7.85 7.84
7.16 7.07
4.49 4.48
3.54 3.53 3.52 3.51 3.51 3.50
0.65
2.14 2.11 0.97 0.86 1.08 1.00 2.10 2.18 2.89 1.03 6.87 2.77
f1 (ppm)


8e

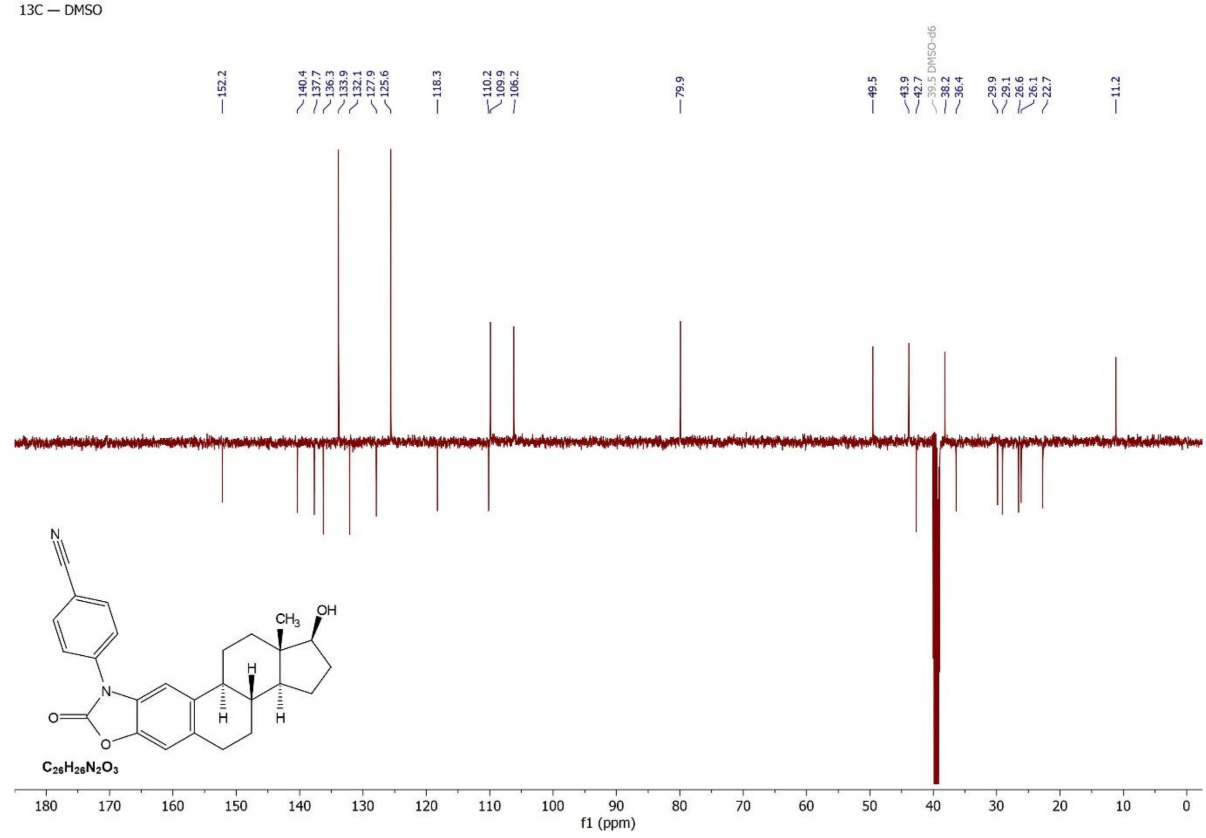

13C — DMSO
152.2
140.4 137.7 136.3 133.9 132.1 127.9 125.6
118.3
110.2 109.9 106.2
79.9
49.5
43.9 42.7 39.5 DMSO-d6 38.2 36.4
29.9 29.1 26.6 26.1 22.7
11.2
$C_{26}H_{26}N_2O_3$
f1 (ppm)

1H — DMSO

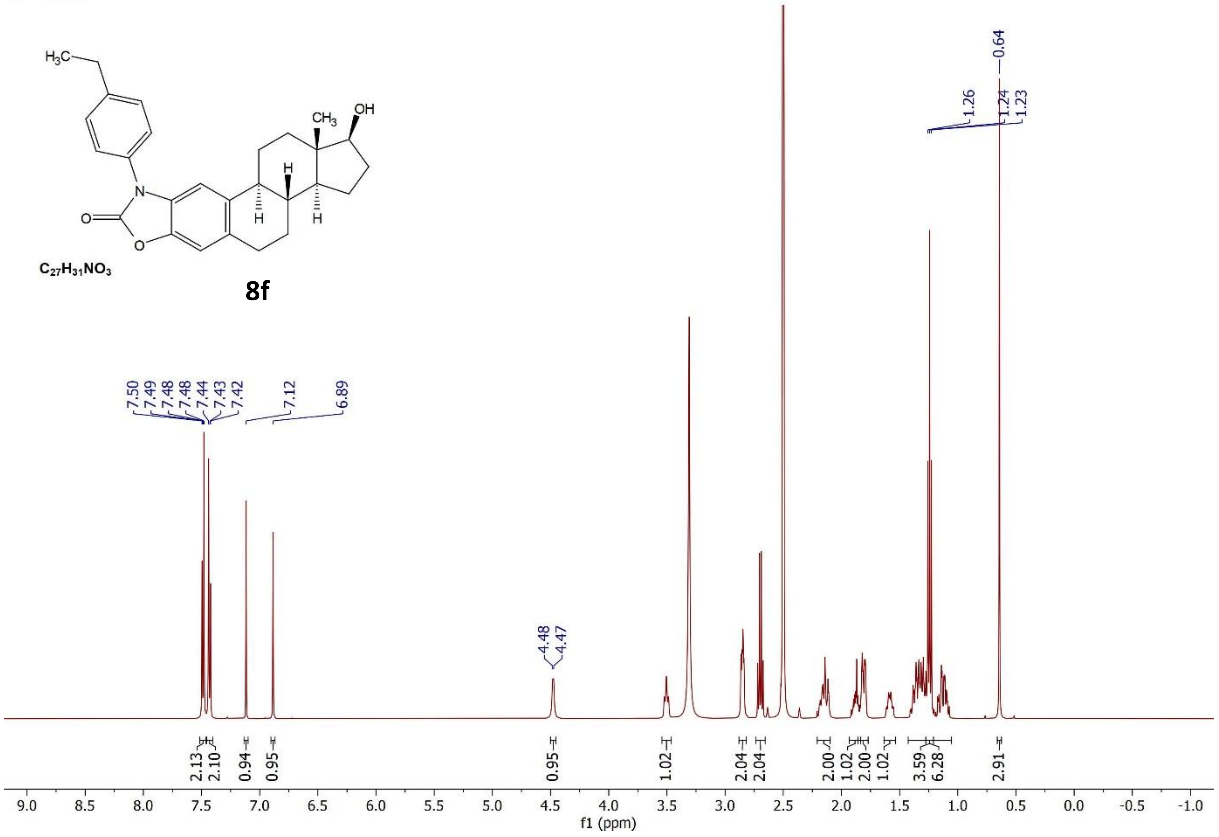


13C — DMSO

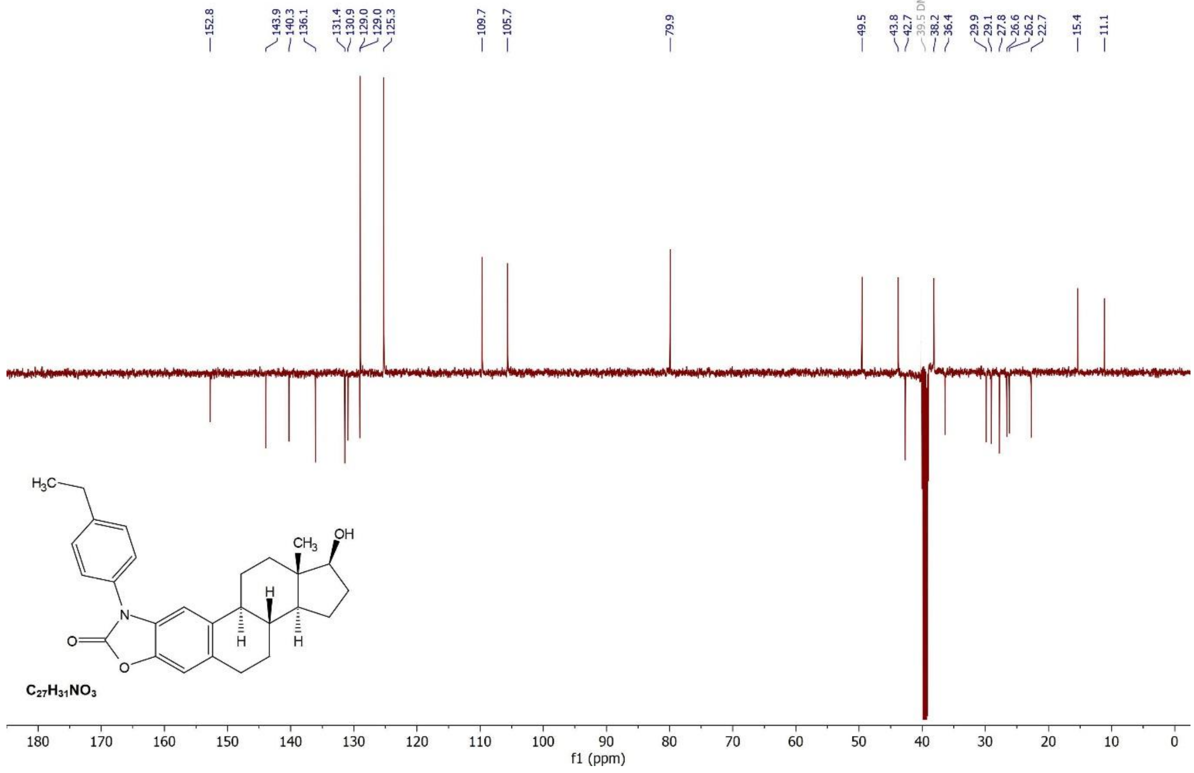

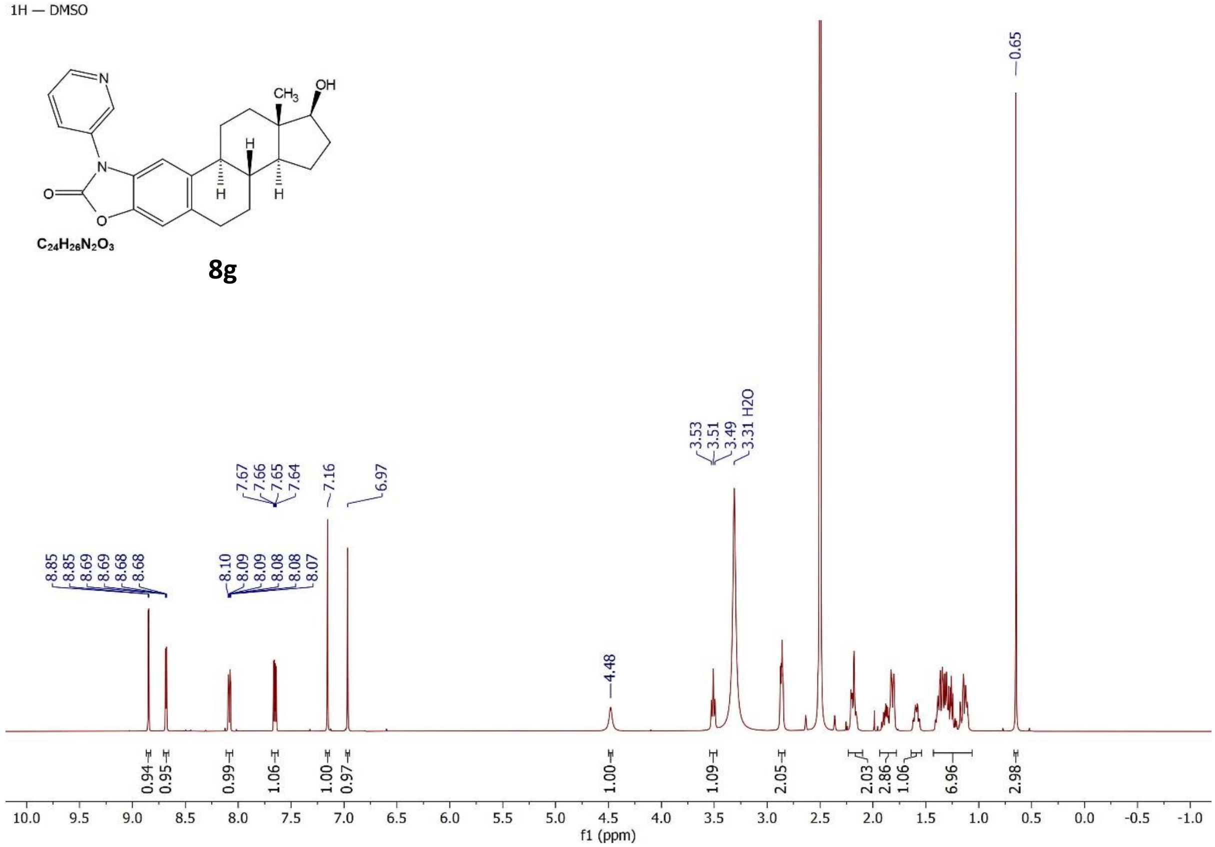
1H — DMSO
$C_{24}H_{26}N_2O_3$
8g
CH3
OH
8.85 8.85 8.69 8.69 8.68 8.68
8.10 8.09 8.09 8.08 8.08 8.07
7.67 7.66 7.65 7.64
7.16
6.97
4.48
3.53 3.51 3.49
3.31 H2O
0.65
0.94 0.95 0.99 1.06 1.00 0.97 1.00 1.09 2.05 2.03 2.86 1.06 6.96 2.98
f1 (ppm)

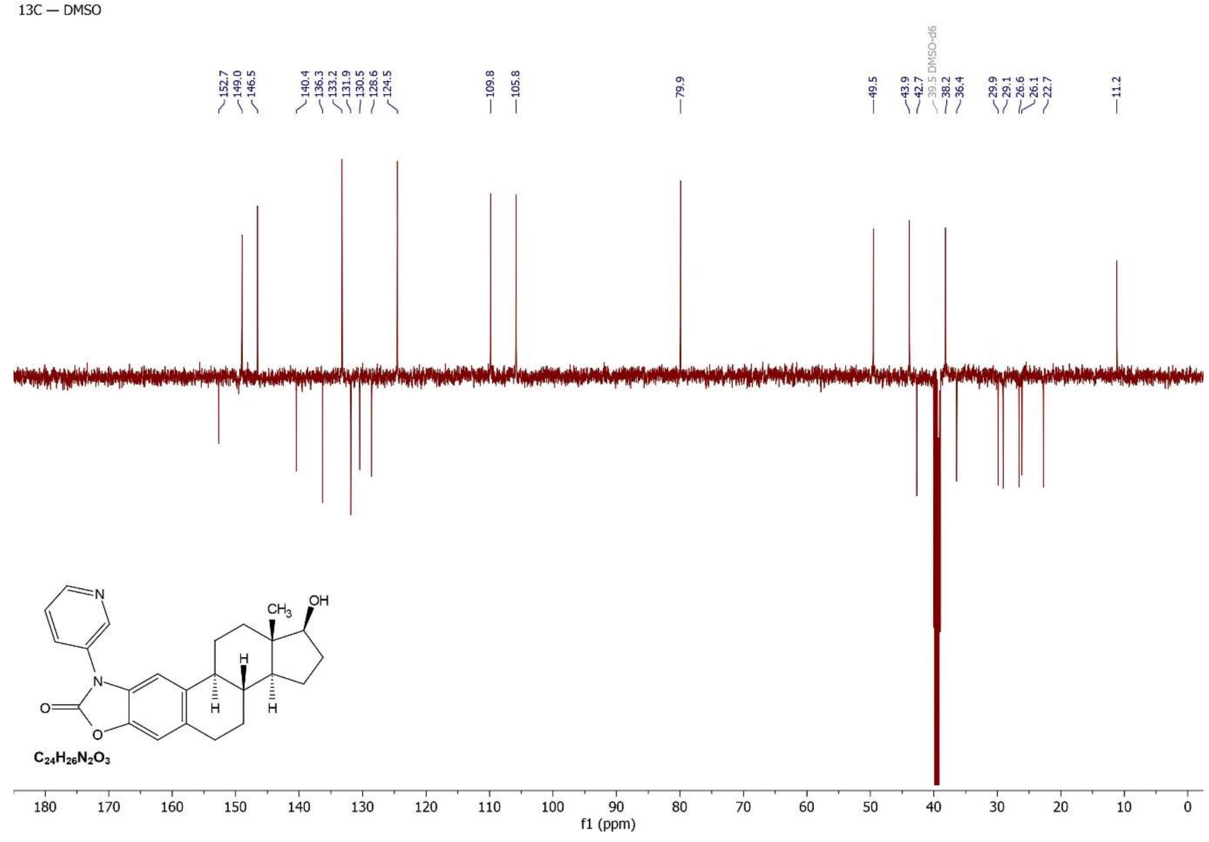
13C — DMSO
152.7 149.0 146.5 140.4 136.3 133.2 131.9 130.5 128.6 124.5 109.8 105.8 79.9 49.5 43.9 42.7 39.5 DMSO-d6 38.2 36.4 29.9 29.1 26.6 26.1 22.7 11.2
$C_{24}H_{26}N_2O_3$
f1 (ppm)

1H — DMSO

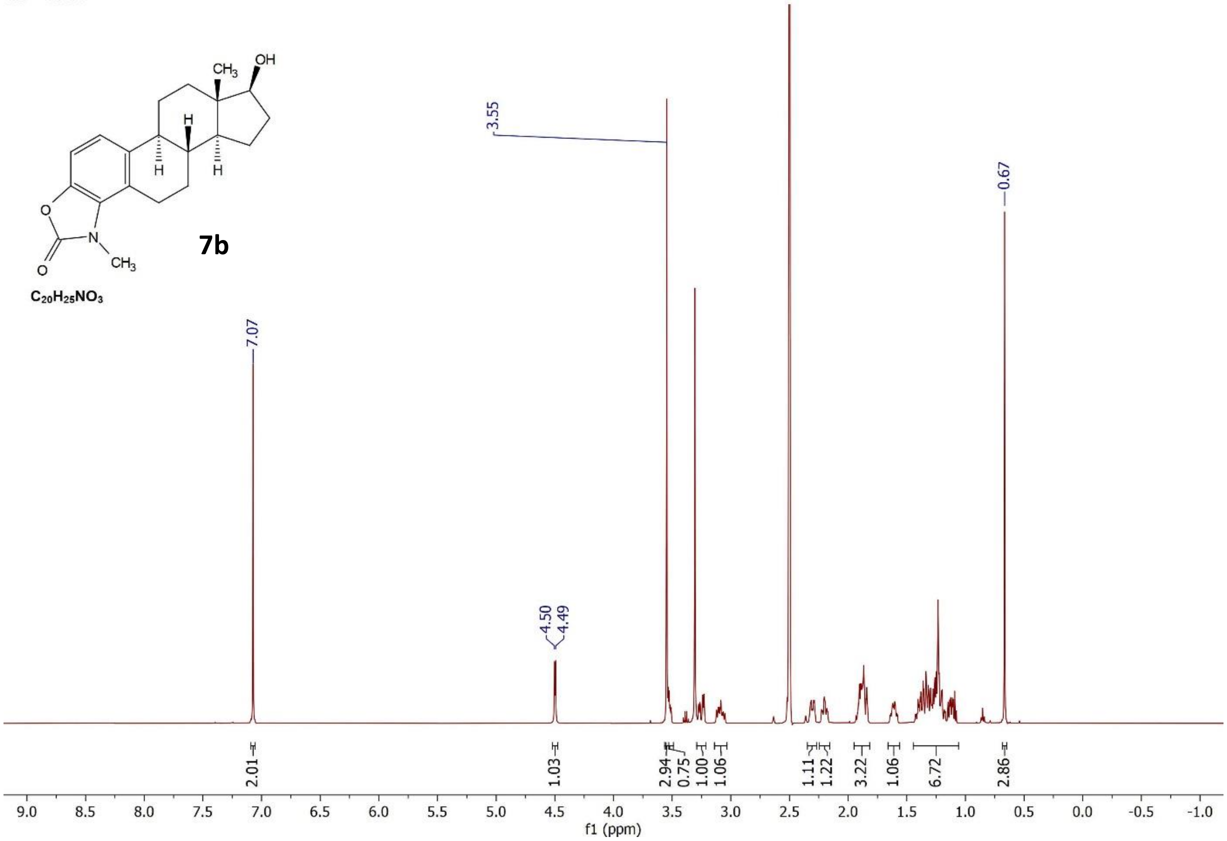


13C — DMSO

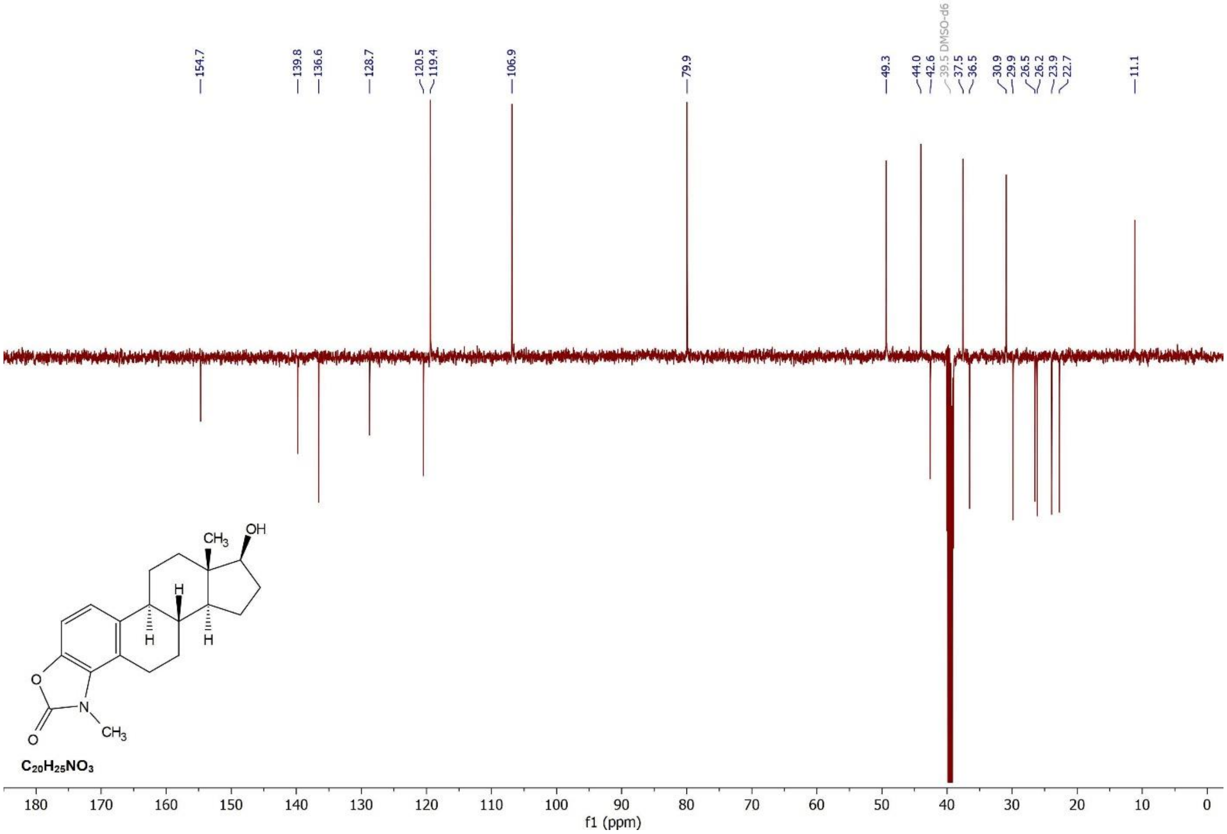

1H — DMSO

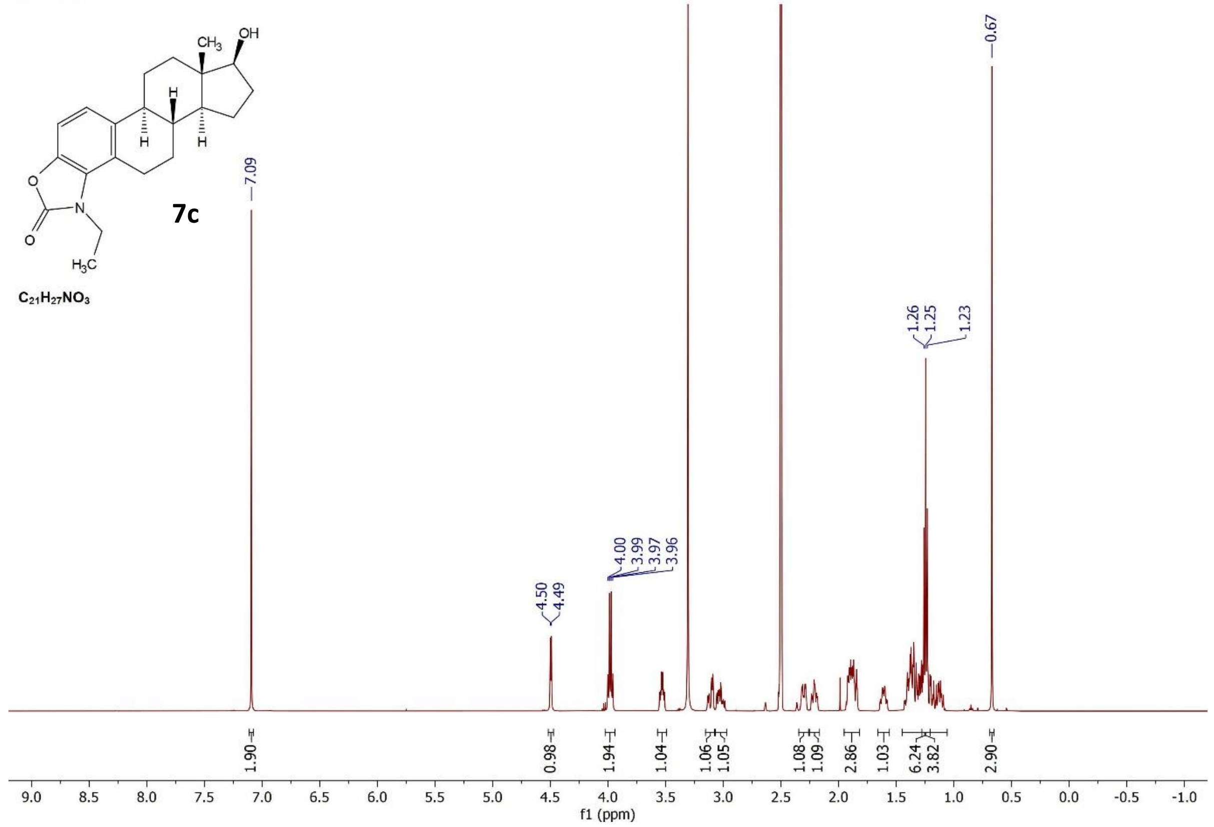


13C — DMSO

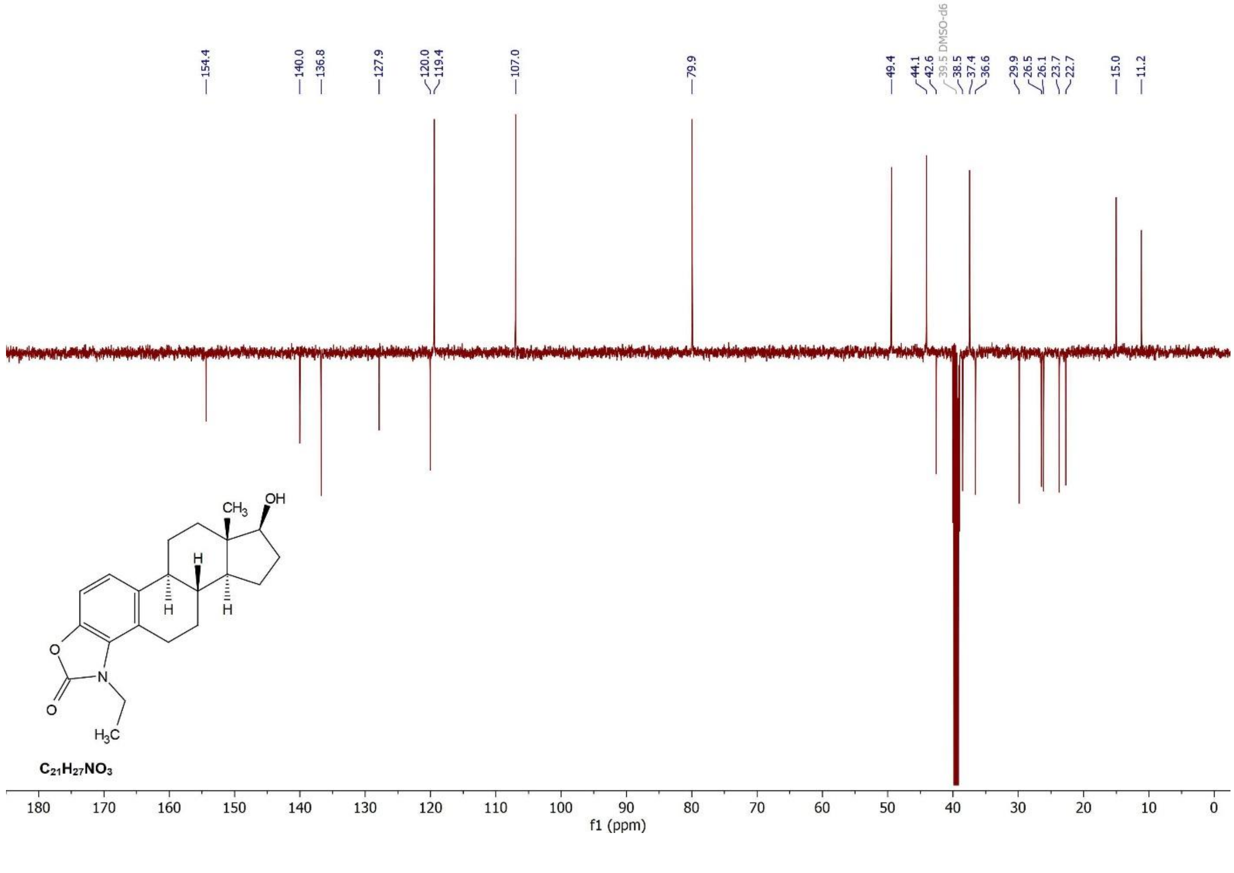

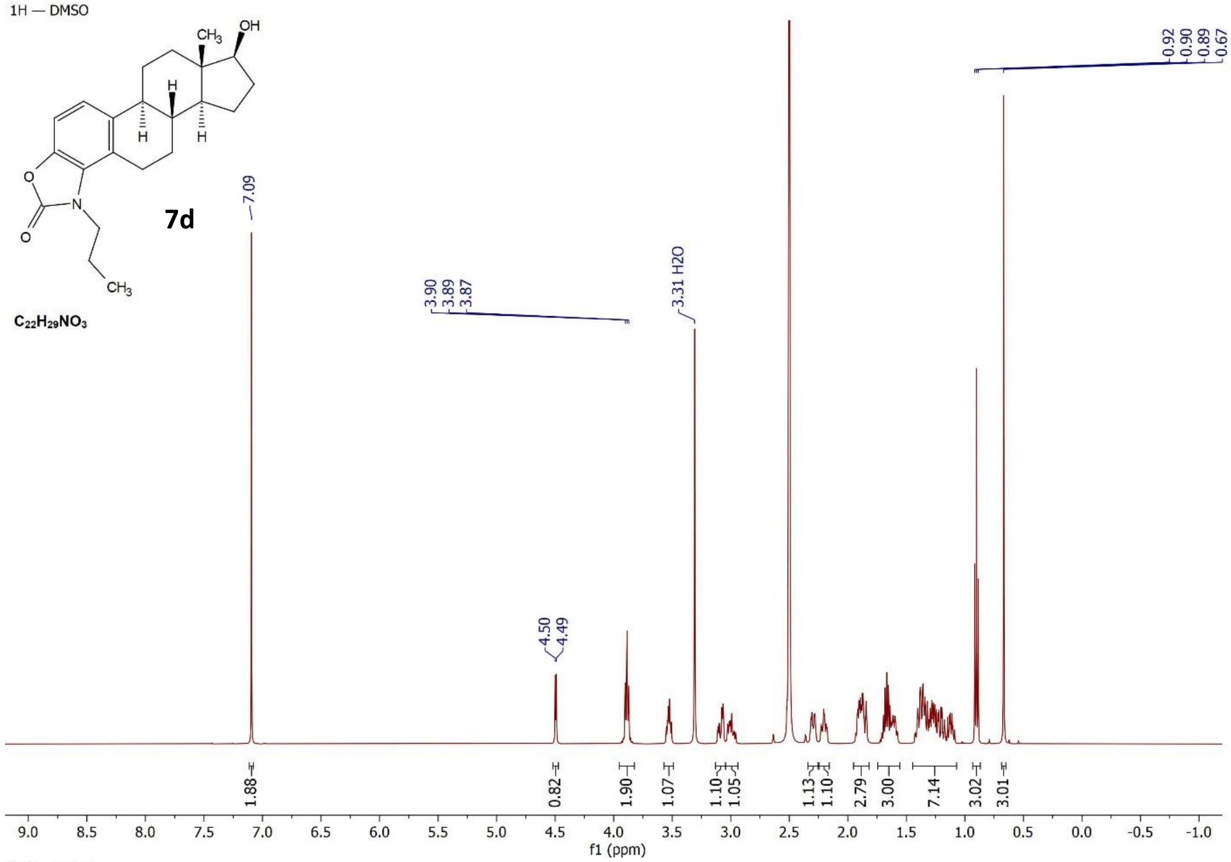
1H — DMSO
7d
$C_{22}H_{29}NO_3$
7.09
3.90
3.89
3.87
3.31 H2O
4.50
4.49
0.92
0.90
0.89
0.67
1.88
0.82
1.90
1.07
1.10
1.05
1.13
1.10
2.79
3.00
7.14
3.02
3.01
f1 (ppm)

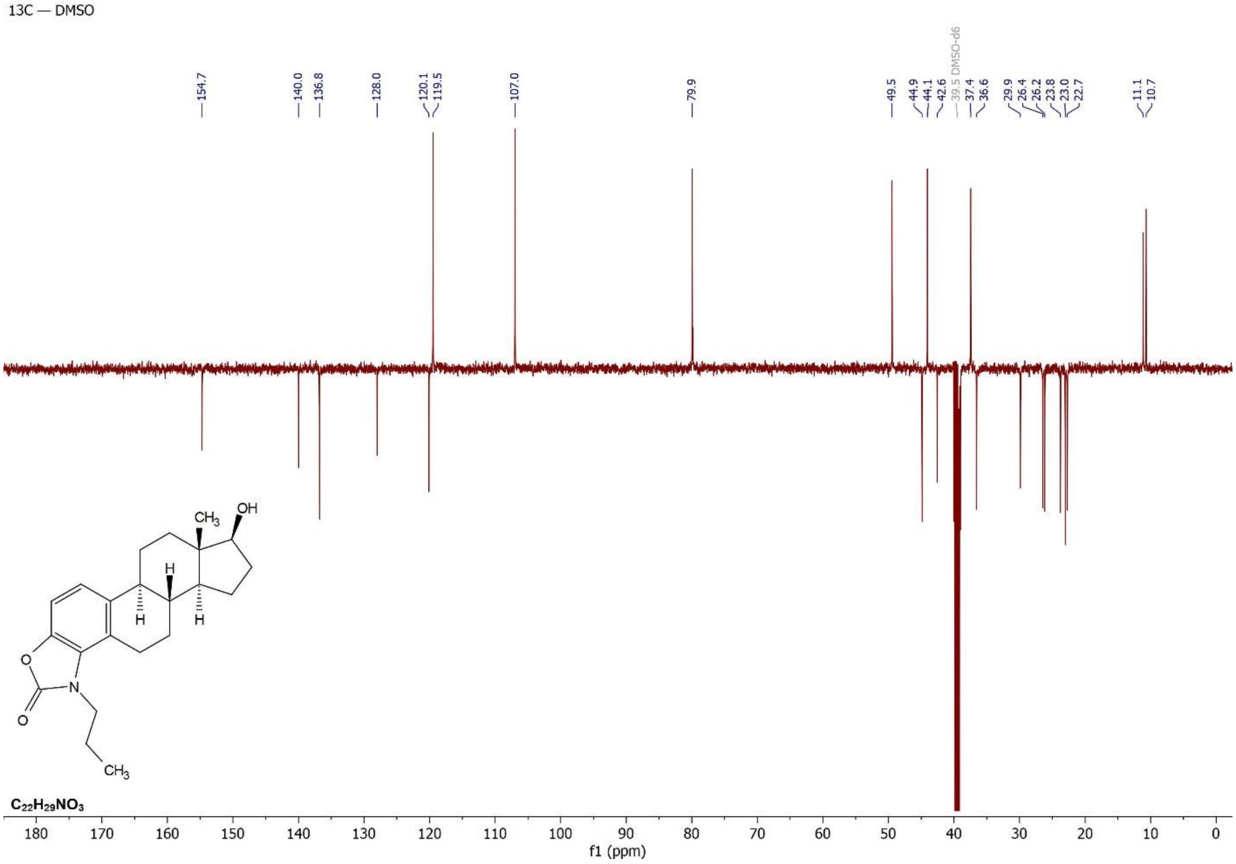
13C — DMSO
154.7
140.0
136.8
128.0
120.1
119.5
107.0
79.9
49.5
44.9
44.1
42.6
39.5 DMSO-d6
37.4
36.6
29.9
26.4
26.2
23.8
23.0
22.7
11.1
10.7
$C_{22}H_{29}NO_3$
f1 (ppm)

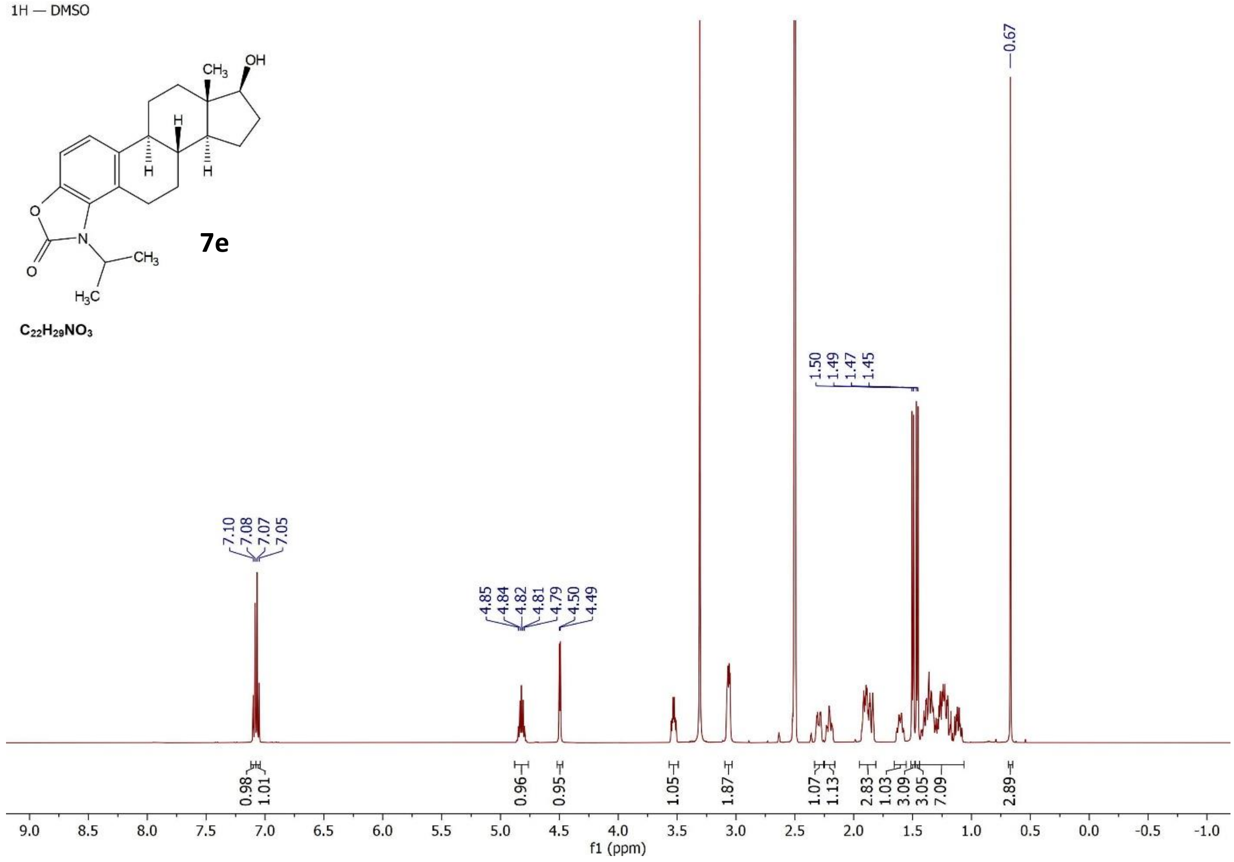
1H — DMSO
CH3
OH
H
H
H
O
N
O
CH3
H3C
7e
C22H29NO3
7.10
7.08
7.07
7.05
4.85
4.84
4.82
4.81
4.79
4.50
4.49
1.50
1.49
1.47
1.45
0.67
0.98
1.01
0.96
0.95
1.05
1.87
1.07
1.13
2.83
1.03
3.09
3.05
7.09
2.89
9.0 8.5 8.0 7.5 7.0 6.5 6.0 5.5 5.0 4.5 4.0 3.5 3.0 2.5 2.0 1.5 1.0 0.5 0.0 -0.5 -1.0
f1 (ppm)

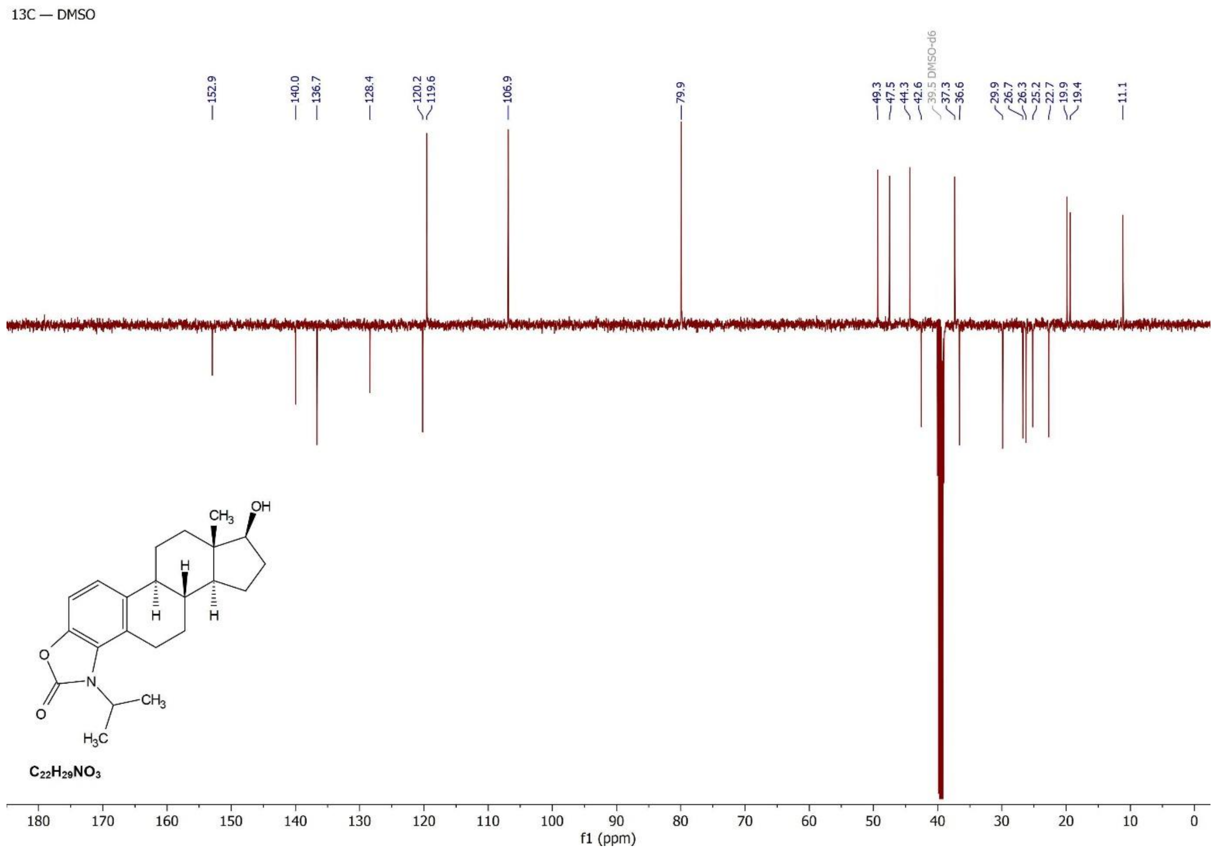
13C — DMSO
152.9
140.0
136.7
128.4
120.2
119.6
106.9
79.9
49.3
47.5
44.3
42.6
39.5 DMSO-d6
37.3
36.6
29.9
26.7
26.3
25.2
22.7
19.9
19.4
11.1
CH3
OH
H
H
H
O
N
O
CH3
H3C
C22H29NO3
180 170 160 150 140 130 120 110 100 90 80 70 60 50 40 30 20 10 0
f1 (ppm)

## Molecule 1

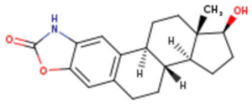

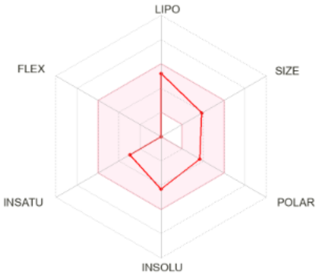


SMILES O[C@H]1CC[C@@H]2[C@]1(C)CC[C@H]1[C@H]2CCc2c1cc1[nH]c(=O)oc1c2

| Physicochemical Properties | |
|---|---|
| Formula | C19H23NO3 |
| Molecular weight | 313.39 g/mol |
| Num. heavy atoms | 23 |
| Num. arom. heavy atoms | 9 |
| Fraction Csp3 | 0.63 |
| Num. rotatable bonds | 0 |
| Num. H-bond acceptors | 3 |
| Num. H-bond donors | 2 |
| Molar Refractivity | 89.40 |
| TPSA | 66.23 Å² |
| **Lipophilicity** | |
| Log $P_{o/w}$ (iLOGP) | 2.73 |
| Log $P_{o/w}$ (XLOGP3) | 3.58 |
| Log $P_{o/w}$ (WLOGP) | 3.34 |
| Log $P_{o/w}$ (MLOGP) | 3.26 |
| Log $P_{o/w}$ (SILICOS-IT) | 3.71 |
| Consensus Log $P_{o/w}$ | 3.33 |

| Water Solubility | |
|---|---|
| Log *S* (ESOL) | -4.33 |
| Solubility | 1.47e-02 mg/ml ; 4.70e-05 mol/l |
| Class | Moderately soluble |
| Log *S* (Ali) | -4.66 |
| Solubility | 6.90e-03 mg/ml ; 2.20e-05 mol/l |
| Class | Moderately soluble |
| Log *S* (SILICOS-IT) | -4.73 |
| Solubility | 5.82e-03 mg/ml ; 1.86e-05 mol/l |
| Class | Moderately soluble |
| **Pharmacokinetics** | |
| GI absorption | High |
| BBB permeant | Yes |
| P-gp substrate | Yes |
| CYP1A2 inhibitor | No |
| CYP2C19 inhibitor | No |
| CYP2C9 inhibitor | No |
| CYP2D6 inhibitor | Yes |
| CYP3A4 inhibitor | No |
| Log $K_p$ (skin permeation) | -5.67 cm/s |
| **Druglikeness** | |
| Lipinski | Yes; 0 violation |
| Ghose | Yes |
| Veber | Yes |
| Egan | Yes |
| Muegge | Yes |
| Bioavailability Score | 0.55 |
| **Medicinal Chemistry** | |
| PAINS | 0 alert |
| Brenk | 0 alert |
| Leadlikeness | No; 1 violation: XLOGP3>3.5 |
| Synthetic accessibility | 4.44 |

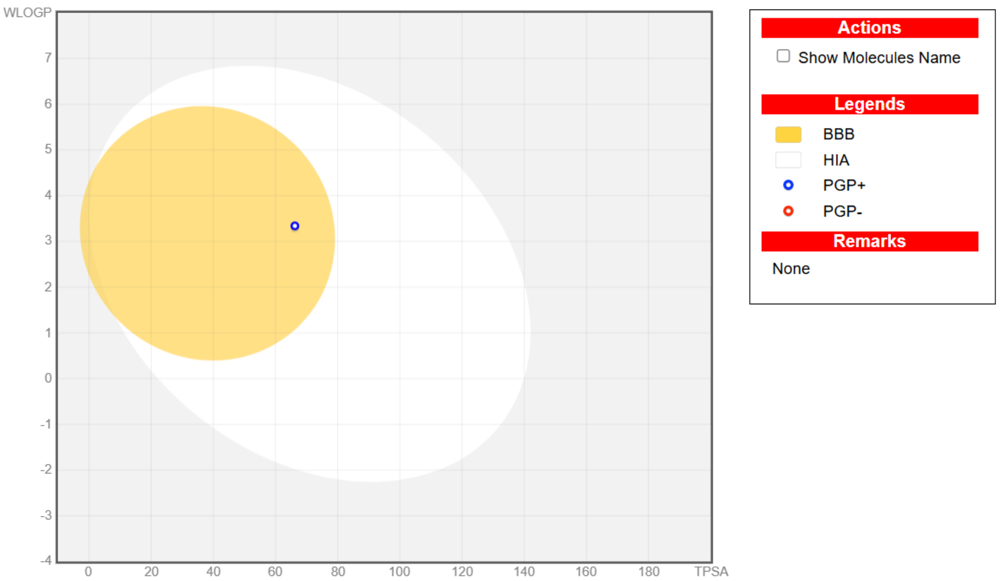


Molecule 1: compound **6a**

## Molecule 2

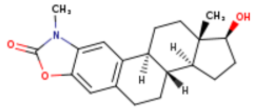

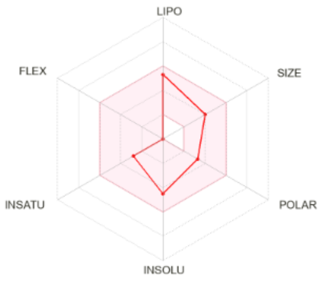


SMILES O[C@@H]1CC[C@@H]2[C@]1(C)CC[C@H]1[C@H]2CCc2c1cc1c(c2)oc(=O)n1C

| Physicochemical Properties | |
|---|---|
| Formula | C20H25NO3 |
| Molecular weight | 327.42 g/mol |
| Num. heavy atoms | 24 |
| Num. arom. heavy atoms | 9 |
| Fraction Csp3 | 0.65 |
| Num. rotatable bonds | 0 |
| Num. H-bond acceptors | 3 |
| Num. H-bond donors | 1 |
| Molar Refractivity | 94.30 |
| TPSA | 55.37 Å² |

| Lipophilicity | |
|---|---|
| Log $P_{o/w}$ (iLOGP) | 3.22 |
| Log $P_{o/w}$ (XLOGP3) | 3.76 |
| Log $P_{o/w}$ (WLOGP) | 3.35 |
| Log $P_{o/w}$ (MLOGP) | 3.49 |
| Log $P_{o/w}$ (SILICOS-IT) | 3.18 |
| Consensus Log $P_{o/w}$ | 3.40 |

| Water Solubility | |
|---|---|
| Log *S* (ESOL) | -4.52 |
| Solubility | 9.97e-03 mg/ml ; 3.05e-05 mol/l |
| Class | Moderately soluble |
| Log *S* (Ali) | -4.62 |
| Solubility | 7.93e-03 mg/ml ; 2.42e-05 mol/l |
| Class | Moderately soluble |
| Log *S* (SILICOS-IT) | -4.29 |
| Solubility | 1.70e-02 mg/ml ; 5.19e-05 mol/l |
| Class | Moderately soluble |

| Pharmacokinetics | |
|---|---|
| GI absorption | High |
| BBB permeant | Yes |
| P-gp substrate | Yes |
| CYP1A2 inhibitor | No |
| CYP2C19 inhibitor | No |
| CYP2C9 inhibitor | No |
| CYP2D6 inhibitor | Yes |
| CYP3A4 inhibitor | No |
| Log $K_p$ (skin permeation) | -5.63 cm/s |

| Druglikeness | |
|---|---|
| Lipinski | Yes; 0 violation |
| Ghose | Yes |
| Veber | Yes |
| Egan | Yes |
| Muegge | Yes |
| Bioavailability Score | 0.55 |

| Medicinal Chemistry | |
|---|---|
| PAINS | 0 alert |
| Brenk | 0 alert |
| Leadlikeness | No; 1 violation: XLOGP3>3.5 |
| Synthetic accessibility | 4.60 |

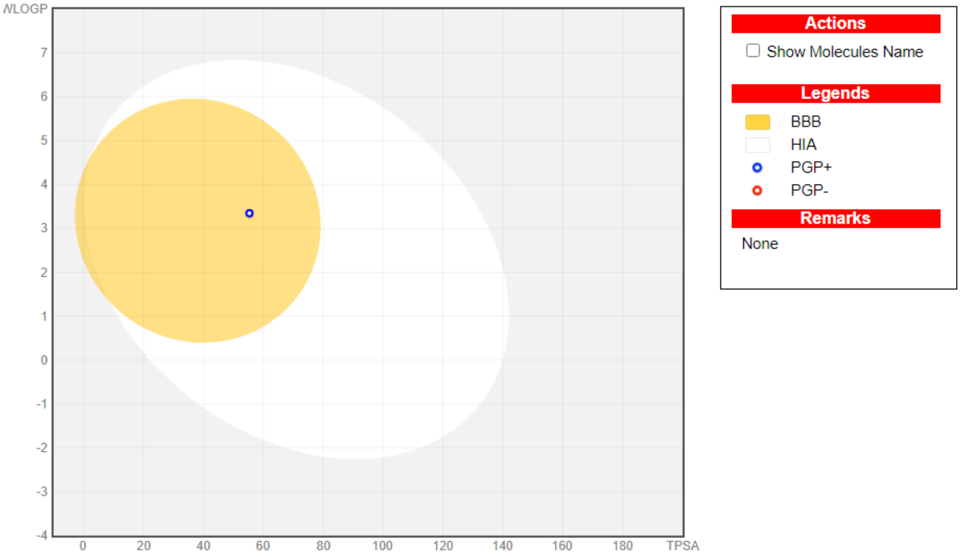


Molecule 2: compound **6b**

## Molecule 3

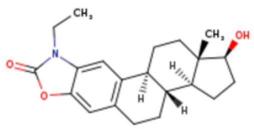

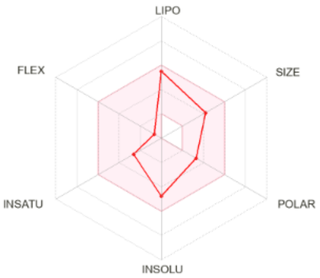


SMILES CCn1c(=O)oc2c1cc1c(c2)CC[C@@H]2[C@@H]1CC[C@]1([C@H]2CC[C@@H]1O)C

| Physicochemical Properties | |
|---|---|
| Formula | C21H27NO3 |
| Molecular weight | 341.44 g/mol |
| Num. heavy atoms | 25 |
| Num. arom. heavy atoms | 9 |
| Fraction Csp3 | 0.67 |
| Num. rotatable bonds | 1 |
| Num. H-bond acceptors | 3 |
| Num. H-bond donors | 1 |
| Molar Refractivity | 99.10 |
| TPSA | 55.37 Å² |

| Lipophilicity | |
|---|---|
| Log $P_{o/w}$ (iLOGP) | 3.45 |
| Log $P_{o/w}$ (XLOGP3) | 4.13 |
| Log $P_{o/w}$ (WLOGP) | 3.83 |
| Log $P_{o/w}$ (MLOGP) | 3.71 |
| Log $P_{o/w}$ (SILICOS-IT) | 3.56 |
| Consensus Log $P_{o/w}$ | 3.74 |

| Water Solubility | |
|---|---|
| Log *S* (ESOL) | -4.76 |
| Solubility | 5.94e-03 mg/ml ; 1.74e-05 mol/l |
| Class | Moderately soluble |
| Log *S* (Ali) | -5.00 |
| Solubility | 3.42e-03 mg/ml ; 1.00e-05 mol/l |
| Class | Moderately soluble |
| Log *S* (SILICOS-IT) | -4.68 |
| Solubility | 7.12e-03 mg/ml ; 2.08e-05 mol/l |
| Class | Moderately soluble |

| Pharmacokinetics | |
|---|---|
| GI absorption | High |
| BBB permeant | Yes |
| P-gp substrate | No |
| CYP1A2 inhibitor | No |
| CYP2C19 inhibitor | No |
| CYP2C9 inhibitor | Yes |
| CYP2D6 inhibitor | Yes |
| CYP3A4 inhibitor | No |
| Log $K_p$ (skin permeation) | -5.45 cm/s |

| Druglikeness | |
|---|---|
| Lipinski | Yes; 0 violation |
| Ghose | Yes |
| Veber | Yes |
| Egan | Yes |
| Muegge | Yes |
| Bioavailability Score | 0.55 |

| Medicinal Chemistry | |
|---|---|
| PAINS | 0 alert |
| Brenk | 0 alert |
| Leadlikeness | No; 1 violation: XLOGP3>3.5 |
| Synthetic accessibility | 4.76 |

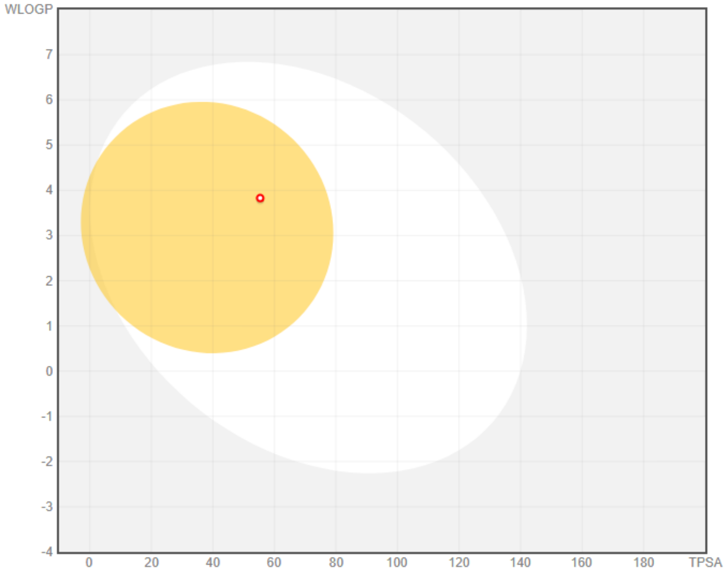


**Actions**

☐ Show Molecules Name

**Legends**

BBB
HIA
PGP+
PGP-

**Remarks**

None

Molecule 3: compound **6c**

## Molecule 4

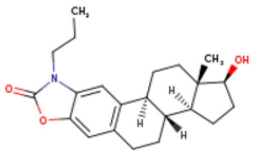


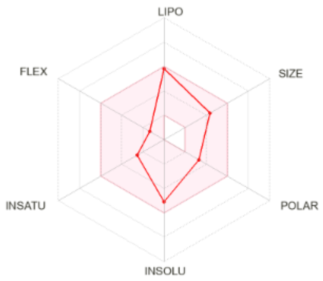


SMILES CCCn1c(=O)oc2c1cc1c(c2)CC[C@@H]2[C@@H]1CC[C@]1([C@H]2CC[C@@H]1O)C

| Physicochemical Properties | |
|---|---|
| Formula | C22H29NO3 |
| Molecular weight | 355.47 g/mol |
| Num. heavy atoms | 26 |
| Num. arom. heavy atoms | 9 |
| Fraction Csp3 | 0.68 |
| Num. rotatable bonds | 2 |
| Num. H-bond acceptors | 3 |
| Num. H-bond donors | 1 |
| Molar Refractivity | 103.91 |
| TPSA | 55.37 Å² |

| Lipophilicity | |
|---|---|
| Log $P_{o/w}$ (iLOGP) | 3.67 |
| Log $P_{o/w}$ (XLOGP3) | 4.66 |
| Log $P_{o/w}$ (WLOGP) | 4.22 |
| Log $P_{o/w}$ (MLOGP) | 3.93 |
| Log $P_{o/w}$ (SILICOS-IT) | 3.94 |
| Consensus Log $P_{o/w}$ | 4.09 |

| Water Solubility | |
|---|---|
| Log *S* (ESOL) | -5.10 |
| Solubility | 2.80e-03 mg/ml ; 7.87e-06 mol/l |
| Class | Moderately soluble |
| Log *S* (Ali) | -5.55 |
| Solubility | 1.00e-03 mg/ml ; 2.82e-06 mol/l |
| Class | Moderately soluble |
| Log *S* (SILICOS-IT) | -5.08 |
| Solubility | 2.98e-03 mg/ml ; 8.39e-06 mol/l |
| Class | Moderately soluble |

| Pharmacokinetics | |
|---|---|
| GI absorption | High |
| BBB permeant | Yes |
| P-gp substrate | No |
| CYP1A2 inhibitor | No |
| CYP2C19 inhibitor | Yes |
| CYP2C9 inhibitor | Yes |
| CYP2D6 inhibitor | Yes |
| CYP3A4 inhibitor | No |
| Log $K_p$ (skin permeation) | -5.16 cm/s |

| Druglikeness | |
|---|---|
| Lipinski | Yes; 0 violation |
| Ghose | Yes |
| Veber | Yes |
| Egan | Yes |
| Muegge | Yes |
| Bioavailability Score | 0.55 |

| Medicinal Chemistry | |
|---|---|
| PAINS | 0 alert |
| Brenk | 0 alert |
| Leadlikeness | No; 2 violations: MW>350, XLOGP3>3.5 |
| Synthetic accessibility | 4.91 |

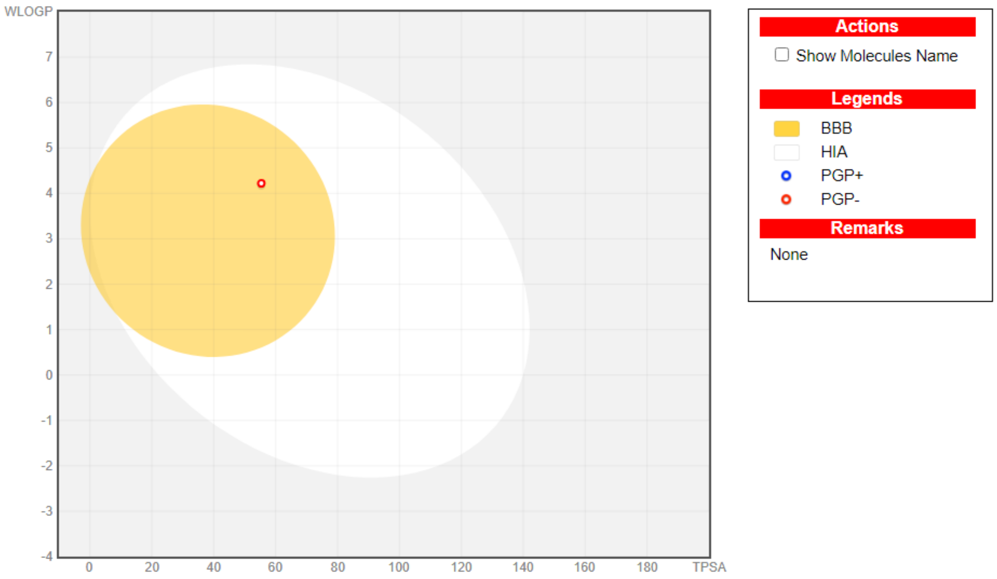


Molecule 4: compound **6d**

## Molecule 5

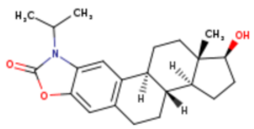

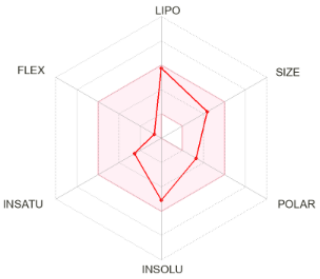


SMILES O[C@H]1CC[C@@H]2[C@]1(C)CC[C@H]1[C@H]2CCc2c1cc1c(c2)oc(=O)n1C(C)C

| Physicochemical Properties | |
|---|---|
| Formula | C22H29NO3 |
| Molecular weight | 355.47 g/mol |
| Num. heavy atoms | 26 |
| Num. arom. heavy atoms | 9 |
| Fraction Csp3 | 0.68 |
| Num. rotatable bonds | 1 |
| Num. H-bond acceptors | 3 |
| Num. H-bond donors | 1 |
| Molar Refractivity | 103.91 |
| TPSA | 55.37 Å² |

| Lipophilicity | |
|---|---|
| Log $P_{o/w}$ (iLOGP) | 3.66 |
| Log $P_{o/w}$ (XLOGP3) | 4.56 |
| Log $P_{o/w}$ (WLOGP) | 4.39 |
| Log $P_{o/w}$ (MLOGP) | 3.93 |
| Log $P_{o/w}$ (SILICOS-IT) | 3.77 |
| Consensus Log $P_{o/w}$ | 4.06 |

| Water Solubility | |
|---|---|
| Log *S* (ESOL) | -5.11 |
| Solubility | 2.78e-03 mg/ml ; 7.82e-06 mol/l |
| Class | Moderately soluble |
| Log *S* (Ali) | -5.45 |
| Solubility | 1.27e-03 mg/ml ; 3.58e-06 mol/l |
| Class | Moderately soluble |
| Log *S* (SILICOS-IT) | -4.70 |
| Solubility | 7.04e-03 mg/ml ; 1.98e-05 mol/l |
| Class | Moderately soluble |

| Pharmacokinetics | |
|---|---|
| GI absorption | High |
| BBB permeant | Yes |
| P-gp substrate | No |
| CYP1A2 inhibitor | No |
| CYP2C19 inhibitor | No |
| CYP2C9 inhibitor | Yes |
| CYP2D6 inhibitor | Yes |
| CYP3A4 inhibitor | No |
| Log $K_p$ (skin permeation) | -5.23 cm/s |

| Druglikeness | |
|---|---|
| Lipinski | Yes; 0 violation |
| Ghose | Yes |
| Veber | Yes |
| Egan | Yes |
| Muegge | Yes |
| Bioavailability Score | 0.55 |

| Medicinal Chemistry | |
|---|---|
| PAINS | 0 alert |
| Brenk | 0 alert |
| Leadlikeness | No; 2 violations: MW>350, XLOGP3>3.5 |
| Synthetic accessibility | 4.88 |

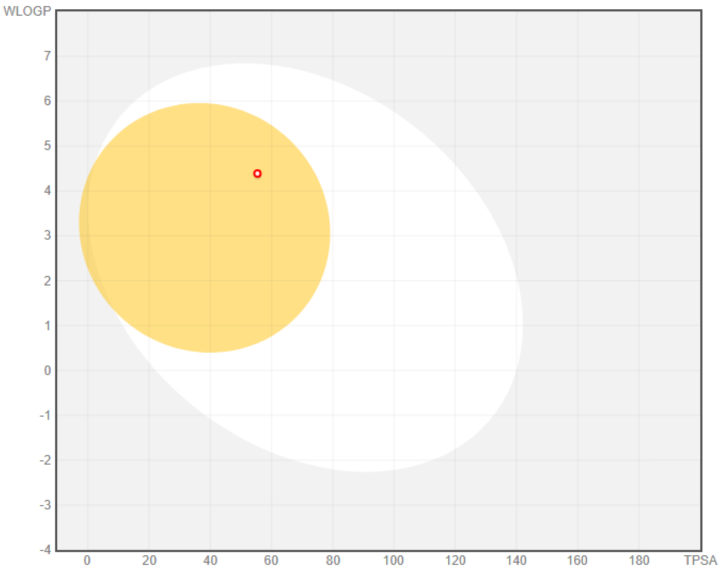


**Actions**

Show Molecules Name

**Legends**

BBB

HIA

PGP+

PGP-

**Remarks**

None

Molecule 5: compound **6e**

## Molecule 6

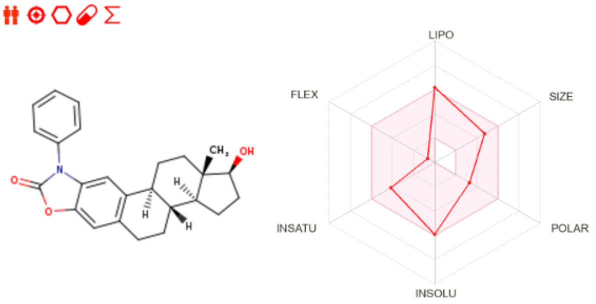


SMILES O[C@H]1CC[C@@H]2[C@]1(C)CC[C@H]1[C@H]2CCc2c1cc1c(c2)oc(=O)n1c1ccccc1

| Physicochemical Properties | |
|---|---|
| Formula | C25H27NO3 |
| Molecular weight | 389.49 g/mol |
| Num. heavy atoms | 29 |
| Num. arom. heavy atoms | 15 |
| Fraction Csp3 | 0.48 |
| Num. rotatable bonds | 1 |
| Num. H-bond acceptors | 3 |
| Num. H-bond donors | 1 |
| Molar Refractivity | 114.37 |
| TPSA | 55.37 Å² |

| Lipophilicity | |
|---|---|
| Log $P_{o/w}$ (iLOGP) | 3.73 |
| Log $P_{o/w}$ (XLOGP3) | 5.32 |
| Log $P_{o/w}$ (WLOGP) | 4.80 |
| Log $P_{o/w}$ (MLOGP) | 4.60 |
| Log $P_{o/w}$ (SILICOS-IT) | 4.21 |
| Consensus Log $P_{o/w}$ | 4.53 |

| Water Solubility | |
|---|---|
| Log *S* (ESOL) | -5.92 |
| Solubility | 4.65e-04 mg/ml ; 1.19e-06 mol/l |
| Class | Moderately soluble |
| Log *S* (Ali) | -6.23 |
| Solubility | 2.27e-04 mg/ml ; 5.83e-07 mol/l |
| Class | Poorly soluble |
| Log *S* (SILICOS-IT) | -6.37 |
| Solubility | 1.66e-04 mg/ml ; 4.27e-07 mol/l |
| Class | Poorly soluble |

| Pharmacokinetics | |
|---|---|
| GI absorption | High |
| BBB permeant | Yes |
| P-gp substrate | No |
| CYP1A2 inhibitor | No |
| CYP2C19 inhibitor | No |
| CYP2C9 inhibitor | Yes |
| CYP2D6 inhibitor | Yes |
| CYP3A4 inhibitor | No |
| Log $K_p$ (skin permeation) | -4.90 cm/s |

| Druglikeness | |
|---|---|
| Lipinski | Yes; 1 violation: MLOGP>4.15 |
| Ghose | Yes |
| Veber | Yes |
| Egan | Yes |
| Muegge | No; 1 violation: XLOGP3>5 |
| Bioavailability Score | 0.55 |

| Medicinal Chemistry | |
|---|---|
| PAINS | 0 alert |
| Brenk | 0 alert |
| Leadlikeness | No; 2 violations: MW>350, XLOGP3>3.5 |
| Synthetic accessibility | 4.96 |

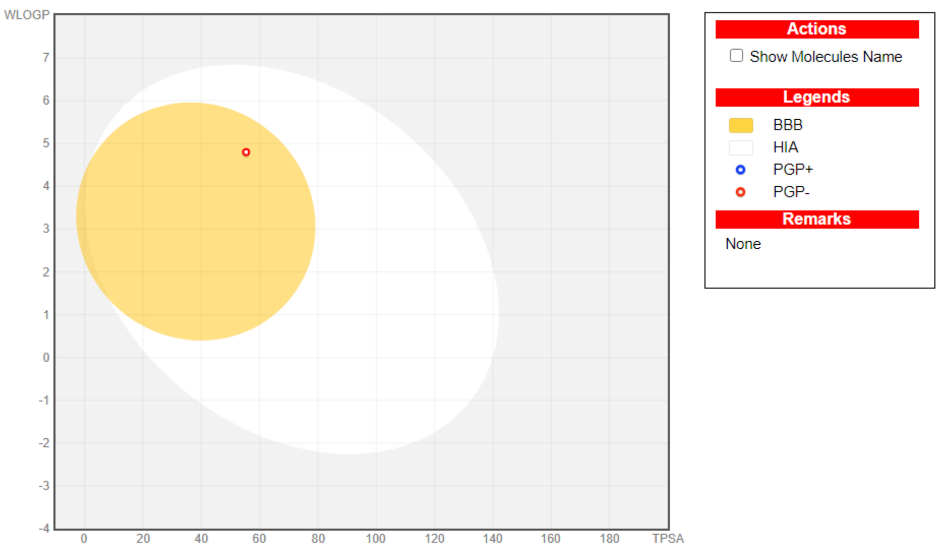


Molecule 6: compound **8a**

## Molecule 7

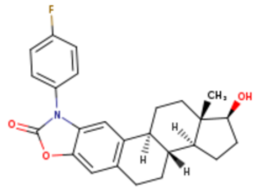


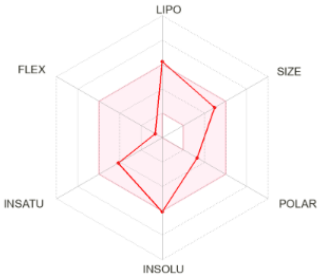


SMILES Fc1ccc(cc1)n1c(=O)oc2c1cc1c(c2)CC[C@@H]2[C@@H]1CC[C@]1([C@H]2CC[C@@H]1O)C

| Physicochemical Properties | |
|---|---|
| Formula | C25H26FNO3 |
| Molecular weight | 407.48 g/mol |
| Num. heavy atoms | 30 |
| Num. arom. heavy atoms | 15 |
| Fraction Csp3 | 0.48 |
| Num. rotatable bonds | 1 |
| Num. H-bond acceptors | 4 |
| Num. H-bond donors | 1 |
| Molar Refractivity | 114.33 |
| TPSA | 55.37 Å² |

| Lipophilicity | |
|---|---|
| Log $P_{o/w}$ (iLOGP) | 3.88 |
| Log $P_{o/w}$ (XLOGP3) | 5.42 |
| Log $P_{o/w}$ (WLOGP) | 5.36 |
| Log $P_{o/w}$ (MLOGP) | 4.97 |
| Log $P_{o/w}$ (SILICOS-IT) | 4.62 |
| Consensus Log $P_{o/w}$ | 4.85 |

| Water Solubility | |
|---|---|
| Log *S* (ESOL) | -6.08 |
| Solubility | 3.35e-04 mg/ml ; 8.22e-07 mol/l |
| Class | Poorly soluble |
| Log *S* (Ali) | -6.34 |
| Solubility | 1.87e-04 mg/ml ; 4.59e-07 mol/l |
| Class | Poorly soluble |
| Log *S* (SILICOS-IT) | -6.63 |
| Solubility | 9.46e-05 mg/ml ; 2.32e-07 mol/l |
| Class | Poorly soluble |

| Pharmacokinetics | |
|---|---|
| GI absorption | High |
| BBB permeant | Yes |
| P-gp substrate | No |
| CYP1A2 inhibitor | No |
| CYP2C19 inhibitor | No |
| CYP2C9 inhibitor | Yes |
| CYP2D6 inhibitor | Yes |
| CYP3A4 inhibitor | No |
| Log $K_p$ (skin permeation) | -4.94 cm/s |

| Druglikeness | |
|---|---|
| Lipinski | Yes; 1 violation: MLOGP>4.15 |
| Ghose | Yes |
| Veber | Yes |
| Egan | Yes |
| Muegge | No; 1 violation: XLOGP3>5 |
| Bioavailability Score | 0.55 |

| Medicinal Chemistry | |
|---|---|
| PAINS | 0 alert |
| Brenk | 0 alert |
| Leadlikeness | No; 2 violations: MW>350, XLOGP3>3.5 |
| Synthetic accessibility | 4.93 |

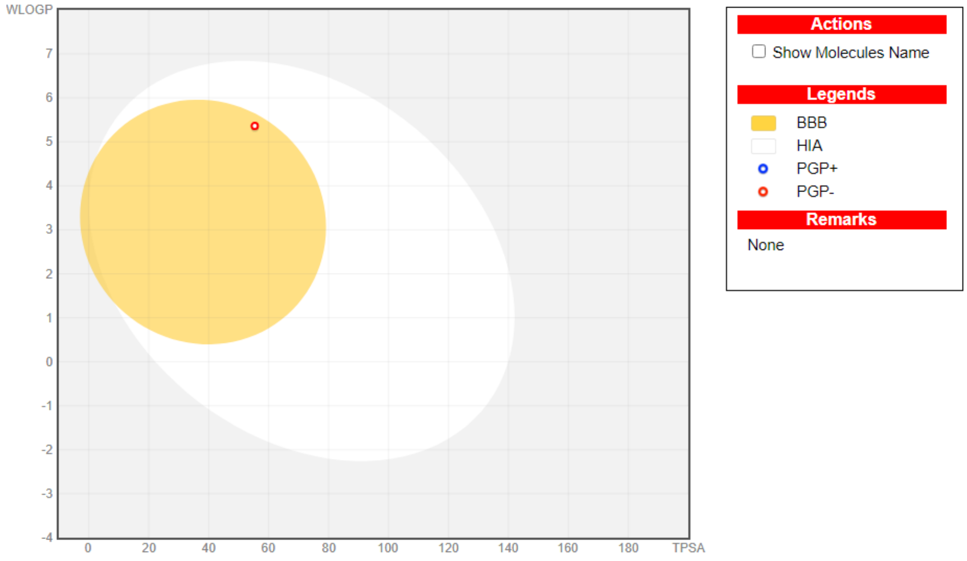


Molecule 7: compound **8b**

## Molecule 8

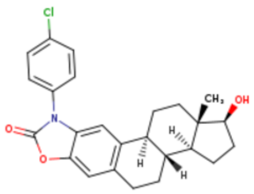


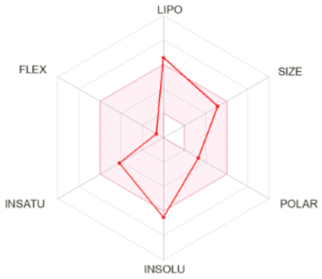


SMILES Clc1ccc(cc1)n1c(=O)oc2c1cc1c(c2)CC[C@@H]2[C@@H]1CC[C@]1([C@H]2CC[C@@H]1O)C

| Physicochemical Properties | |
|---|---|
| Formula | C25H26ClNO3 |
| Molecular weight | 423.93 g/mol |
| Num. heavy atoms | 30 |
| Num. arom. heavy atoms | 15 |
| Fraction Csp3 | 0.48 |
| Num. rotatable bonds | 1 |
| Num. H-bond acceptors | 3 |
| Num. H-bond donors | 1 |
| Molar Refractivity | 119.38 |
| TPSA | 55.37 Å² |

| Lipophilicity | |
|---|---|
| Log $P_{o/w}$ (iLOGP) | 4.05 |
| Log $P_{o/w}$ (XLOGP3) | 5.95 |
| Log $P_{o/w}$ (WLOGP) | 5.45 |
| Log $P_{o/w}$ (MLOGP) | 5.07 |
| Log $P_{o/w}$ (SILICOS-IT) | 4.84 |
| Consensus Log $P_{o/w}$ | 5.07 |

| Water Solubility | |
|---|---|
| Log *S* (ESOL) | -6.52 |
| Solubility | 1.28e-04 mg/ml ; 3.01e-07 mol/l |
| Class | Poorly soluble |
| Log *S* (Ali) | -6.89 |
| Solubility | 5.48e-05 mg/ml ; 1.29e-07 mol/l |
| Class | Poorly soluble |
| Log *S* (SILICOS-IT) | -6.96 |
| Solubility | 4.69e-05 mg/ml ; 1.11e-07 mol/l |
| Class | Poorly soluble |

| Pharmacokinetics | |
|---|---|
| GI absorption | High |
| BBB permeant | Yes |
| P-gp substrate | No |
| CYP1A2 inhibitor | No |
| CYP2C19 inhibitor | No |
| CYP2C9 inhibitor | Yes |
| CYP2D6 inhibitor | Yes |
| CYP3A4 inhibitor | No |
| Log $K_p$ (skin permeation) | -4.66 cm/s |

| Druglikeness | |
|---|---|
| Lipinski | Yes; 1 violation: MLOGP>4.15 |
| Ghose | Yes |
| Veber | Yes |
| Egan | Yes |
| Muegge | No; 1 violation: XLOGP3>5 |
| Bioavailability Score | 0.55 |

| Medicinal Chemistry | |
|---|---|
| PAINS | 0 alert |
| Brenk | 0 alert |
| Leadlikeness | No; 2 violations: MW>350, XLOGP3>3.5 |
| Synthetic accessibility | 4.95 |

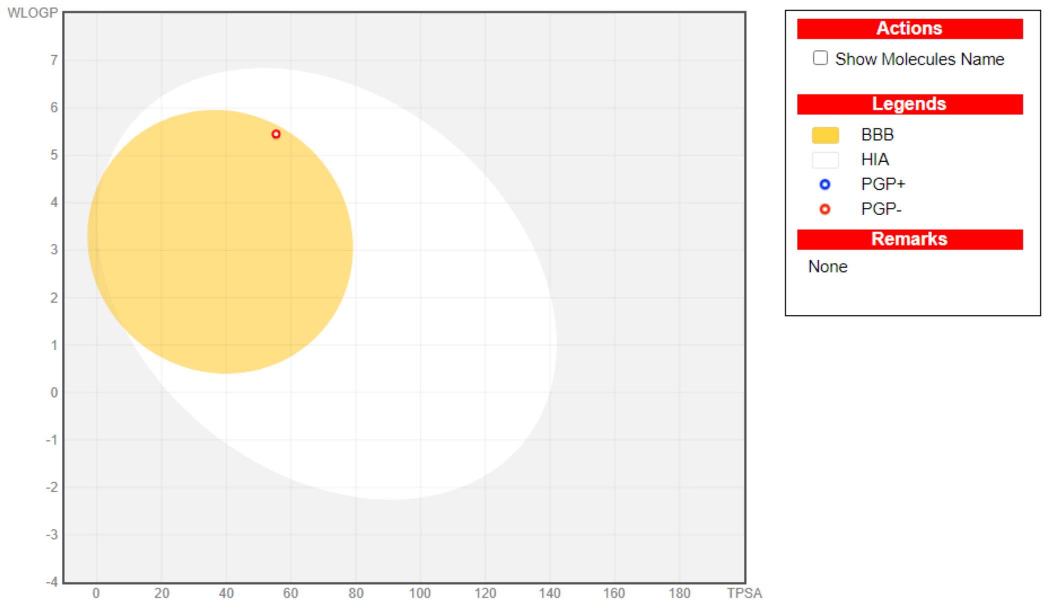


Molecule 8: compound **8c**

## Molecule 9

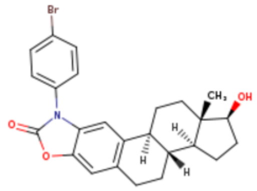


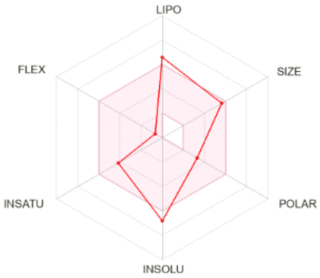


SMILES Brc1ccc(cc1)n1c(=O)oc2c1cc1c(c2)CC[C@@H]2[C@@H]1CC[C@]1([C@H]2CC[C@@H]1O)C

| Physicochemical Properties | |
|---|---|
| Formula | C25H26BrNO3 |
| Molecular weight | 468.38 g/mol |
| Num. heavy atoms | 30 |
| Num. arom. heavy atoms | 15 |
| Fraction Csp3 | 0.48 |
| Num. rotatable bonds | 1 |
| Num. H-bond acceptors | 3 |
| Num. H-bond donors | 1 |
| Molar Refractivity | 122.07 |
| TPSA | 55.37 Å² |

| Lipophilicity | |
|---|---|
| Log $P_{o/w}$ (iLOGP) | 4.13 |
| Log $P_{o/w}$ (XLOGP3) | 6.01 |
| Log $P_{o/w}$ (WLOGP) | 5.56 |
| Log $P_{o/w}$ (MLOGP) | 5.17 |
| Log $P_{o/w}$ (SILICOS-IT) | 4.88 |
| Consensus Log $P_{o/w}$ | 5.15 |

| Water Solubility | |
|---|---|
| Log *S* (ESOL) | -6.83 |
| Solubility | 6.86e-05 mg/ml ; 1.46e-07 mol/l |
| Class | Poorly soluble |
| Log *S* (Ali) | -6.95 |
| Solubility | 5.25e-05 mg/ml ; 1.12e-07 mol/l |
| Class | Poorly soluble |
| Log *S* (SILICOS-IT) | -7.15 |
| Solubility | 3.33e-05 mg/ml ; 7.11e-08 mol/l |
| Class | Poorly soluble |

| Pharmacokinetics | |
|---|---|
| GI absorption | High |
| BBB permeant | Yes |
| P-gp substrate | No |
| CYP1A2 inhibitor | No |
| CYP2C19 inhibitor | No |
| CYP2C9 inhibitor | Yes |
| CYP2D6 inhibitor | Yes |
| CYP3A4 inhibitor | No |
| Log $K_p$ (skin permeation) | -4.89 cm/s |

| Druglikeness | |
|---|---|
| Lipinski | Yes; 1 violation: MLOGP>4.15 |
| Ghose | Yes |
| Veber | Yes |
| Egan | Yes |
| Muegge | No; 1 violation: XLOGP3>5 |
| Bioavailability Score | 0.55 |

| Medicinal Chemistry | |
|---|---|
| PAINS | 0 alert |
| Brenk | 0 alert |
| Leadlikeness | No; 2 violations: MW>350, XLOGP3>3.5 |
| Synthetic accessibility | 4.97 |

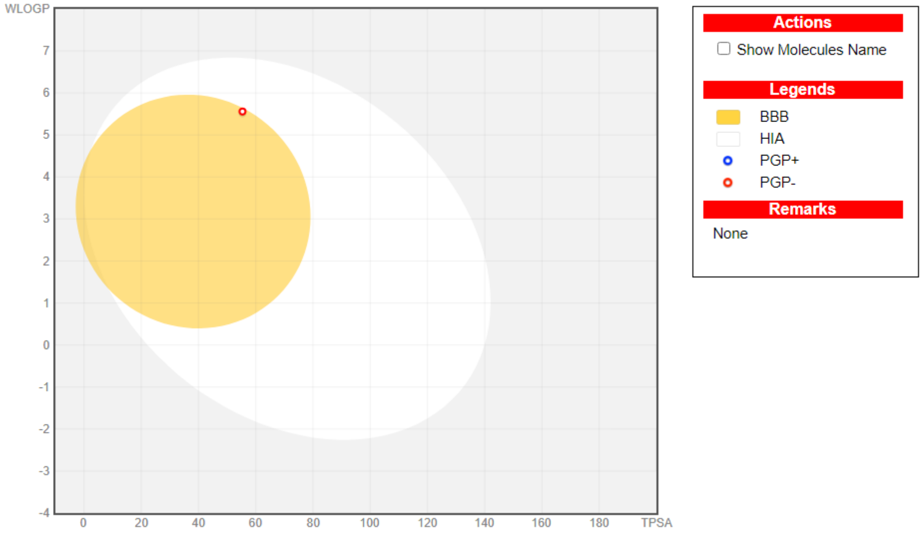


Molecule 9: compound **8d**

## Molecule 10

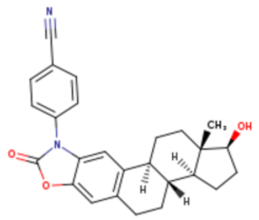

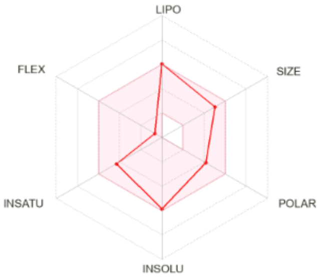


SMILES N#Cc1ccc(cc1)n1c(=O)oc2c1cc1c(c2)CC[C@@H]2[C@@H]1CC[C@]1([C@H]2CC[C@@H]1O)C

| Physicochemical Properties | |
|---|---|
| Formula | C26H26N2O3 |
| Molecular weight | 414.50 g/mol |
| Num. heavy atoms | 31 |
| Num. arom. heavy atoms | 15 |
| Fraction Csp3 | 0.46 |
| Num. rotatable bonds | 1 |
| Num. H-bond acceptors | 4 |
| Num. H-bond donors | 1 |
| Molar Refractivity | 119.09 |
| TPSA | 79.16 Å² |

| Lipophilicity | |
|---|---|
| Log $P_{o/w}$ (iLOGP) | 3.66 |
| Log $P_{o/w}$ (XLOGP3) | 5.04 |
| Log $P_{o/w}$ (WLOGP) | 4.67 |
| Log $P_{o/w}$ (MLOGP) | 3.90 |
| Log $P_{o/w}$ (SILICOS-IT) | 4.24 |
| Consensus Log $P_{o/w}$ | 4.30 |

| Water Solubility | |
|---|---|
| Log *S* (ESOL) | -5.88 |
| Solubility | 5.50e-04 mg/ml ; 1.33e-06 mol/l |
| Class | Moderately soluble |
| Log *S* (Ali) | -6.44 |
| Solubility | 1.49e-04 mg/ml ; 3.60e-07 mol/l |
| Class | Poorly soluble |
| Log *S* (SILICOS-IT) | -6.43 |
| Solubility | 1.52e-04 mg/ml ; 3.67e-07 mol/l |
| Class | Poorly soluble |

| Pharmacokinetics | |
|---|---|
| GI absorption | High |
| BBB permeant | No |
| P-gp substrate | No |
| CYP1A2 inhibitor | No |
| CYP2C19 inhibitor | No |
| CYP2C9 inhibitor | Yes |
| CYP2D6 inhibitor | Yes |
| CYP3A4 inhibitor | No |
| Log $K_p$ (skin permeation) | -5.25 cm/s |

| Druglikeness | |
|---|---|
| Lipinski | Yes; 0 violation |
| Ghose | Yes |
| Veber | Yes |
| Egan | Yes |
| Muegge | No; 1 violation: XLOGP3>5 |
| Bioavailability Score | 0.55 |

| Medicinal Chemistry | |
|---|---|
| PAINS | 0 alert |
| Brenk | 0 alert |
| Leadlikeness | No; 2 violations: MW>350, XLOGP3>3.5 |
| Synthetic accessibility | 5.07 |

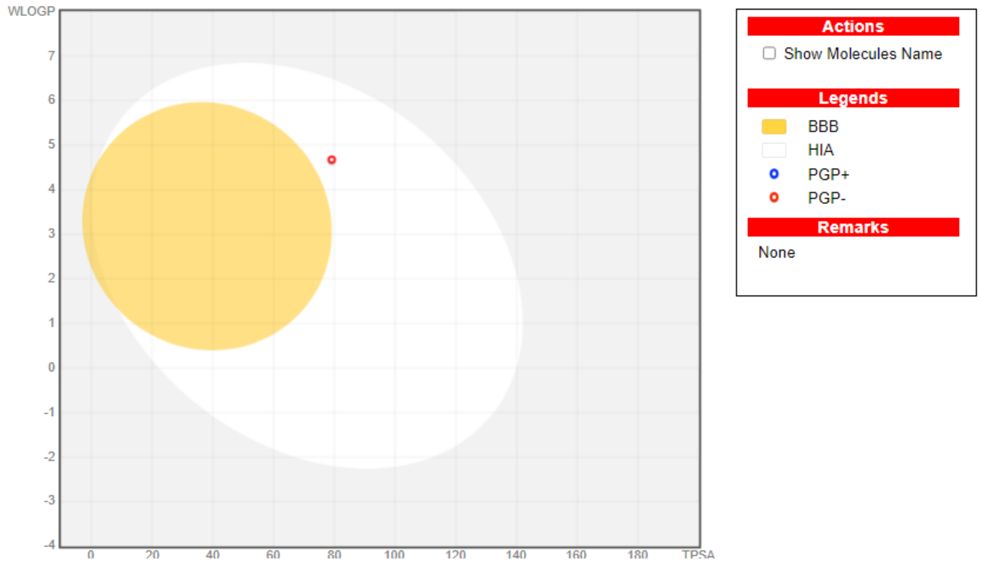


Molecule 10: compound **8e**

## Molecule 11

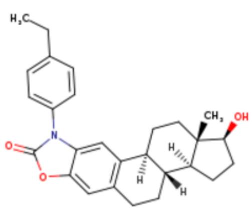

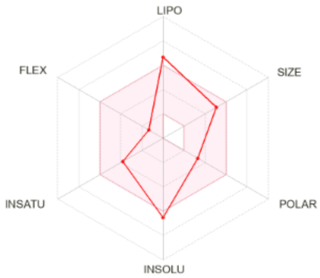


SMILES CCc1ccc(cc1)n1c(=O)oc2c1cc1c(c2)CC[C@@H]2[C@@H]1CC[C@]1([C@H]2CC[C@@H]1O)C

| Physicochemical Properties | |
|---|---|
| Formula | C27H31NO3 |
| Molecular weight | 417.54 g/mol |
| Num. heavy atoms | 31 |
| Num. arom. heavy atoms | 15 |
| Fraction Csp3 | 0.52 |
| Num. rotatable bonds | 2 |
| Num. H-bond acceptors | 3 |
| Num. H-bond donors | 1 |
| Molar Refractivity | 124.15 |
| TPSA | 55.37 Å² |

| Lipophilicity | |
|---|---|
| Log $P_{o/w}$ (iLOGP) | 4.21 |
| Log $P_{o/w}$ (XLOGP3) | 6.12 |
| Log $P_{o/w}$ (WLOGP) | 5.36 |
| Log $P_{o/w}$ (MLOGP) | 5.01 |
| Log $P_{o/w}$ (SILICOS-IT) | 5.12 |
| Consensus Log $P_{o/w}$ | 5.16 |

| Water Solubility | |
|---|---|
| Log *S* (ESOL) | -6.51 |
| Solubility | 1.29e-04 mg/ml ; 3.09e-07 mol/l |
| Class | Poorly soluble |
| Log *S* (Ali) | -7.06 |
| Solubility | 3.60e-05 mg/ml ; 8.62e-08 mol/l |
| Class | Poorly soluble |
| Log *S* (SILICOS-IT) | -7.14 |
| Solubility | 3.04e-05 mg/ml ; 7.28e-08 mol/l |
| Class | Poorly soluble |

| Pharmacokinetics | |
|---|---|
| GI absorption | High |
| BBB permeant | Yes |
| P-gp substrate | No |
| CYP1A2 inhibitor | No |
| CYP2C19 inhibitor | No |
| CYP2C9 inhibitor | Yes |
| CYP2D6 inhibitor | Yes |
| CYP3A4 inhibitor | No |
| Log $K_p$ (skin permeation) | -4.50 cm/s |

| Druglikeness | |
|---|---|
| Lipinski | Yes; 1 violation: MLOGP>4.15 |
| Ghose | Yes |
| Veber | Yes |
| Egan | Yes |
| Muegge | No; 1 violation: XLOGP3>5 |
| Bioavailability Score | 0.55 |

| Medicinal Chemistry | |
|---|---|
| PAINS | 0 alert |
| Brenk | 0 alert |
| Leadlikeness | No; 2 violations: MW>350, XLOGP3>3.5 |
| Synthetic accessibility | 5.19 |

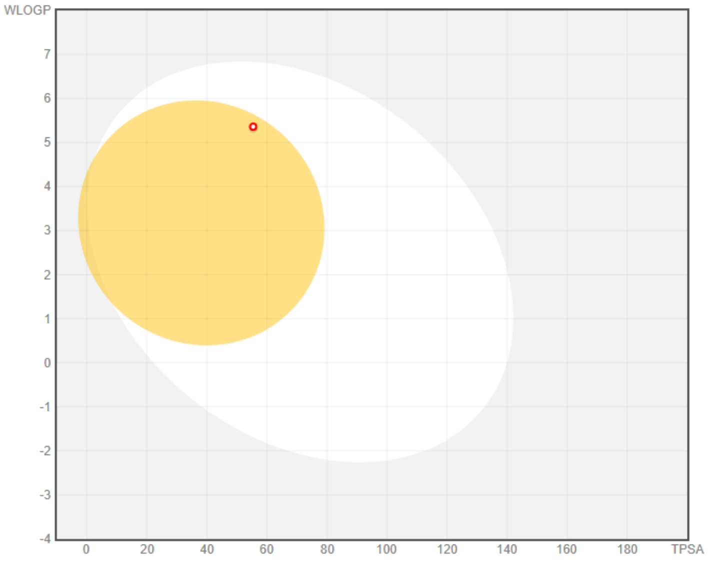


**Actions**

☐ Show Molecules Name

**Legends**

BBB
HIA
PGP+
PGP-

**Remarks**

None

Molecule 11: compound **8f**

## Molecule 12

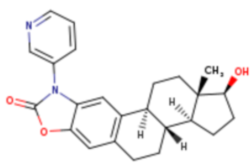

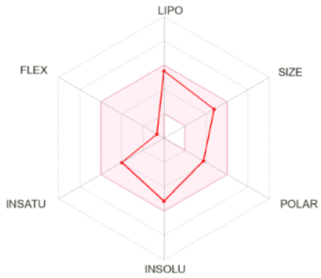


SMILES O[C@H]1CC[C@@H]2[C@]1(C)CC[C@H]1[C@H]2CCc2c1cc1c(c2)oc(=O)n1c1cccnc1

| Physicochemical Properties | |
|---|---|
| Formula | C24H26N2O3 |
| Molecular weight | 390.47 g/mol |
| Num. heavy atoms | 29 |
| Num. arom. heavy atoms | 15 |
| Fraction Csp3 | 0.50 |
| Num. rotatable bonds | 1 |
| Num. H-bond acceptors | 4 |
| Num. H-bond donors | 1 |
| Molar Refractivity | 112.17 |
| TPSA | 68.26 Å² |

| Lipophilicity | |
|---|---|
| Log $P_{o/w}$ (iLOGP) | 3.35 |
| Log $P_{o/w}$ (XLOGP3) | 4.15 |
| Log $P_{o/w}$ (WLOGP) | 4.20 |
| Log $P_{o/w}$ (MLOGP) | 3.56 |
| Log $P_{o/w}$ (SILICOS-IT) | 3.64 |
| Consensus Log $P_{o/w}$ | 3.78 |

| Water Solubility | |
|---|---|
| Log *S* (ESOL) | -5.19 |
| Solubility | 2.51e-03 mg/ml ; 6.42e-06 mol/l |
| Class | Moderately soluble |
| Log *S* (Ali) | -5.29 |
| Solubility | 2.00e-03 mg/ml ; 5.12e-06 mol/l |
| Class | Moderately soluble |
| Log *S* (SILICOS-IT) | -6.00 |
| Solubility | 3.93e-04 mg/ml ; 1.01e-06 mol/l |
| Class | Moderately soluble |

| Pharmacokinetics | |
|---|---|
| GI absorption | High |
| BBB permeant | Yes |
| P-gp substrate | No |
| CYP1A2 inhibitor | No |
| CYP2C19 inhibitor | Yes |
| CYP2C9 inhibitor | No |
| CYP2D6 inhibitor | Yes |
| CYP3A4 inhibitor | No |
| Log $K_p$ (skin permeation) | -5.74 cm/s |

| Druglikeness | |
|---|---|
| Lipinski | Yes; 0 violation |
| Ghose | Yes |
| Veber | Yes |
| Egan | Yes |
| Muegge | Yes |
| Bioavailability Score | 0.55 |

| Medicinal Chemistry | |
|---|---|
| PAINS | 0 alert |
| Brenk | 0 alert |
| Leadlikeness | No; 2 violations: MW>350, XLOGP3>3.5 |
| Synthetic accessibility | 5.06 |

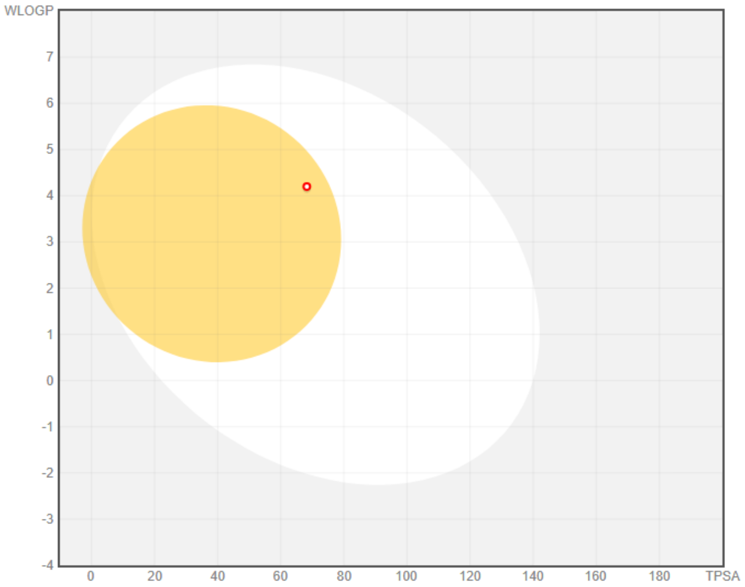


**Actions**

☐ Show Molecules Name

**Legends**

- BBB
- HIA
- PGP+
- PGP-

**Remarks**

None

Molecule 12: compound **8g**

## Molecule 13

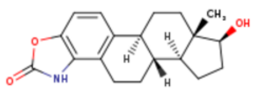


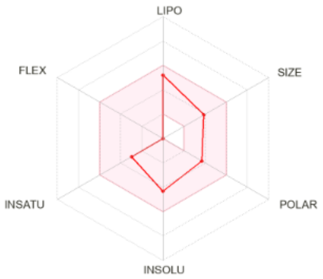


SMILES O[C@H]1CC[C@@H]2[C@]1(C)CC[C@H]1[C@H]2CCc2c1ccc1c2[nH]c(=O)o1

| Physicochemical Properties | |
|---|---|
| Formula | C19H23NO3 |
| Molecular weight | 313.39 g/mol |
| Num. heavy atoms | 23 |
| Num. arom. heavy atoms | 9 |
| Fraction Csp3 | 0.63 |
| Num. rotatable bonds | 0 |
| Num. H-bond acceptors | 3 |
| Num. H-bond donors | 2 |
| Molar Refractivity | 89.40 |
| TPSA | 66.23 Å² |

| Lipophilicity | |
|---|---|
| Log $P_{o/w}$ (iLOGP) | 2.76 |
| Log $P_{o/w}$ (XLOGP3) | 3.58 |
| Log $P_{o/w}$ (WLOGP) | 3.34 |
| Log $P_{o/w}$ (MLOGP) | 3.26 |
| Log $P_{o/w}$ (SILICOS-IT) | 3.71 |
| Consensus Log $P_{o/w}$ | 3.33 |

| Water Solubility | |
|---|---|
| Log *S* (ESOL) | -4.33 |
| Solubility | 1.47e-02 mg/ml ; 4.70e-05 mol/l |
| Class | Moderately soluble |
| Log *S* (Ali) | -4.66 |
| Solubility | 6.90e-03 mg/ml ; 2.20e-05 mol/l |
| Class | Moderately soluble |
| Log *S* (SILICOS-IT) | -4.73 |
| Solubility | 5.82e-03 mg/ml ; 1.86e-05 mol/l |
| Class | Moderately soluble |

| Pharmacokinetics | |
|---|---|
| GI absorption | High |
| BBB permeant | Yes |
| P-gp substrate | Yes |
| CYP1A2 inhibitor | No |
| CYP2C19 inhibitor | No |
| CYP2C9 inhibitor | No |
| CYP2D6 inhibitor | Yes |
| CYP3A4 inhibitor | No |
| Log $K_p$ (skin permeation) | -5.67 cm/s |

| Druglikeness | |
|---|---|
| Lipinski | Yes; 0 violation |
| Ghose | Yes |
| Veber | Yes |
| Egan | Yes |
| Muegge | Yes |
| Bioavailability Score | 0.55 |

| Medicinal Chemistry | |
|---|---|
| PAINS | 0 alert |
| Brenk | 0 alert |
| Leadlikeness | No; 1 violation: XLOGP3>3.5 |
| Synthetic accessibility | 4.54 |

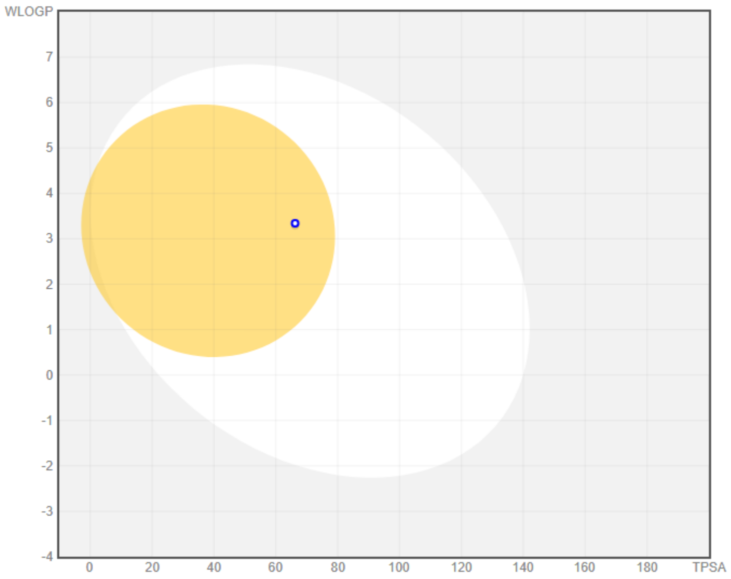


| Actions |
|---|
| ☐ Show Molecules Name |

| Legends |
|---|
| BBB |
| HIA |
| PGP+ |
| PGP- |

| Remarks |
|---|
| None |

Molecule 13: compound **7a**

## Molecule 14

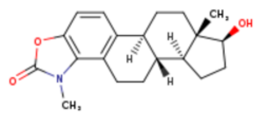


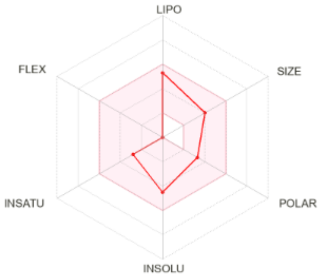


SMILES O[C@H]1CC[C@@H]2[C@]1(C)CC[C@H]1[C@H]2CCc2c1ccc1c2n(C)c(=O)o1

| Physicochemical Properties | |
|---|---|
| Formula | C20H25NO3 |
| Molecular weight | 327.42 g/mol |
| Num. heavy atoms | 24 |
| Num. arom. heavy atoms | 9 |
| Fraction Csp3 | 0.65 |
| Num. rotatable bonds | 0 |
| Num. H-bond acceptors | 3 |
| Num. H-bond donors | 1 |
| Molar Refractivity | 94.30 |
| TPSA | 55.37 Å² |

| Lipophilicity | |
|---|---|
| Log $P_{o/w}$ (iLOGP) | 3.13 |
| Log $P_{o/w}$ (XLOGP3) | 3.76 |
| Log $P_{o/w}$ (WLOGP) | 3.35 |
| Log $P_{o/w}$ (MLOGP) | 3.49 |
| Log $P_{o/w}$ (SILICOS-IT) | 3.18 |
| Consensus Log $P_{o/w}$ | 3.38 |

| Water Solubility | |
|---|---|
| Log *S* (ESOL) | -4.52 |
| Solubility | 9.97e-03 mg/ml ; 3.05e-05 mol/l |
| Class | Moderately soluble |
| Log *S* (Ali) | -4.62 |
| Solubility | 7.93e-03 mg/ml ; 2.42e-05 mol/l |
| Class | Moderately soluble |
| Log *S* (SILICOS-IT) | -4.29 |
| Solubility | 1.70e-02 mg/ml ; 5.19e-05 mol/l |
| Class | Moderately soluble |

| Pharmacokinetics | |
|---|---|
| GI absorption | High |
| BBB permeant | Yes |
| P-gp substrate | Yes |
| CYP1A2 inhibitor | No |
| CYP2C19 inhibitor | No |
| CYP2C9 inhibitor | No |
| CYP2D6 inhibitor | Yes |
| CYP3A4 inhibitor | No |
| Log $K_p$ (skin permeation) | -5.63 cm/s |

| Druglikeness | |
|---|---|
| Lipinski | Yes; 0 violation |
| Ghose | Yes |
| Veber | Yes |
| Egan | Yes |
| Muegge | Yes |
| Bioavailability Score | 0.55 |

| Medicinal Chemistry | |
|---|---|
| PAINS | 0 alert |
| Brenk | 0 alert |
| Leadlikeness | No; 1 violation: XLOGP3>3.5 |
| Synthetic accessibility | 4.68 |

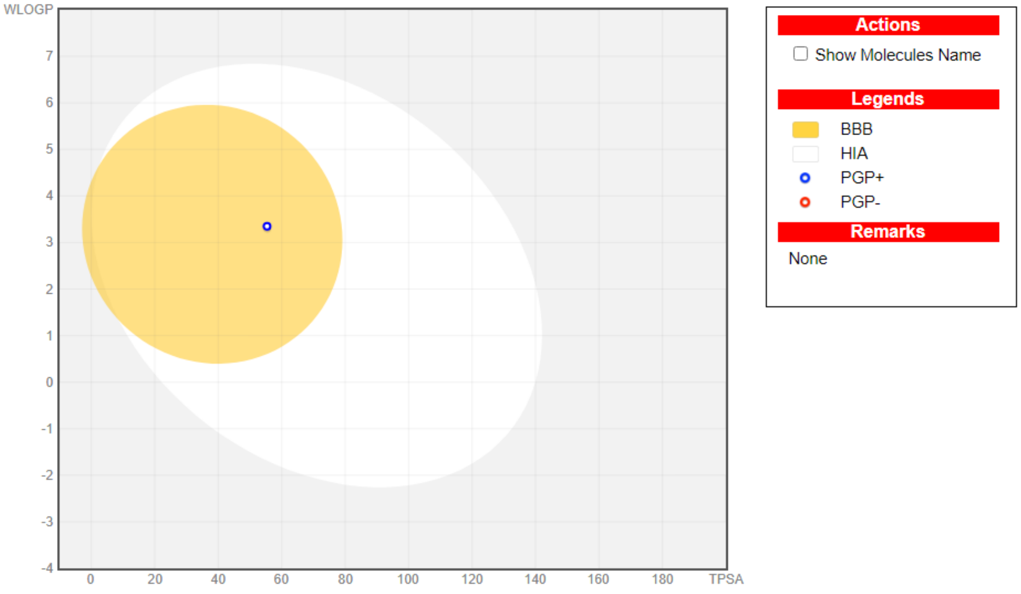


Molecule 14: Compound **7b**

## Molecule 15

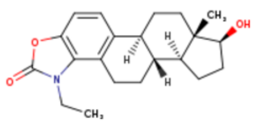


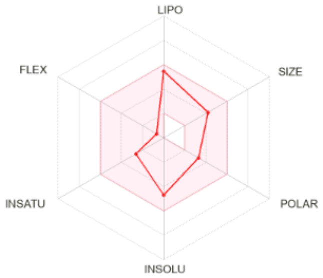


SMILES CCn1c(=O)oc2c1cc1CC[C@@H]3[C@@H](c1cc2)CC[C@]1([C@H]3CC[C@@H]1O)C

| Physicochemical Properties | |
|---|---|
| Formula | C21H27NO3 |
| Molecular weight | 341.44 g/mol |
| Num. heavy atoms | 25 |
| Num. arom. heavy atoms | 9 |
| Fraction Csp3 | 0.67 |
| Num. rotatable bonds | 1 |
| Num. H-bond acceptors | 3 |
| Num. H-bond donors | 1 |
| Molar Refractivity | 99.10 |
| TPSA | 55.37 Å² |

| Lipophilicity | |
|---|---|
| Log $P_{o/w}$ (iLOGP) | 3.33 |
| Log $P_{o/w}$ (XLOGP3) | 4.03 |
| Log $P_{o/w}$ (WLOGP) | 3.83 |
| Log $P_{o/w}$ (MLOGP) | 3.71 |
| Log $P_{o/w}$ (SILICOS-IT) | 3.56 |
| Consensus Log $P_{o/w}$ | 3.69 |

| Water Solubility | |
|---|---|
| Log *S* (ESOL) | -4.70 |
| Solubility | 6.87e-03 mg/ml ; 2.01e-05 mol/l |
| Class | Moderately soluble |
| Log *S* (Ali) | -4.90 |
| Solubility | 4.34e-03 mg/ml ; 1.27e-05 mol/l |
| Class | Moderately soluble |
| Log *S* (SILICOS-IT) | -4.68 |
| Solubility | 7.12e-03 mg/ml ; 2.08e-05 mol/l |
| Class | Moderately soluble |

| Pharmacokinetics | |
|---|---|
| GI absorption | High |
| BBB permeant | Yes |
| P-gp substrate | Yes |
| CYP1A2 inhibitor | No |
| CYP2C19 inhibitor | No |
| CYP2C9 inhibitor | No |
| CYP2D6 inhibitor | Yes |
| CYP3A4 inhibitor | No |
| Log $K_p$ (skin permeation) | -5.52 cm/s |

| Druglikeness | |
|---|---|
| Lipinski | Yes; 0 violation |
| Ghose | Yes |
| Veber | Yes |
| Egan | Yes |
| Muegge | Yes |
| Bioavailability Score | 0.55 |

| Medicinal Chemistry | |
|---|---|
| PAINS | 0 alert |
| Brenk | 0 alert |
| Leadlikeness | No; 1 violation: XLOGP3>3.5 |
| Synthetic accessibility | 4.85 |

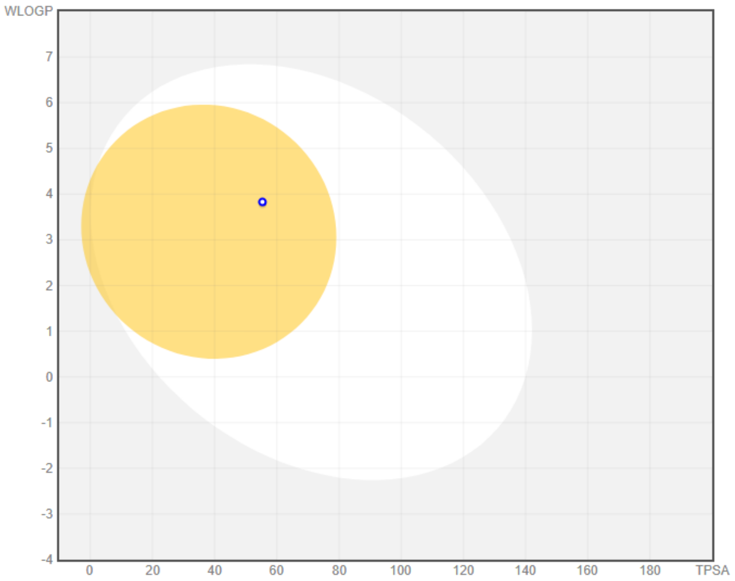


**Actions**

☐ Show Molecules Name

**Legends**

BBB
HIA
PGP+
PGP-

**Remarks**

None

Molecule 15: compound **7c**

## Molecule 16

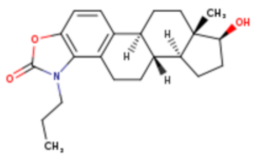


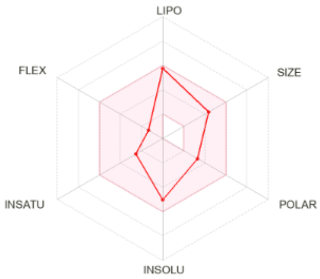


SMILES C[C@]12CC[C@H]3[C@H]([C@@H]1CC[C@@H]2O)CCc1c3ccc2c1n(CCC)c(=O)o2

| Physicochemical Properties | |
|---|---|
| Formula | C22H29NO3 |
| Molecular weight | 355.47 g/mol |
| Num. heavy atoms | 26 |
| Num. arom. heavy atoms | 9 |
| Fraction Csp3 | 0.68 |
| Num. rotatable bonds | 2 |
| Num. H-bond acceptors | 3 |
| Num. H-bond donors | 1 |
| Molar Refractivity | 103.91 |
| TPSA | 55.37 Å² |

| Lipophilicity | |
|---|---|
| Log $P_{o/w}$ (iLOGP) | 3.57 |
| Log $P_{o/w}$ (XLOGP3) | 4.55 |
| Log $P_{o/w}$ (WLOGP) | 4.22 |
| Log $P_{o/w}$ (MLOGP) | 3.93 |
| Log $P_{o/w}$ (SILICOS-IT) | 3.94 |
| Consensus Log $P_{o/w}$ | 4.04 |

| Water Solubility | |
|---|---|
| Log *S* (ESOL) | -5.03 |
| Solubility | 3.28e-03 mg/ml ; 9.23e-06 mol/l |
| Class | Moderately soluble |
| Log *S* (Ali) | -5.44 |
| Solubility | 1.30e-03 mg/ml ; 3.67e-06 mol/l |
| Class | Moderately soluble |
| Log *S* (SILICOS-IT) | -5.08 |
| Solubility | 2.98e-03 mg/ml ; 8.39e-06 mol/l |
| Class | Moderately soluble |

| Pharmacokinetics | |
|---|---|
| GI absorption | High |
| BBB permeant | Yes |
| P-gp substrate | No |
| CYP1A2 inhibitor | No |
| CYP2C19 inhibitor | Yes |
| CYP2C9 inhibitor | Yes |
| CYP2D6 inhibitor | Yes |
| CYP3A4 inhibitor | No |
| Log $K_p$ (skin permeation) | -5.24 cm/s |

| Druglikeness | |
|---|---|
| Lipinski | Yes; 0 violation |
| Ghose | Yes |
| Veber | Yes |
| Egan | Yes |
| Muegge | Yes |
| Bioavailability Score | 0.55 |

| Medicinal Chemistry | |
|---|---|
| PAINS | 0 alert |
| Brenk | 0 alert |
| Leadlikeness | No; 2 violations: MW>350, XLOGP3>3.5 |
| Synthetic accessibility | 4.99 |

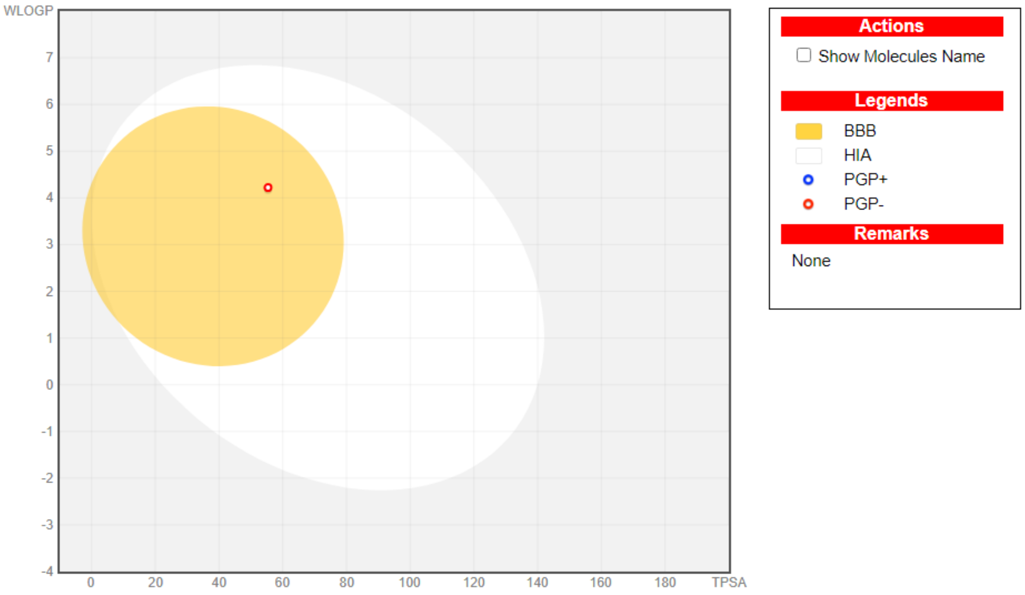


Molecules 16: compound **7d**

## Molecule 17

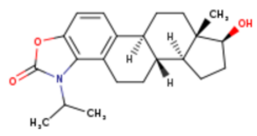

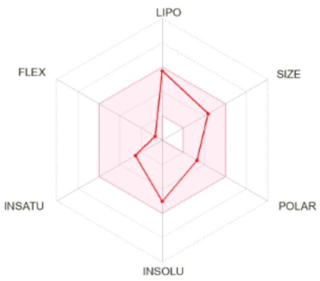


SMILES O[C@H]1CC[C@@H]2[C@]1(C)CC[C@H]1[C@H]2CCc2c1ccc1c2n(C(C)C)c(=O)o1

| Physicochemical Properties | |
|---|---|
| Formula | C22H29NO3 |
| Molecular weight | 355.47 g/mol |
| Num. heavy atoms | 26 |
| Num. arom. heavy atoms | 9 |
| Fraction Csp3 | 0.68 |
| Num. rotatable bonds | 1 |
| Num. H-bond acceptors | 3 |
| Num. H-bond donors | 1 |
| Molar Refractivity | 103.91 |
| TPSA | 55.37 Å² |

| Lipophilicity | |
|---|---|
| Log $P_{o/w}$ (iLOGP) | 3.55 |
| Log $P_{o/w}$ (XLOGP3) | 4.46 |
| Log $P_{o/w}$ (WLOGP) | 4.39 |
| Log $P_{o/w}$ (MLOGP) | 3.93 |
| Log $P_{o/w}$ (SILICOS-IT) | 3.77 |
| Consensus Log $P_{o/w}$ | 4.02 |

| Water Solubility | |
|---|---|
| Log *S* (ESOL) | -5.04 |
| Solubility | 3.21e-03 mg/ml ; 9.04e-06 mol/l |
| Class | Moderately soluble |
| Log *S* (Ali) | -5.34 |
| Solubility | 1.62e-03 mg/ml ; 4.55e-06 mol/l |
| Class | Moderately soluble |
| Log *S* (SILICOS-IT) | -4.70 |
| Solubility | 7.04e-03 mg/ml ; 1.98e-05 mol/l |
| Class | Moderately soluble |

| Pharmacokinetics | |
|---|---|
| GI absorption | High |
| BBB permeant | Yes |
| P-gp substrate | No |
| CYP1A2 inhibitor | No |
| CYP2C19 inhibitor | No |
| CYP2C9 inhibitor | Yes |
| CYP2D6 inhibitor | Yes |
| CYP3A4 inhibitor | No |
| Log $K_p$ (skin permeation) | -5.30 cm/s |

| Druglikeness | |
|---|---|
| Lipinski | Yes; 0 violation |
| Ghose | Yes |
| Veber | Yes |
| Egan | Yes |
| Muegge | Yes |
| Bioavailability Score | 0.55 |

| Medicinal Chemistry | |
|---|---|
| PAINS | 0 alert |
| Brenk | 0 alert |
| Leadlikeness | No; 2 violations: MW>350, XLOGP3>3.5 |
| Synthetic accessibility | 4.97 |

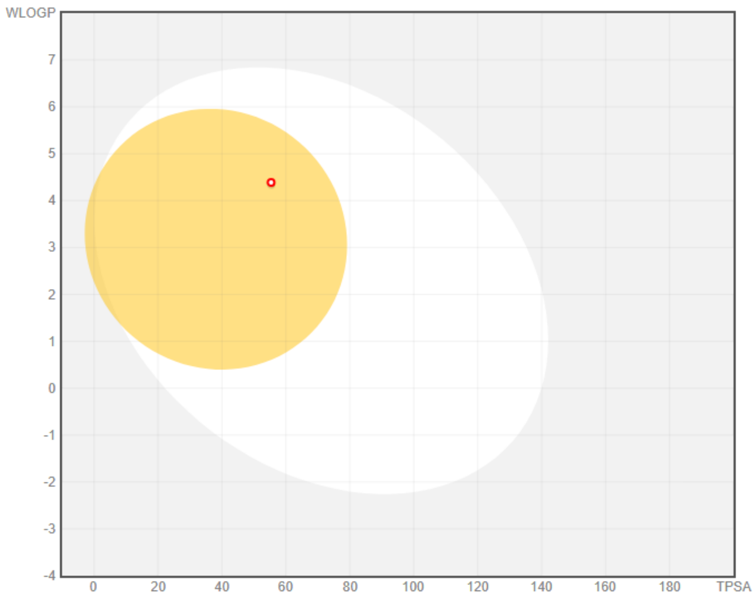


**Actions**

☐ Show Molecules Name

**Legends**

BBB
HIA
PGP+
PGP-

**Remarks**

None

Molecules 17: compound **7e**

**Table S1.** Selected physicochemical properties, lipophilicity (logP) and water solubility (logS) of the synthesized molecules computed by the SwissADME web tool

| Compd | Physicochemical properties | | | | Lipophilicity | | | Water solubility | | |
|---|---|---|---|---|---|---|---|---|---|---|
| | MW (g/mol) | Fraction $Csp^3$ | #rotatable bonds | TPSA ($Å^2$) | $logP_{o/w}$ (XLOGP3) | $LogP_{o/w}$ (MLOGP) | Consensus $logP_{o/w}$ | logS (ESOL) | logS (Ali) | logS (SILICOS-IT) |
| **6a** | 313.39 | 0.63 | 0 | 66.23 | 3.58 | 3.26 | 3.33 | -4.33 | -4.66 | -4.73 |
| **6b** | 327.42 | 0.65 | 0 | 55.37 | 3.76 | 3.49 | 3.40 | -4.52 | -4.62 | -4.29 |
| **6c** | 341.44 | 0.67 | 1 | 55.37 | 4.13 | 3.71 | 3.74 | -4.76 | -5.00 | -4.68 |
| **6d** | 355.47 | 0.68 | 2 | 55.37 | 4.66 | 3.93 | 4.09 | -5.10 | -5.55 | -5.08 |
| **6e** | 355.47 | 0.68 | 1 | 55.37 | 4.56 | 3.93 | 4.06 | -5.11 | -5.45 | -4.70 |
| **8a** | 389.49 | 0.48 | 1 | 55.37 | 5.32 | 4.60 | 4.53 | -5.92 | -6.23 | -6.37 |
| **8b** | 407.48 | 0.48 | 1 | 55.37 | 5.42 | 4.97 | 4.85 | -6.08 | -6.34 | -6.63 |
| **8c** | 423.93 | 0.48 | 1 | 55.37 | 5.95 | 5.07 | 5.07 | -6.52 | -6.89 | -6.96 |
| **8d** | 468.38 | 0.48 | 1 | 55.37 | 6.01 | 5.17 | 5.15 | -6.83 | -6.95 | -7.15 |
| **8e** | 414.50 | 0.46 | 1 | 79.16 | 5.04 | 3.90 | 4.30 | -5.88 | -6.44 | -6.43 |
| **8f** | 417.54 | 0.52 | 2 | 55.37 | 6.12 | 5.01 | 5.16 | -6.51 | -7.06 | -7.14 |
| **8g** | 390.47 | 0.50 | 1 | 68.26 | 4.15 | 3.56 | 3.78 | -5.19 | -5.29 | -6.00 |
| **7a** | 313.39 | 0.63 | 0 | 66.23 | 3.58 | 3.26 | 3.33 | -4.33 | -4.66 | -4.73 |
| **7b** | 327.42 | 0.65 | 0 | 55.37 | 3.76 | 3.49 | 3.38 | -4.52 | -4.62 | -4.29 |
| **7c** | 341.44 | 0.67 | 1 | 55.37 | 4.03 | 3.71 | 3.69 | -4.70 | -4.90 | -4.68 |
| **7d** | 355.47 | 0.68 | 2 | 55.37 | 4.55 | 3.93 | 4.04 | -5.03 | -5.44 | -5.08 |
| **7e** | 355.47 | 0.68 | 1 | 55.37 | 4.46 | 3.93 | 4.02 | -5.04 | -5.34 | -4.70 |

MW: molecular weight, TPSA; total polar surface area [S1]; XLOGP3: atomistic and knowledge-based method calculated by XLOGP program, version 3.2.2, courtesy of CCBG, Shanghai Institute of Organic Chemistry; MLOGP: topological method [S2]; Consensus $logP_{o/w}$: average values of five LogP predictions including XLOGP3, MLOGP and three others (ILOGP [32], WLOGP [S3] and SILICOS-IT [https://www.silicos-it.be/] evaluated upon various lipophilicity criteria; water solubility: lgS (ESOL) [S4]; lgS (Ali) [S5], logS (SILICOS-IT) [https://www.silicos-it.be/].

[S1] P. Ertl, B. Rohde, P. Selzer, Fast calculation of molecular polar surface area as a sum of fragment-based contributions and its application to the prediction of drug transport properties, *J. Med Chem.* 43 (2000) 3714-3717. doi: 10.1021/jm000942e.

[S2] P. A. Lipinski, F- Lombardo, B. W. Dominy, P. J. Feeney, Experimental and computational approaches to estimate solubility and permeability in drug discovery and development settings, *Adv. Drug. Deliv. Rev*. 46 (2001) 3-26. doi: 10.1016/s0169-409x(00)00129-0

[S3] S. A. Wildman, G. M. Crippen, Prediction of physicochemical parameters by atomic contributions, *Chem. Inf. Comput. Sci.* 39 (1999) 868–873. doi:10.1021/ci990307l

[S4] J. S. Delaney, ESOL: estimating aqueous solubility directly from molecular structure, *J. Chem. Inf. Comput. Sci.* 44 (2004) 1000-1005. doi: 10.1021/ci034243x.

[S5] J. Ali, P. Camilleri, M. B. Brown, A. J. Hutt, S. B. Kirton, *In silico* prediction of aqueous solubility using simple QSPR models: the importance of phenol and phenol-like moieties. *J. Chem. Inf. Model* 52 (2012) 2950-2957. doi: 10.1021/ci300447c.

**Table S2.** Evaluation of drug-likeness and selected pharmacokinetic properties of the synthesized molecules using swissADME (GI: gastrointestinal tract, BBB: blood brain barrier, P-gp: P-glycoprotein, which is expressed in both the intestinal epithelium and the capillary endothelial cells composing the BBB, pumping xenobiotics back into the intestinal lumen and into the capillaries, respectively)

| Compd | Lipinski violations | Ghose violations | Veber violations | Egan violations | Muegge violations | GI absorption | BBB penetration | P-gp substrate |
|---|---|---|---|---|---|---|---|---|
| **6a** | 0 | 0 | 0 | 0 | 0 | high | yes | yes |
| **6b** | 0 | 0 | 0 | 0 | 0 | high | yes | yes |
| **6c** | 0 | 0 | 0 | 0 | 0 | high | yes | no |
| **6d** | 0 | 0 | 0 | 0 | 0 | high | yes | no |
| **6e** | 0 | 0 | 0 | 0 | 0 | high | yes | no |
| **8a** | 1 | 0 | 0 | 0 | 1 | high | yes | no |
| **8b** | 1 | 0 | 0 | 0 | 1 | high | yes | no |
| **8c** | 1 | 0 | 0 | 0 | 1 | high | yes | no |
| **8d** | 1 | 0 | 0 | 0 | 1 | high | yes | no |
| **8e** | 0 | 0 | 0 | 0 | 1 | high | no | no |
| **8f** | 1 | 0 | 0 | 0 | 1 | high | yes | no |
| **8g** | 0 | 0 | 0 | 0 | 0 | high | yes | no |
| **7a** | 0 | 0 | 0 | 0 | 0 | high | yes | yes |
| **7b** | 0 | 0 | 0 | 0 | 0 | high | yes | yes |
| **7c** | 0 | 0 | 0 | 0 | 0 | high | yes | yes |
| **7d** | 0 | 0 | 0 | 0 | 0 | high | yes | no |
| **7e** | 0 | 0 | 0 | 0 | 0 | high | yes | no |

Lipinski (Pfizer) filter [S2]: MW ≤ 4.15, N or O ≤ 10, NH or OH ≤ 5; Ghose filter [S6]: 160 ≤ MW ≤ 480, -0.4 ≤ WLOGP ≤ 5.6, 40 ≤ MR ≤ 130, 20 ≤ atoms ≤ 70; Veber (GSK) filter [S7]: rotatable bonds ≤ 10, TPSA ≤ 140; Egan (Pharmacia) filter [S8]: WLOGP ≤ 5.88, TPSA ≤ 131.6; Muegge (Bayer) filter [S9]: 200 ≤ MW ≤ 600, -2 ≤ XLOGP ≤ 5, TPSA ≤ 150, num. rings ≤ 7, num. carbon > 4, num. heteroatoms > 1, num. rotatable bonds ≤ 15, H-bond acc. < 10, H-bond don. < 5

[S6] A. K. Ghose, V. N. Viswanadhan, J. J. Wendoloski, A knowledge-based approach in designing combinatorial or medicinal chemistry libraries for drug discovery. 1. A qualitative and quantitative characterization of known drug databases, J. Comb. Chem. 1 (1999) 55-68. doi: 10.1021/cc9800071

[S7] D. F. Veber, S. R. Johnson, H. Y. Cheng, B. R. Smith, K. W. Ward, K. D. Kopple, Molecular properties that influence the oral bioavailability of drug candidates, *J. Med. Chem*. 45 (2002) 2615-2623. doi: 10.1021/jm020017n.

[S8] W. J. Egan, K. M. Jr. Merz, J. J. Baldwin, Prediction of drug absorption using multivariate statistics, *J. Med. Chem.* 43 (2000) 3867-3877. doi: 10.1021/jm000292e.

[S9] I. Muegge, S. L. Heald, D. Brittelli, Simple selection criteria for drug-like chemical matter, *J. Med. Chem*. 44 (2001) 1841-1846. doi: 10.1021/jm015507e.

**Table S3**. Values of primary growth inhibitory screen used for heat map construction. Mean ± SD values of primary growth inhibitory screen (given as cell viability) used for heat map construction. The compounds were tested in 2.5 μM and in 5.0 μM concentration for 72 h. Control represents viability of cells receiving solvent (DMSO) treatment.

| 5 mM | A549 | | Du-145 | | HeLa | | MCF-7 | | MRC-5 | |
|---|---|---|---|---|---|---|---|---|---|---|
| | mean (%) | ±SD | mean (%) | ±SD | mean (%) | ±SD | mean (%) | ±SD | mean (%) | ±SD |
| control | 100,00 | 2,72 | 100,00 | 6,84 | 100,00 | 5,99 | 100,00 | 7,66 | 100,00 | 7,69 |
| **6b** | 82,06 | 0,77 | 84,28 | 3,71 | 4,25 | 0,38 | 77,63 | 2,40 | 93,79 | 4,50 |
| **6c** | 35,21 | 2,98 | 53,34 | 3,26 | 2,91 | 0,21 | 22,21 | 2,27 | 99,21 | 6,52 |
| **6d** | 53,33 | 6,63 | 32,24 | 5,88 | 32,42 | 4,90 | 76,81 | 10,05 | 117,38 | 5,08 |
| **7b** | 64,05 | 1,69 | 71,00 | 7,29 | 69,59 | 4,05 | 59,25 | 5,28 | 104,91 | 7,14 |
| **7c** | 72,75 | 3,56 | 81,91 | 3,96 | 41,87 | 3,57 | 51,73 | 7,40 | 96,52 | 3,45 |
| **7d** | 64,83 | 6,70 | 48,93 | 5,00 | 65,99 | 4,09 | 72,96 | 2,95 | 115,01 | 6,24 |
| **7e** | 68,17 | 1,68 | 82,22 | 0,86 | 56,34 | 3,67 | 79,77 | 7,56 | 108,59 | 8,75 |
| **8a** | 74,48 | 3,30 | 75,09 | 2,05 | 43,76 | 2,84 | 67,61 | 3,08 | 95,02 | 6,22 |
| **8b** | 78,56 | 3,78 | 84,94 | 2,99 | 82,42 | 3,34 | 103,04 | 12,58 | 121,52 | 5,00 |
| **8c** | 66,63 | 4,97 | 75,57 | 3,08 | 85,97 | 3,12 | 98,66 | 9,18 | 129,35 | 5,34 |
| **8d** | 71,02 | 4,63 | 74,87 | 6,59 | 86,74 | 2,27 | 101,97 | 11,13 | 117,59 | 12,59 |
| **8e** | 76,35 | 4,50 | 75,69 | 4,56 | 75,31 | 1,16 | 100,54 | 4,41 | 115,16 | 2,21 |
| **8f** | 74,34 | 4,59 | 73,98 | 4,15 | 81,23 | 4,22 | 111,64 | 10,52 | 123,01 | 10,07 |
| **8g** | 74,97 | 5,74 | 90,45 | 3,34 | 88,86 | 2,77 | 112,71 | 13,74 | 113,92 | 8,36 |

| 2,5 mM | A549 | | Du-145 | | HeLa | | MCF-7 | | MRC-5 | |
|---|---|---|---|---|---|---|---|---|---|---|
| | mean (%) | ±SD | mean (%) | ±SD | mean (%) | ±SD | mean (%) | ±SD | mean (%) | ±SD |
| control | 100,00 | 4,44 | 100,00 | 4,92 | 100,00 | 5,15 | 99,66 | 6,25 | 99,46 | 4,76 |
| **6b** | 89,15 | 2,35 | 99,48 | 6,83 | 39,56 | 4,70 | 107,76 | 5,18 | 97,83 | 9,54 |
| **6c** | 73,84 | 4,11 | 73,12 | 7,21 | 21,00 | 1,99 | 65,61 | 7,16 | 103,76 | 9,00 |
| **6d** | 81,89 | 2,22 | 60,43 | 1,35 | 37,20 | 1,80 | 97,73 | 9,84 | 100,06 | 3,90 |
| **7b** | 72,89 | 7,45 | 77,29 | 6,27 | 48,85 | 3,76 | 74,68 | 11,57 | 115,00 | 6,72 |
| **7c** | 84,83 | 7,02 | 105,94 | 9,10 | 60,29 | 7,74 | 112,96 | 7,33 | 111,08 | 9,37 |
| **7d** | 86,04 | 4,44 | 78,86 | 6,14 | 71,79 | 4,49 | 142,93 | 10,74 | 92,11 | 17,15 |
| **7e** | 99,55 | 4,74 | 83,51 | 6,22 | 91,70 | 10,35 | 101,26 | 15,95 | 98,60 | 4,67 |
| **8a** | 79,22 | 5,14 | 85,54 | 6,93 | 75,42 | 3,31 | 105,24 | 5,30 | 108,51 | 10,71 |
| **8b** | 92,72 | 5,15 | 72,72 | 6,21 | 83,25 | 9,58 | 156,40 | 9,79 | 102,25 | 5,74 |
| **8c** | 98,27 | 6,51 | 69,64 | 7,05 | 56,39 | 9,46 | 125,56 | 14,81 | 99,05 | 5,64 |
| **8d** | 103,21 | 4,63 | 81,10 | 4,43 | 84,91 | 3,56 | 186,32 | 12,07 | 95,63 | 2,32 |
| **8e** | 91,66 | 4,04 | 78,75 | 4,73 | 76,13 | 8,99 | 109,99 | 8,00 | 101,95 | 8,67 |
| **8f** | 81,13 | 4,44 | 64,41 | 9,76 | 58,24 | 5,10 | 118,95 | 11,20 | 95,53 | 0,37 |
| **8g** | 93,40 | 4,57 | 77,81 | 9,73 | 84,25 | 4,89 | 182,13 | 10,76 | 94,02 | 3,82 |

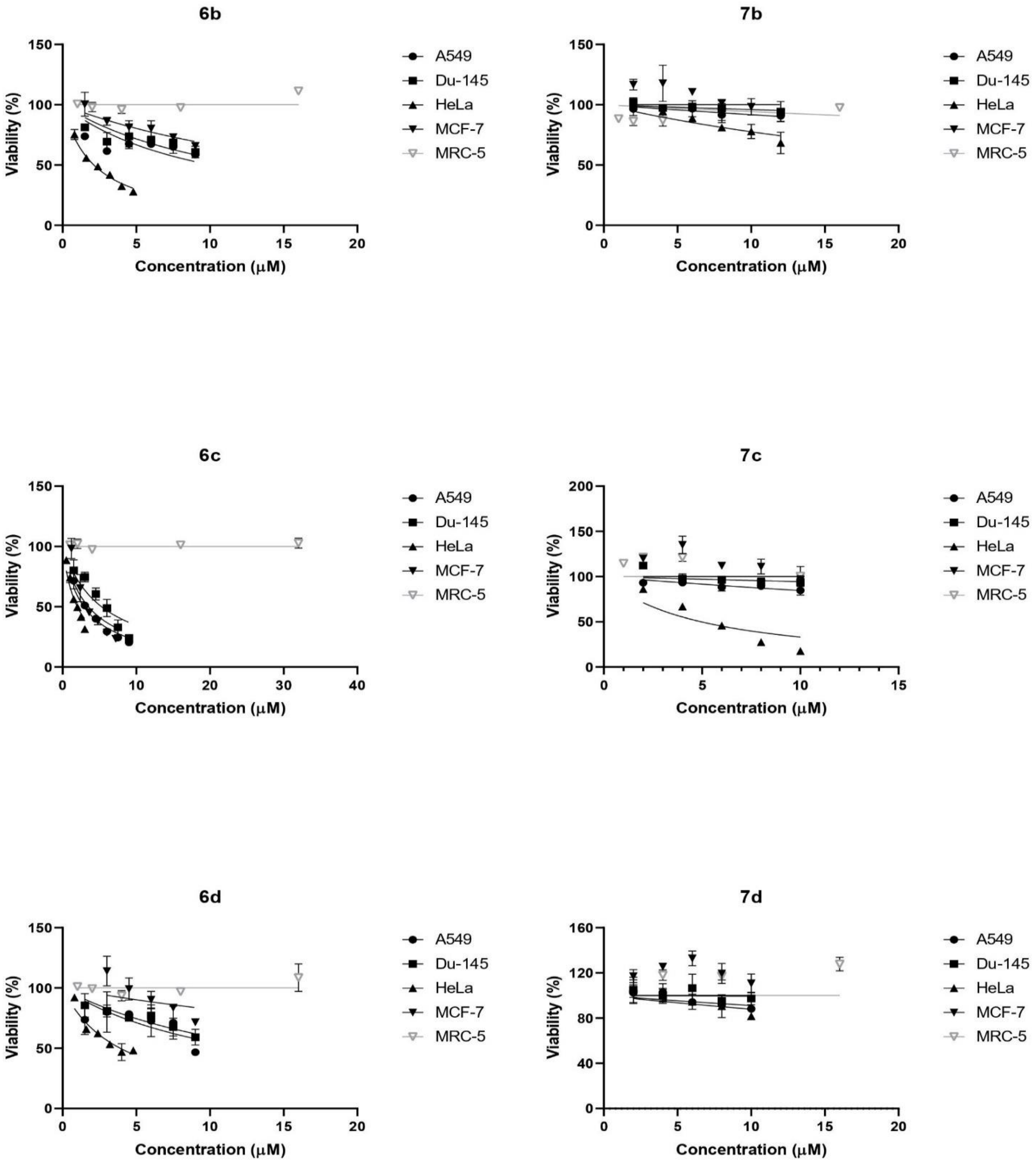


**Fig. S1**. Representative cell viability curves to determine growth inhibition and $IC_{50}$ values following treatments with the selected compounds on different cell lines. On X-axis the applied concentrations are represented in μM. Based on the viability data. $IC_{50}$ values were obtained. and represented in Table 3.
